# Molecular Criteria for Defining the Naive Human Pluripotent State

**DOI:** 10.1016/j.stem.2016.06.011

**Published:** 2016-10-06

**Authors:** Thorold W. Theunissen, Marc Friedli, Yupeng He, Evarist Planet, Ryan C. O’Neil, Styliani Markoulaki, Julien Pontis, Haoyi Wang, Alexandra Iouranova, Michaël Imbeault, Julien Duc, Malkiel A. Cohen, Katherine J. Wert, Rosa Castanon, Zhuzhu Zhang, Yanmei Huang, Joseph R. Nery, Jesse Drotar, Tenzin Lungjangwa, Didier Trono, Joseph R. Ecker, Rudolf Jaenisch

**Affiliations:** 1 Whitehead Institute for Biomedical Research, Cambridge, MA 02142, USA; 2 School of Life Sciences, Ecole Polytechnique Fédérale de Lausanne (EPFL), 1015 Lausanne, Switzerland; 3 Genomic Analysis Laboratory and Howard Hughes Medical Institute, Salk Institute for Biological Studies, La Jolla, CA 92037, USA; 4 Department of Biology, Massachusetts Institute of Technology, Cambridge, MA 02142, USA; 5 Bioinformatics Program, University of California, San Diego, La Jolla, CA 92093, USA

**Keywords:** pluripotency, embryonic stem cells, transposable elements, DNA methylation, imprinting, X chromosome reactivation, mouse-human chimeras

## Abstract

Recent studies have aimed to convert cultured human pluripotent cells to a naive state, but it remains unclear to what extent the resulting cells recapitulate in vivo naive pluripotency. Here we propose a set of molecular criteria for evaluating the naive human pluripotent state by comparing it to the human embryo. We show that transcription of transposable elements provides a sensitive measure of the concordance between pluripotent stem cells and early human development. We also show that induction of the naive state is accompanied by genome-wide DNA hypomethylation, which is reversible except at imprinted genes, and that the X chromosome status resembles that of the human preimplantation embryo. However, we did not see efficient incorporation of naive human cells into mouse embryos. Overall, the different naive conditions we tested showed varied relationships to human embryonic states based on molecular criteria, providing a backdrop for future analysis of naive human pluripotency.

## Highlights

• Naive human ESCs share a unique transposon signature with cleavage-stage embryos
• Global DNA demethylation in naive human ESCs is reversible except at imprinted loci
• The X chromosome status of naive human ESCs resembles the preimplantation embryo
• Naive human ESCs incorporate into the mouse morula or blastocyst very inefficiently

## Graphical Abstract

**Figure:**
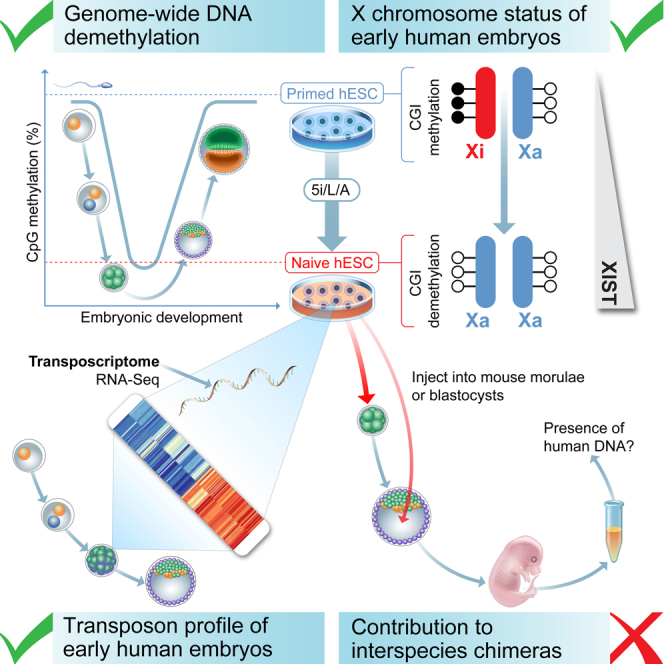

## Teaser

Theunissen et al. use molecular criteria based on transposon expression, DNA methylation, and X chromosome status to compare naive human pluripotent cells to human preimplantation embryos. Current approaches yield cells that most closely resemble the morula/early blastocyst stage.

## Introduction

Pluripotent stem cells from mouse and human have distinct morphologies, signaling requirements and epigenetic configurations. It has been proposed that mouse embryonic stem cells (ESCs) and induced pluripotent stem cells (iPSCs) represent a naive state of pluripotency corresponding to the inner cell mass (ICM), whereas human ESCs/iPSCs correspond to a more advanced, or “primed,” state of pluripotency found in the postimplantation epiblast (Nichols and Smith, 2009). A number of protocols have been described for inducing naive features in human ESCs, mostly testing candidates (Chan et al., 2013, Gafni et al., 2013, Takashima et al., 2014, Ware et al., 2014). We took a systematic approach by screening a kinase inhibitor library for the ability to maintain activity of the distal enhancer of *OCT4* (Theunissen et al., 2014). Through iterative screening, we identified a combination of five kinase inhibitors that, together with LIF and activin A (5i/L/A), enabled the conversion of pre-existing human ESCs to the naive state in the absence of transgenes. An independent analysis concluded that naive human cells generated with this method or in titrated 2i/L medium supplemented with a protein kinase C (PKC) inhibitor (Takashima et al., 2014) displayed the closest transcriptional similarity to both the human blastocyst and mouse ESCs in 2i/L (Huang et al., 2014).

It has been challenging to define the naive state of pluripotency in humans, particularly in view of the expanding number of protocols for deriving putative naive human cells (De Los Angeles et al., 2015, Wu and Izpisua Belmonte, 2015). While robust standards such as chimera formation can be used to definitively define naive pluripotency in mouse ESCs, such assays are not available in the human system, necessitating the establishment of alternative criteria. We and others previously assessed naive human cells according to features of naive pluripotency observed in mouse, such as *OCT4* distal enhancer activity, expression of naive-specific transcripts, and reduced bivalent domains (Chan et al., 2013, Gafni et al., 2013, Takashima et al., 2014, Theunissen et al., 2014, Ware et al., 2014). However, a comprehensive examination of the extent to which naive cells resemble early human embryos has not been described so far. Here we propose rigorous criteria for evaluating naive human pluripotency based on emerging insights into human preimplantation development (Guo et al., 2014, Okamoto et al., 2011, Petropoulos et al., 2016, Yan et al., 2013). A priori, the expectation based on parallels with naive mouse ESCs would be that naive human ESCs would be most closely related to the ICM of the blastocyst. We show using a range of molecular assays that naive human cells in 5i/L/A and other conditions acquire key features of corresponding pluripotent cells in vivo but fail to recapitulate the embryonic context entirely. Our results present a framework for future analysis and improvement of naive culture conditions.

## Results

### Naive Human ESCs Display a Transposon Transcription Signature of Cleavage-Stage Embryos

Transposable elements (TEs) are mobile genetic entities that constitute over half the human genome, and whose sequential expression during embryonic development is tightly regulated by species-specific *trans*-acting factors (Friedli and Trono, 2015). We recently generated an updated census of the transposcriptome, or sum of TE-derived transcripts in a cell, which provides a high-density barcode that can be used to characterize cellular identity. Here we explored whether TE expression profiling can be used to distinguish naive and primed human ESCs, and measure the correspondence of pluripotent stem cells to distinct stages of human embryogenesis.

**Figure 1:**
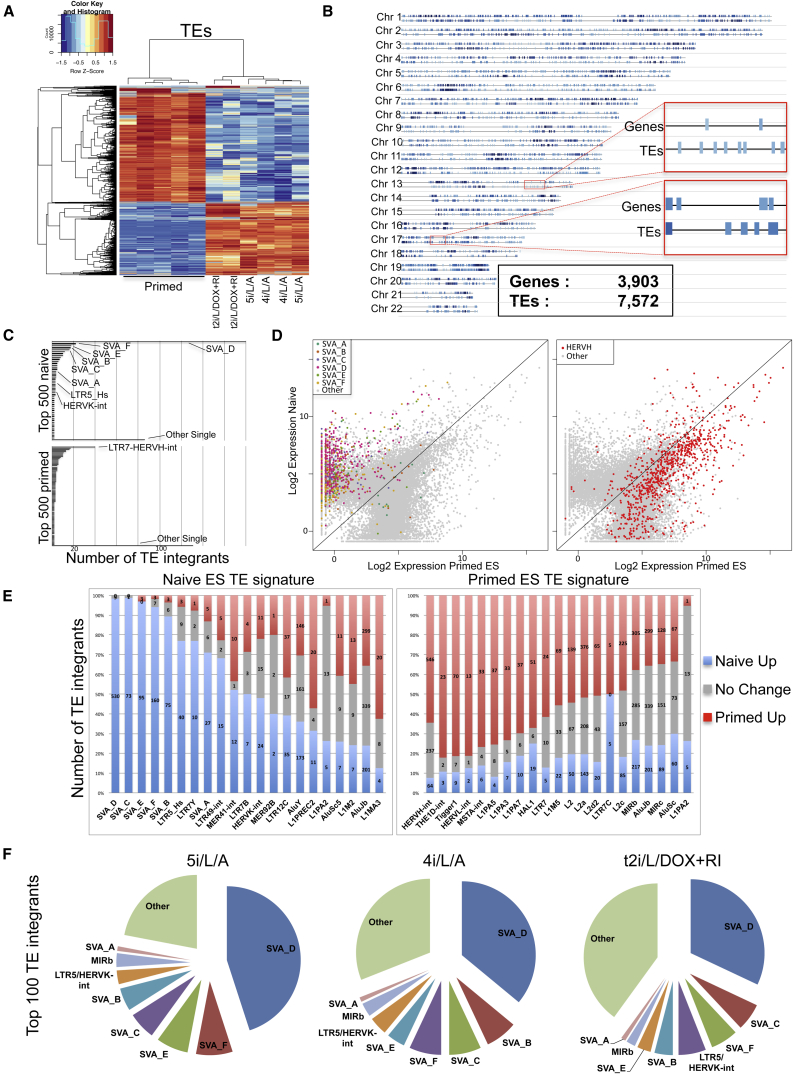
TEs Accurately Discriminate Naive and Primed Human ESCs (A) Heatmap of RNA-seq expression data from primed human ESCs and naive human ESCs derived in 5i/L/A-, 4i/L/A-, and transgene (KLF2/NANOG)-dependent naive cells maintained in t2i/L/DOX+RI. Data shown include 10,000 TEs with the highest SD between the samples. (B) Naive/primed differentially expressed TEs (bottom) or genes (top) are depicted along human chromosomes. A cutoff fold change of 10× (p < 0.05) between naive and primed states was used to visualize the distribution of the most significantly differentially expressed TEs and genes. Note that TEs provide a much denser coverage of the genome. (C) The top 500 TE integrants with the highest fold change between primed and naive ESCs are heavily enriched for HERVH-int-LTR7 (primed) or SVA and LTR5_Hs integrants (naive). (D) Dot plot of all expressed TE integrants in naive versus primed ESCs. SVA-D, SVA-F, SVA-E, SVA-C, and SVA-B integrants are broadly expressed in naive ESCs (left), while SVA-A integrants are more evenly distributed. Similarly, HERVH-ints were by and large overexpressed in primed ESCs (right) (E) TE signatures of naive or primed ESCs. The most heavily primed or naive-biased TE families are represented as columns split into three segments: naive-overexpressed integrants (2-fold cutoff, p adj. < 0.05), primed-overexpressed integrants, and no change (integrants with expression ratio not contained in the above cutoff). (F) Pie charts comparing the top 100 TEs with highest fold change for different naive media (5i/L/A, 4i/L/A, and t2i/L/DOX+RI) compared to primed ESCs. The overall distribution is similar, with SVA-D representing about one-third of the top 100 TEs. See also Figures S1 and S2.

TE-derived transcripts were analyzed using an improved RNA sequencing (RNA-seq) methodology in conventional (primed) human ESCs and three conditions for naive human pluripotency: (1) ectopic expression of doxycycline (DOX)-inducible KLF2 and NANOG transgenes (Takashima et al., 2014, Theunissen et al., 2014); (2) our previously described 5i/L/A conditions (Theunissen et al., 2014); and (3) a modified version of 5i/L/A in which the GSK3 inhibitor is removed (4i/L/A), which confers enhanced proliferation but results in a flatter colony morphology (Figures S1A–S1C, available online). The top 10,000 TEs with largest SD perfectly separated naive and primed cells (Figure 1A). Taking a 2-fold cutoff and p < 0.05, 16,311 loci (Table S1) were differentially expressed between naive and primed cells, with a slight imbalance (38.8% versus 61.2%) in favor of the latter. Differentially expressed TEs provided much denser coverage of human chromosomes than genes, as expected from their relative abundance in the genome (>four million TE integrants versus ∼25,000 genes), producing a high-density barcode for the state of human ESCs (Figure 1B).

We asked which TEs were overexpressed in naive cells and found that members of the SINE-VNTR-*Alu* (SVA) family of TEs, in particular the SVA-D subgroup, were transcribed almost exclusively in the naive state. Of the top ten integrants with the highest naive-to-primed difference, four were SVA-Ds (Table S2), and of the top 500, 258 were SVAs, with a strong predominance of SVA-Ds (181) (Figures 1C and 1D). Of 539 SVA-Ds that produced RNA-seq reads above detection threshold, 530 were differentially expressed (98.3%), all of them at higher levels in the naive state (Figure 1E). SVAs are an evolutionarily young (hominid-specific) class of retroelements, and active retrotransposition of some SVA integrants has been reported in the human genome (Hancks and Kazazian, 2010). We also found the HERVK-associated LTR, LTR5-Hs, to be more readily transcribed in naive cells (52 loci expressed, 82.7% differentially, 93.0% of them more strongly in naive cells).

Surprisingly, none of the top 100 naive-expressed TEs belonged to the LTR7-HERVH family (only two LTR7-HERVHs and two unmerged LTR7s in the top 500), contrary to the recent suggestion that this endogenous retrovirus and its promoters are specific to naive-like cells (Wang et al., 2014) (Figure S2A). Instead, LTR7-HERVH integrants were collectively more expressed in primed cells, with 40 of them among the top 500 TEs of this category (Figures 1C and 1D). Furthermore, of 847 HERVH-int elements for which transcription was detected in at least one of our ESC samples, 610 (72.0%) were differentially expressed, with 546 (89.5%) higher in primed cells (Figure 1E). Globally, the transcription of SVAs (SVA-A to a lesser extent) and of HERVH-int was thus highly polarized, with a majority of integrants from these two TE subfamilies displaying preferential expression in naive and primed states, respectively.

**Figure 2:**
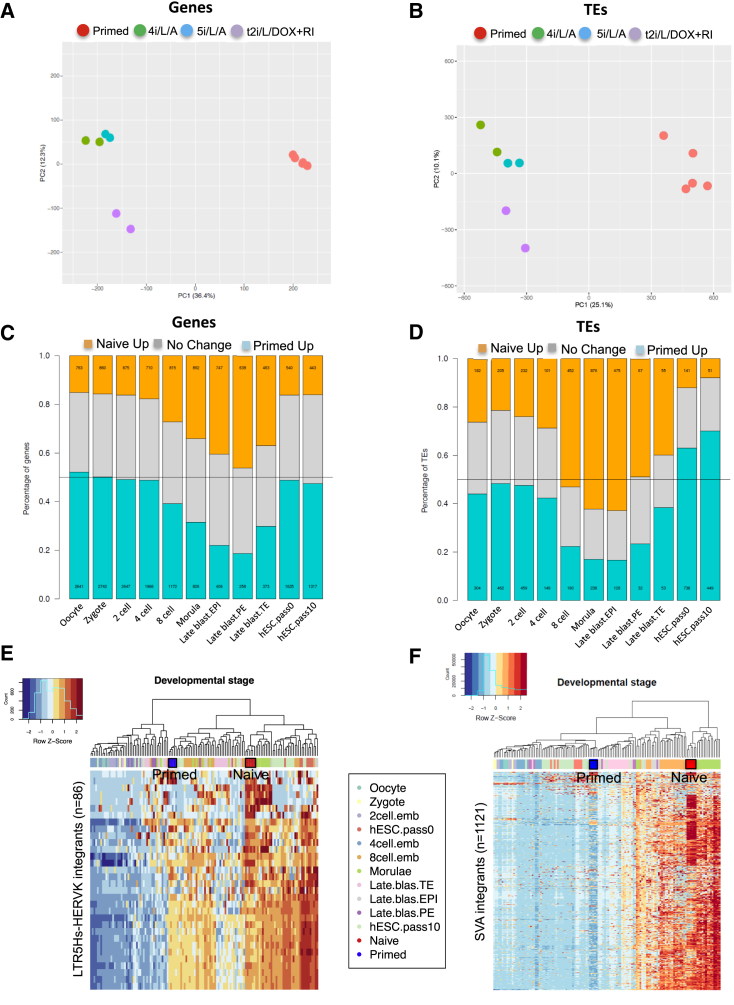
Naive Human ESCs Have a Transposon Transcription Signature of the Human Preimplantation Embryo (A and B) Principal component analysis (PCA) of primed or naive human ESCs derived in 5i/L/A-, 4i/L/A-, and transgene (KLF2/NANOG)-dependent naive cells maintained in t2i/L/DOX+RI based on the differential expression of genes (A) or transposable elements (B). (C and D) Correspondence between gene expression (C) or TE expression (D) in naive/primed ESCs and single-cell human embryonic stages (Yan et al., 2013). For every stage of human embryonic development, a statistical test was performed to find the genes (or TEs) that have a different expression level compared to the other stages. The proportions of developmental stage-specific genes (or TEs) that are upregulated (p < 0.05, 2-fold change) in naive or primed cells are indicated in orange and blue, respectively, while genes (or TEs) that did not change expression are indicated in gray. The naive samples include all three conditions of naive cells that we examined in the PCA analyses (i.e., 5i/L/A, 4i/L/A, and t2i/L/DOX+RI). See Supplemental Experimental Procedures for details of the analysis. (E and F) Heatmaps of RNA-seq data from naive/primed ESCs and from single-cell human embryonic stages (Yan et al., 2013). Clustering is performed with all LTR5_Hs/HERVK elements with expression data (n = 86) (E) or all SVA elements with expression data (n = 1121) (F). The naive samples shown in red include all three conditions of naive cells that we examined in the PCA analyses (i.e., 5i/L/A, 4i/L/A, and t2i/L/DOX+RI). See also Figures S1–S3.

The three conditions for naive human cells that we examined induced comparable upregulation of SVAs (mainly SVA-D) and LTR5_Hs integrants (Figure 1F). The transposcriptomes of naive cells in 5i/L/A and 4i/L/A were especially similar (Figure 1A), reflecting the close alignment of their global gene expression profiles (Figure 2A). Transgene-dependent naive cells were enriched in SVAs and LTR5_Hs expression (Figure 1F), but formed a discrete cluster in terms of TE expression (Figure 1A) or gene expression (Figure 2A). Principal component analysis (PCA) based on differentially expressed TEs uncovered greater variability between biological replicates than gene expression-based PCA (Figures 2A and 2B), highlighting the ability of TEs to uncover subtle differences between cell states.

Given the enhanced sensitivity of TE profiling, we hypothesized that the transposcriptome might provide a precise measure of the degree to which naive and primed ESCs resemble pluripotent cells in vivo. We therefore matched TE-derived RNA-seq data from naive and primed human ESCs with previous single-cell analyses of early human embryos containing an appropriate read length for transposcriptome analysis (the source loci of many TE-derived transcripts cannot be properly mapped if reads are below 100 bp) (Yan et al., 2013). Naive cells displayed the most significant overlap with the human morula and epiblast stages: 62% (n = 876) of morula-enriched TEs and 63% (n = 475) of epiblast-enriched TEs were upregulated in naive compared to primed human ESCs (p < 0.05, 2-fold change) (Figures 2D and S1E). This suggests that 5i/L/A and 4i/L/A induce a pluripotent state that corresponds most closely to a late morula or early blastocyst identity. Based on gene expression, naive cells were most closely aligned with the late blastocyst (Figures 2C and S1D), but TE profiling revealed a greater contrast between naive and primed states (Figures 2D and S1E). Naive human cells generated with inducible KLF2 and NANOG transgenes in t2i/L+PKCi (Takashima et al., 2014) presented largely similar gene and TE expression profiles, but upregulation of morula-specific TEs was more pronounced in 5i/L/A and 4i/L/A (Figure S2B). In contrast, the NHSM protocol (Gafni et al., 2013) did not induce blastocyst-specific genes or TEs to a significant extent in our hands (Figure S2C), though the original dataset (Gafni et al., 2013) cannot be analyzed in a comparable way. Primed cells shared gene and TE expression with early-passage human ESCs (post-natal day 0 [P0] and P10) and very early (one- to four-cell-stage) human cleavage embryos. The overlap in gene expression between primed cells and one- to four-cell-stage embryos was noted before and attributed to upregulation of mitotic regulators that are expressed before embryonic genome activation occurs (Huang et al., 2014). Thus, we conclude that the transposcriptome provides a robust sensor of the correspondence between pluripotent states in vitro and the human preimplantation embryo.

Epiblast cells from late-stage mouse blastocysts have been identified as the closest in vivo counterpart of naive mouse ESCs based on their gene expression profile and capacity as a source for ESC derivation (Boroviak et al., 2014). Hence, the finding that naive human cells display comparable induction of morula and epiblast-associated TEs was unexpected. The overlap between the transposcriptomes of naive human cells and morula-stage embryos was driven largely by the pronounced upregulation of SVA and LTR5_Hs integrants: unsupervised clustering based on the expression of these two TE families revealed a strong convergence between naive cells and morulae, whereas primed cells clustered most closely with previously analyzed human ESCs at passage 10 (Figures 2E and 2F). This transposon transcription signature points to a slightly earlier developmental stage as being the equivalent of naive human cells than would necessarily be predicted. However, as a result it is possible that these naive human cells might also be able to differentiate into the extra-embryonic lineages. Indeed, we observed significant upregulation in the naive state of 8 out of 16 transcription factors reported to be overexpressed in trophectoderm and placenta compared to conventional human ESCs (Bai et al., 2012) (Figure S3). These observations suggest that naive human cells could provide a convenient model system to study the initiation of trophectoderm differentiation.

### Control of Transposon Transcription in Naive and Primed Human ESCs

**Figure 3:**
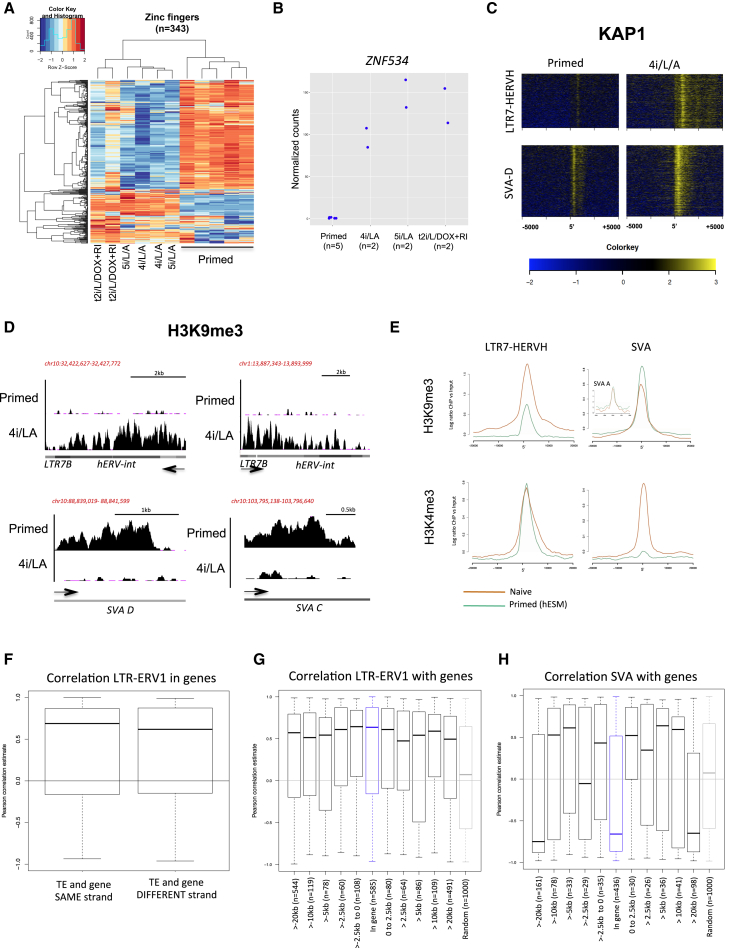
Control of Transposon Transcription in Naive and Primed Human ESCs (A) Heatmap of RNA-seq data from naive/primed ESCs depicting all KRAB-ZFP genes with expression data (n = 345). (B) RNA-seq quantification of *ZNF534* in naive and primed ESCs. (C) Density heatmaps of KAP1 for SVA or LTR7-HERVH. The average profile was determined at ± 20 kb around the 5′ of all elements for each family. This average is calculated from log ratio of ChIP reads over input DNA. KAP1 was chromatin immunoprecipitated in naive (WIBR3 in 4i/L/A) or primed ESC conditions (WIBR3 in hESM). For ChIP, naive cells were cultured in 4i/L/A, as these conditions enabled more rapid large-scale expansion. (D) Examples of H3K9me3 ChIP signals in naive and primed cells over primed or naive-specific TEs. Screenshot of H3K9me3 ChIP-seq in WIBR3 cells in primed media (hESM) versus naive media (4i/L/A). (E) H3K9me3 and H3K4me3 ChIP-seq average profiles for LTR7-HERVH (all LTR7 associated 5′ to an HERVH-int) and all SVA elements. The average profile was determined at ± 20 kb around the 5′ of all those elements. This average corresponds to log ratio of number of reads in chromatin immunoprecipitated over input DNA. H3K9me3 and H3K4me3 were produced from primed ESCs, H3K4me3 in 5i/L/A and JNK inhibitor (6i/L/A) (Theunissen et al., 2014), and H3K9me3 in 4i/L/A (this study). (F) Boxplot showing TE-gene pair expression correlation for TEs located within genes in either sense or antisense orientations. The correlation is not strand specific. Line in the center of box indicates the median. Whiskers indicate maximum or minimum values within 1.5 interquartile range to upper (or lower) quartile. (G and H) Boxplots displaying correlation between the expression of LTR-ERV1 integrants (n = 2,324), including HERVH-int (G) or SVA integrants (n = 1003) (H) with expression of closest genes. TEs were categorized according to distance to genes with the following bins: in gene (introns), <2.5, 2.5–5, 5–10, 10–20 kb, and random. Correlation is greatest near genes and tails off as distance increases. Line in the center of box indicates the median. Whiskers indicate maximum or minimum values within 1.5 interquartile range to upper (or lower) quartile.

The group-like behavior of SVA-D and LTR7/HERVH integrants suggested that these TEs might be collectively subjected to the influence of *trans*-regulators capable of controlling multiple members of a same family. The KRAB-ZFP/KAP1 system is central to the repression of a broad range of TEs during early embryogenesis (Matsui et al., 2010, Rowe et al., 2010, Turelli et al., 2014), and we recently determined that a vast majority of human KRAB-ZFPs have TEs as their preferential genomic targets (unpublished data). Correspondingly, most KRAB-ZFP genes displayed highly state-specific modes of expression in human ESCs, being markedly more expressed either in naive or in primed cells (Figure 3A). Levels of *ZNF534*, a KRAB-ZFP we identified as responsible for recognizing HERVH (unpublished data), were significantly higher in naive than primed ESCs (Figure 3B).

Among the top 100 TEs preferentially expressed in primed ESCs, twelve were enriched for KAP1 in naive cells and none in primed cells, as determined by chromatin immunoprecipitation followed by deep sequencing (ChIP-seq). As expected from the transposcriptome data, HERVH integrants were much less frequently KAP1 bound in primed than naive ESCs (Figure 3C). Interestingly, KAP1 binding extended further 3′ into LTR7-HERVH-int merged elements in naive ESCs, suggesting an additional specific KAP1 binding site within HERVH-int in this context (Figure 3C). Consistent with their repression, we detected H3K9me3 enrichment at many HERVH elements in naïve, but not primed, ESCs (Figures 3D and 3E). No overall difference in H3K4me3 was observed at these elements, consistent with a poised state in naive ESCs (Figure 3E). As expected from their naive-restricted expression, SVA elements exhibited reduced H3K9me3 and increased H3K4me3 enrichment in naive ESCs (Figures 3D and 3E), despite slightly increased KAP1 deposition in this setting (Figure 3C). This strongly suggests that on these elements, as previously observed at other loci (Iyengar and Farnham, 2011, Singh et al., 2015), KAP1 does not act as a co-repressor. However, members of the SVA-A subfamily, the only one not to display strong naive-biased expression, bore H3K9me3 in both primed and naive ESCs (Figure 3E). Thus, LTR7-HERVH and SVAs show dichotomous transcriptional and epigenetic marks specific to primed and naive states, respectively.

TEs can influence transcription through a variety of mechanisms (Friedli and Trono, 2015). Supportive of promoter or proximal enhancer effects, we found a significant correlation between expression of genes and their closest TEs. This correlation was independent of the respective orientations of the two components of a TE-gene pair (Figure 3F) and tailed off as their distance increased (Figures 3G and 3H). Interestingly, the effect on gene expression appeared more local for LTR-containing TEs with expression correlation for TEs located within the gene boundaries or in near vicinity, consistent with promoter activity (Figure 3G). SVAs, on the other hand, did not influence gene expression when located within genes and appeared to act at longer distances from genes, suggesting enhancer effects (Figure 3H).

### Naive Induction Is Accompanied by a Genome-Wide Depletion in DNA Methylation that Is Reversible upon Differentiation except at Imprinted DMRs

**Figure 4:**
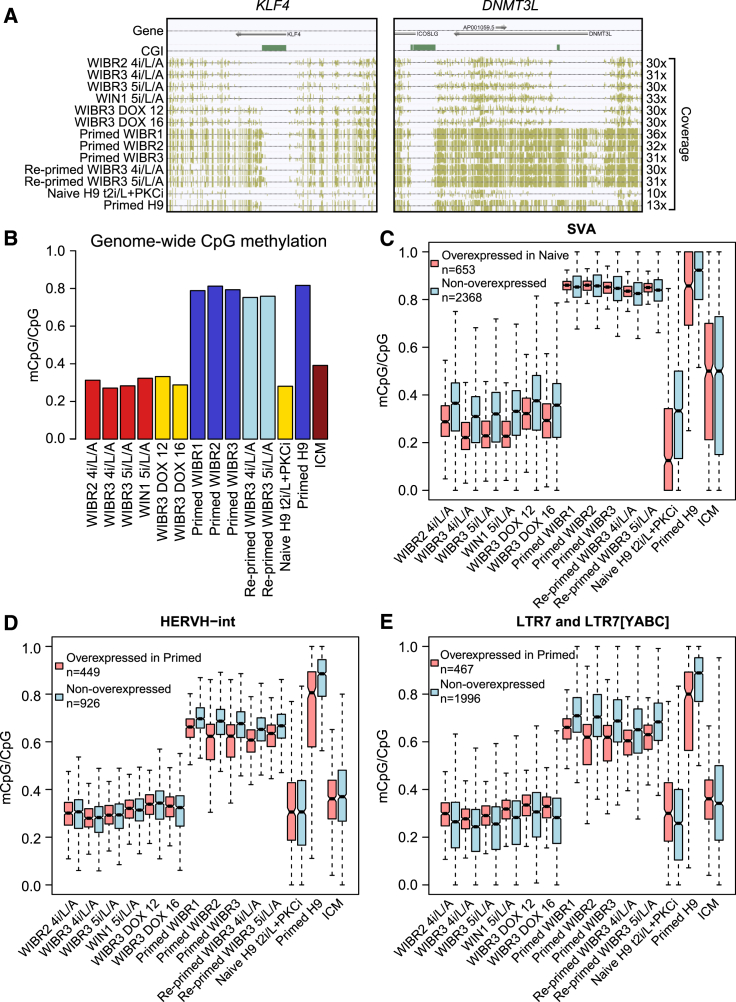
Base-Resolution DNA Methylomes of Naive and Primed Human ESCs (A) Browser screenshots of two representative regions showing CpG hypomethylation in naive human ESCs compared with primed ESCs. Gene annotations are displayed in the top track. The second track shows the locations of CGIs. CpG methylation is shown as gold tick marks and the heights are proportional to the methylation levels. For each track, ticks projecting upward and downward indicate the methylation on the Watson and Crick strand, respectively. Mean coverage on cytosines in each sample is shown on the right. (B) Genome-wide CpG methylation level of all samples described in (A) and ICM data from Guo et al. (2014). (C) Boxplot displaying the methylation levels of SVA copies that were overexpressed in naive versus primed ESCs (red) as well as the methylation state of the non-overexpressed copies (blue). “Overexpressed” defines elements with significantly increased expression in naive ESCs versus primed ESCs (1% FDR; see Supplemental Experimental Procedures for details). Bold line in the center of box indicates the median and notch around it shows the confidence interval (calculated by the “boxplot” function in R). Whiskers indicate maximum or minimum values within 1.5 interquartile range to upper (or lower) quartile. (D and E) Boxplot displaying the methylation levels of (D) HERVH-int and (E) LTR7 and LTR7[YABC] copies that are overexpressed in primed versus naive human ESCs as well as other non-overexpressed copies from the same family. See also Figure S4.

Human preimplantation development is marked by a global reduction in DNA methylation (Guo et al., 2014). We generated high-coverage base-resolution methylomes (>30× coverage) of naive and primed human ESCs using MethylC-seq (Lister et al., 2009, Urich et al., 2015) (Table S3). All conditions examined for naive human cells (5i/L/A, 4i/L/A, and DOX-inducible expression of KLF2 and NANOG transgenes) were characterized by global hypomethylation in both CpG (26.9%–33.2%) and non-CpG contexts (0.19%–0.29%) and compared to their primed and re-primed counterparts (CpG, 75.2%–81.0%; non-CpG, 0.32%–0.60%) (Figures 4A, 4B, S4A, and S4B; Table S3). Similarly, widespread hypomethylation was observed in human cleavage-stage embryos (Guo et al., 2014) and naive human ESCs maintained in t2i/L+PKCi (Takashima et al., 2014) (Figures 4B and S4A; Table S3). Such CpG and non-CpG demethylation in naive cells was not restricted to specific genomic features but occurred at CpG islands (CGIs), exons, introns, promoters, and enhancers (Figures S4C and S4D).

CpG methylation is a major mechanism controlling TE expression during human embryonic development (Guo et al., 2014). We therefore investigated the correlation between CpG methylation and TE expression in naive and primed human ESCs. Along with genome-wide demethylation, TEs in naive cells were hypomethylated relative to primed cells (Figures S4C and S4D). Consequently, directly comparing the methylation levels of TEs in naive and primed cells was uninformative. Instead, we compared the methylation levels of overexpressed TEs and non-overexpressed copies from the same repeat family (as control). In naive cells, we focused on the most overexpressed family, SVA. Notwithstanding the overall low methylation in naive ESCs, overexpressed TE integrants tended to be further hypomethylated compared to non-overexpressed copies in naive cells (Figure 4C; p < 0.05, Mann-Whitney test). Likewise, for the primed cells we focused on the most overexpressed groups, HERVH-int and LTR7 sub families (LTR7, LTR7Y, LTR7B, and LTR7C), and found these to be hypomethylated compared to their non-primed-overexpressed counterparts (Figures 4D and 4E; p < 0.05, Mann-Whitney test).

**Figure 5:**
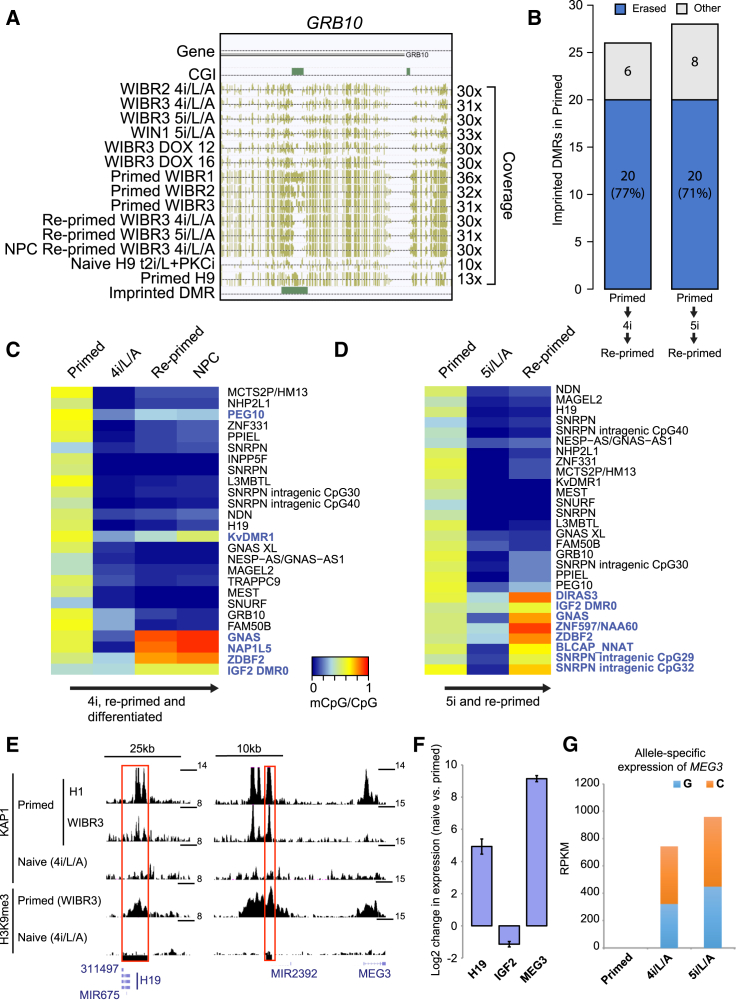
Global DNA Demethylation in Naive Human ESCs Is Reversible except at Imprinted DMRs (A) Browser screenshot of one representative imprinted DMR where methylation is erased in naive human ESCs and is not restored to primed methylation state in re-primed cells or differentiated cells (NPC re-primed WIBR3 4i/L/A). (B) The number and fraction of imprinted DMRs in primed WIBR3 that were erased (blue) in both naive and re-primed cells. Gray proportion marks the imprinted DMRs that did not lose DNA methylation in either naive or re-primed ESCs. (C and D) Heatmaps displaying the CpG methylation levels of imprinted DMRs in WIBR3-primed ESCs before and after conversion in 4i/L/A (C) or WIBR3 (AAVS1-GFP targeted subclone) ESCs before and after conversion in 5i/L/A (D) together with re-primed derivatives. Names of nearby genes for each region are shown on the right. Imprinted DMRs highlighted in blue retained some CpG methylation in either naive or re-primed cells. (E) KAP1 and H3K9me3 ChIP-seq signals at imprinted DMRs near *H19* and *MEG3* genes in naive and primed ESCs. KAP1 was chromatin immunoprecipitated in naive (WIBR3 4i/LA) or two primed ES conditions (primed WIBR3 from this study and H1 in mTesR1; Turelli et al., 2014). H3K9me3 was chromatin immunoprecipitated in primed WIBR3 ESCs or WIBR3 4i/L/A. (F) RNA-seq quantification of *H19*, *IGF*, and *MEG3* expression in WIBR2-primed ESCs and naive ESCs in 5i/L/A or 4i/L/A. Error bars indicate ± 1 SD. (G) SNP analysis shows that *MEG3* is expressed from both alleles in WIBR2 naive ESCs in 5i/L/A or 4i/L/A. See also Figure S5.

Differentially methylated regions (DMRs) within imprinted regions are protected from both active and passive demethylation during preimplantation development to safeguard the transmission of parent-of-origin-specific epigenetic marks (Lee et al., 2014). We examined whether imprinted DMRs in human ESCs (Court et al., 2014) were resistant to methylation erasure upon conversion to the naive state. While imprinted DMRs had an intermediate level of CpG methylation (0.3–0.7) in primed cells, methylation in these regions was dramatically reduced in naive lines, suggesting demethylation of both alleles (Figures 5A and 5B; Table S4). In total, 77% of imprinted DMRs were erased upon 4i/L/A conversion and 71% upon 5i/L/A conversion, where “erased” is defined as regions where the intermediate methylation in starting primed cells becomes hypomethylated (mCpG/CpG < 0.3) in both naive and re-primed cells (Figure 5B; see Supplemental Experimental Procedures). Re-analysis of methylation data from naive human cells in t2i/L+PKCi (Takashima et al., 2014) revealed a similar reduction in CpG methylation at imprinted DMRs: 77% of imprinted DMRs in primed H9 were erased in naive H9 (Table S4). While methylation levels were restored upon re-priming at flanking regions, imprinted DMR methylation remained low regardless of whether the naive state was induced in 5i/L/A or 4i/L/A (Figures 5A–5D and S5A–S5F; Supplemental Experimental Procedures). SNPs revealed the loss of allele-specific methylation at two CpGs in an imprinted DMR near the *SNRPN* gene (Figure S5G; Supplemental Experimental Procedures). We also observed transcriptional amplification of some imprinted genes in naive cells following the combined loss of DNA methylation, KAP1 binding, and H3K9me3 deposition (Figures 5E and 5F; Table S4). The presence of a SNP in a transcribed region of *MEG3* showed that this imprinted gene was expressed from both alleles in naive cells (Figure 5G). The loss of imprinted DMR methylation in naive human cells indicates that they do not entirely recapitulate the in vivo naive pluripotent state. However, it is possible that maintaining naive cells in culture causes extensive demethylation events and erasure of CpG methylation in imprinted DMRs, which may not occur during the few cell divisions in cleavage embryos.

### X Chromosome Status of Female Naive ESCs Resembles the Human Preimplantation Embryo

In the human preimplantation embryo, both X chromosomes are actively transcribed despite expression of *XIST* (Okamoto et al., 2011, Petropoulos et al., 2016). We previously showed that conversion of female primed lines to the naive state results in activation of *XIST*, but reduced expression of X-linked genes suggested the presence of an inactive X chromosome (Theunissen et al., 2014). However, a recent study reported that X chromosome genes maintain biallelic expression while dosage compensation proceeds during human preimplantation development (Petropoulos et al., 2016). Here we re-examined the X chromosome status of naive human cells using methylation data and allele-specific expression methods.

**Figure 6:**
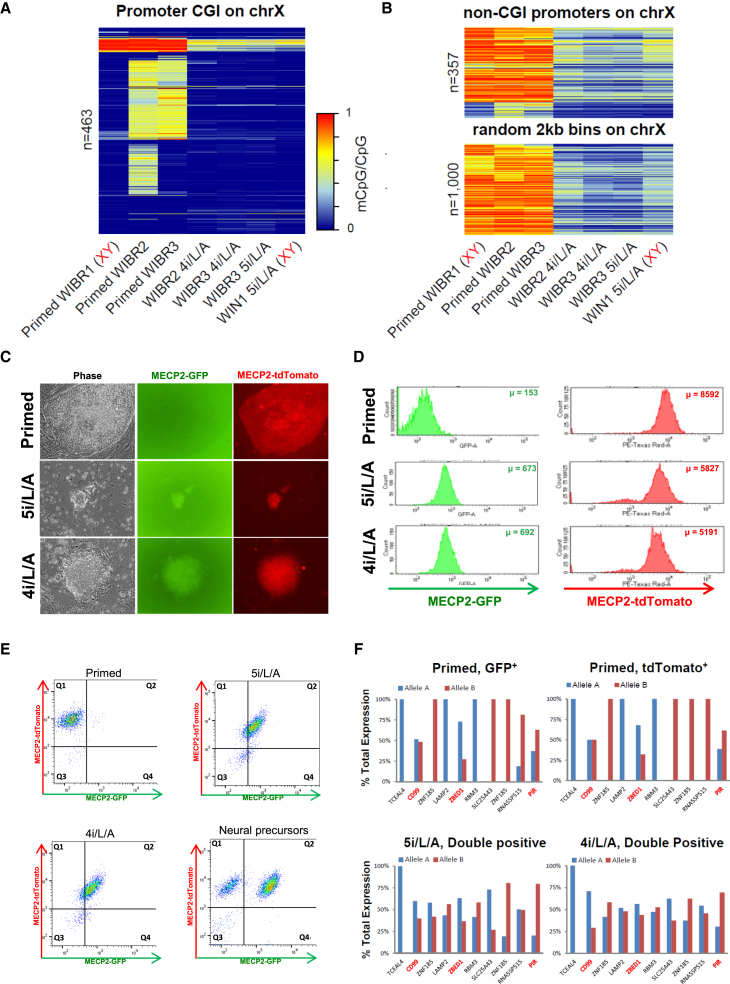
X Chromosome Status of Female Naive Human ESCs (A) CpG methylation of X-linked promoter CpG islands (CGIs) (n = 463) in naive and primed human ESCs. Male control lines are indicated in red. (B) CpG methylation levels of non-CGI promoter regions (n = 357) or randomly chosen 2 kb bins (n = 1,000) along the X chromosome in naive and primed human ESCs. The 2 kb bins do not overlap any CGIs or non-CGI promoters. Male control lines are indicated in red. (C) Phase and fluorescence images of MECP2^GFP-OFF/Tom-ON^ primed cells in hESM, and after conversion to the naive state in 5i/L/A and 4i/L/A. (D) Histograms reporting the levels of MECP2-GFP and MECP-tdTomato in MECP2^GFP-OFF/Tom-ON^-primed cells in hESM, and after conversion to the naive state in 5i/L/A and 4i/L/A. Note that the maximum intensity of MECP2-GFP is significantly weaker than MECP2-tdTomato. (E) Flow cytometric analysis showing the distribution of MECP2-GFP and MECP-tdTomato in MECP2^GFP-OFF/Tom-ON^-primed cells in hESM, after conversion to the naive state in 5i/L/A and 4i/L/A, and upon differentiation into neural precursors via a re-primed intermediate step. (F) Allele-specific gene expression analysis using SNPs located within transcribed regions of X-linked genes in single-color MECP2^GFP-ON/Tom-OFF^- or MECP2^GFP-OFF/Tom-ON^-primed cells in hESM (top) and double-color naive cells maintained in 5i/L/A or 4i/L/A for ten passages (bottom). Red asterisks mark genes reported to escape X inactivation (see Supplemental Experimental Procedures). See also Figures S6 and S7.

Genes that undergo X inactivation have been shown to acquire DNA methylation at CGIs in promoter regions (Weber et al., 2007). Substantial fractions of X-linked promoter CGIs showed an intermediate methylation level in female primed lines, consistent with the presence of both an unmethylated (active) and methylated (inactive) allele (Figures 6A and S6A). In contrast, female naive cells displayed uniform hypomethylation at X-linked promoter CGIs, similar to male cells (Figures 6A and S6A). Most non-CGI-containing X-linked promoters were not differentially methylated between male and female primed cells, but instead reflected the genome-wide methylation levels of naive and primed states (Figures 6B, S6B, and S6C). The dramatic reduction in methylation of X-linked promoter CGIs suggested that naive conversion may result in X chromosome reactivation.

To distinguish between monoallelic and biallelic expression of X-linked genes, we targeted both alleles of *MECP2* with GFP or tdTomato sequences fused in frame with exon 3 (Figures S6D and S6E). Fluorescent activity was followed in two independent double-targeted MECP2 reporter clones. The first clone expressed the GFP allele in the primed state but contained a fraction of cells with GFP and tdTomato co-expression. Upon conversion to the naive state, both MECP2 alleles became uniformly expressed (Figures S6F, S6G, and S7A). A second clone that expressed the tdTomato allele in the primed state also displayed activation of both MECP2 alleles upon naive conversion (Figures 6C–6E). Differentiation into neural precursors resulted in the re-appearance of single-color-positive cells (Figure 6E). However, expression was biased toward one of the MECP2 alleles, suggesting that most cells did not undergo random X inactivation upon differentiation.

We used SNPs to quantify allele-specific gene expression in MECP2-reporter cells converted in 5i/L/A and maintained with or without GSK3 inhibition for ten passages. To ensure a direct comparison between naive and primed states, this analysis was restricted to SNP-containing X-linked transcripts with at least ten reads in all samples. Whereas the starting primed cells showed a largely monoallelic expression profile at the interrogated transcripts, the naive cells displayed a shift toward biallelic expression (Figure 6F). As we reported previously (Theunissen et al., 2014), total X-linked gene expression was reduced upon naive conversion when compared to female primed cells (Figure S7B). However, we now realized that X-linked expression also differed substantially between male primed and male naive cells (Figure S7B). To control for the observed variation in transcriptional output, we compared total X-linked expression levels in female naive cells to male naive cells and found that female naive cells had slightly increased X-linked expression (Figure S7C). A recent study described a progressive dampening of expression from both X chromosomes during human preimplantation development, which results in female cells acquiring a total X-linked expression level similar to male cells by the late blastocyst stage (Petropoulos et al., 2016). The observation that dosage compensation is still incomplete in 5i/L/A provides a further indication that this state may correspond most closely to the late-morula or early-blastocyst rather than the late-blastocyst stage, as was suggested from the transposcriptome profile (Figure 2D). In summary, the presence of two active X chromosomes and upregulation of *XIST* (Figure S7D) indicate that naive cells acquire an X chromosome signature close to that observed in early human development (Okamoto et al., 2011, Petropoulos et al., 2016) and suggest that they could be a useful model for studying human X inactivation mechanisms.

### A Sensitive Mitochondrial PCR Assay Shows that Naive Human ESCs Rarely Contribute to Interspecies Chimeras after Morula or Blastocyst Injection

**Figure 7:**
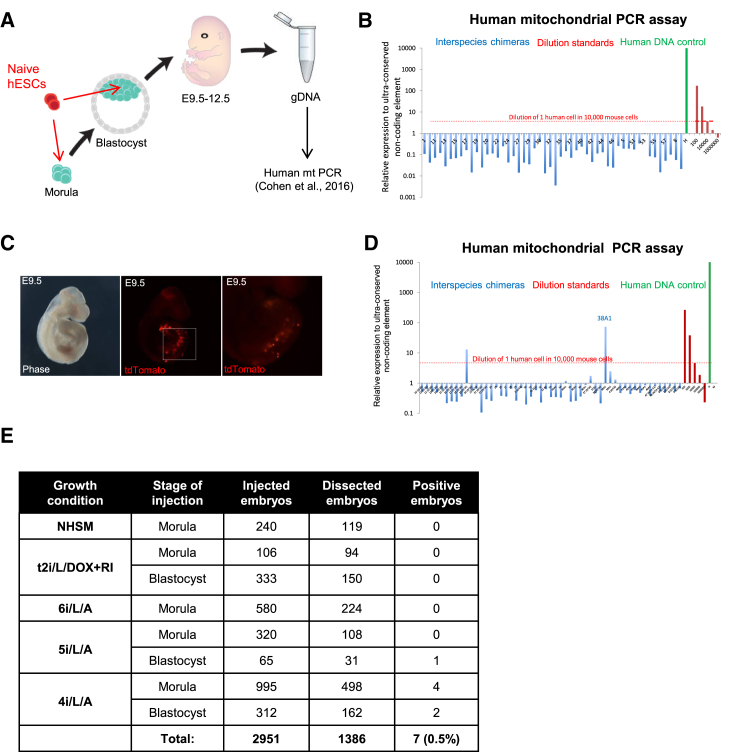
Assessing the Potential of Naive Human Cells to Generate Interspecies Chimeras Using a Sensitive Assay for Human Mitochondrial DNA (A) Schematic overview of strategy to assess the contribution of naive human ESCs to interspecific chimera formation after injection into mouse morula- or blastocyst-stage embryos. (B) Sample qPCR analysis for human mitochondrial DNA following injection of naive human ESCs. This chart shows the lack of human DNA detection after injection of naive human ESCs maintained by inducible overexpression of KLF2 and NANOG transgenes into mouse blastocysts. Embryos were harvested at E10.5. A human DNA control (green bar) and a series of human-mouse cell dilutions (red bars) were run in parallel to estimate the degree of human cell contribution. The dotted line indicates the detection level of human mitochondrial DNA equivalent to a dilution of one human cell in 10,000 mouse cells. (C) Phase and fluorescence images of a mouse embryo at E9.5 with discernable tdTomato signal following injection with tdTomato-labeled naive ESCs in 4i/L/A at the morula stage and transfer to a pseudopregnant female. (D) qPCR analysis for human mitochondrial DNA indicated the presence of human cells in mouse embryos at E9.5–E12.5 following injection of naive human ESCs in 4i/L/A at the morula or blastocyst stages. The presence of human cells in embryo 38A1 was confirmed by visual inspection for the presence of tdTomato (C). (E) Summary of injections of naive human ESCs at the mouse morula or blastocyst stages, numbers of injected and tested embryos, and positive embryos based on a sensitive human mitochondrial PCR assay with a detection threshold of approximately one human cell for every 10,000 mouse cells. Genomic DNA was isolated between E9.5 and E12.5.

Contribution to in vivo development after injection into morula- or blastocyst-stage embryos is the most stringent criterion of naive pluripotent cells in the mouse system. In a previous study, we investigated the potential of 5i/L/A naive human ESCs as well as the naive cells described in Gafni et al. (2013) to contribute to interspecific chimera formation following injection into early mouse embryos, as assessed by the presence of fluorescence in embryos (Theunissen et al., 2014). Because the sensitivity of detecting GFP reporter cells in embryos is low, and because auto-fluorescence may lead to false-positive results, we repeated these experiments using a quantitative and highly sensitive molecular assay based on the detection of an amplified human mitochondrial DNA segment by PCR (Cohen et al., 2016). GFP-labeled naive human ESCs described in Gafni et al. (2013) and tdTomato-labeled naive human ESCs maintained by KLF2 and NANOG transgenes or small molecules were injected at the morula or blastocyst stages. Embryos were isolated between embryonic day 9.5 (E9.5) and E12.5 and inspected under a fluorescence microscope. Dissected embryos were further screened using the PCR assay for human mitochondrial DNA, which has a detection limit of one human cell in 10,000 mouse cells (Cohen et al., 2016). No contribution of human cells was detected after injecting naive cells derived in NHSM (n = 119) (Gafni et al., 2013), 5i/L/A and JNK inhibition (6i/L/A) (n = 224), or DOX-dependent naive cells (n = 244) (Figures 7A, 7B, and 7E). A small number of embryos with discernable human DNA signal were obtained using 5i/L/A (0.7%, n = 139) or 4i/L/A (0.9%, n = 660), indicating the presence of one human cell in 10,000 mouse cells (up to 1 in 2,000 mouse cells in a few embryos; Figures 7C–7E). Thus, though human donor cells were detected in the mouse embryos, the rare incidence of human cell incorporation precluded the examination of their fate. The results summarized in Figure 7E failed to detect a significant difference between the various human donor cells in their low efficiency in colonizing mouse embryos. Moreover, it should be emphasized that the PCR assay can only detect the presence of human DNA but is not suitable to ascertain survival of human cells in the embryo, as the human DNA could be derived from lysed or dead cells.

We conclude that, at least in our hands, the generation of interspecies mouse-human chimeras by injection of naive ESCs into the mouse morula or blastocyst is currently too sporadic for definitive assessment of functional contribution, and we do not propose including it as part of the molecular assessment of naive human pluripotency.

## Discussion

Recent years have seen a surge of interest in capturing human ESCs in a naive state more akin to mouse ESCs. However, evidence has accumulated that mouse and human preimplantation development are highly divergent, and that mouse ESCs by extension do not provide an appropriate standard for naive human cells. Notable differences in early development between the two species include diapause in mouse (Nichols and Smith, 2009), the failure to restrict hypoblast formation upon FGF/MEK inhibition in humans (Roode et al., 2012), distinct expression patterns of pluripotency and lineage markers (Blakeley et al., 2015, Yan et al., 2013), and the activation of *XIST* in early human embryos (Okamoto et al., 2011, Petropoulos et al., 2016). Here we propose that putative naive human cells should be examined according to their resemblance to human pluripotent cells in vivo. We established stringent criteria for evaluating the naive state of human pluripotency using three comparative molecular parameters for which data from early human embryos are available: (1) the expression profile of transposable elements based on single-cell RNA-seq data (Yan et al., 2013), (2) the DNA methylation landscape of human preimplantation development (Guo et al., 2014), and (3) the X chromosome status of female human embryos (Okamoto et al., 2011, Petropoulos et al., 2016). As an additional criterion, we examined whether the generation of interspecies chimeras by injection of naive human cells into early mouse embryos would provide a functional assay for the naive human pluripotent state.

First, we demonstrate that a comprehensive examination of the transposcriptome offers a more sensitive measure of the correspondence between pluripotent stem cells and the early human embryo than gene expression profiling. As expected from their relative abundance in the genome, differentially expressed TEs between naive and primed human ESCs provided much denser coverage of human chromosomes than differentially expressed genes. Comparison with single-cell RNA-seq data from early human embryos (Yan et al., 2013) revealed a close similarity between naive human ESCs in 5i/L/A and both morula- and epiblast-stage embryos. The upregulation of morula-associated TEs, in particular SVA and LTR5_Hs integrants, was unexpected in view of mounting evidence that epiblast cells of the late blastocyst represent the in vivo equivalent of naive mouse ESCs (Boroviak et al., 2014, Boroviak et al., 2015). Our findings suggest that 5i/L/A induces a pluripotent state in human cells that corresponds most closely to a late morula or early blastocyst identity. Contrary to previous reports based on naive-like cells derived in 2i/L conditions (Wang et al., 2014), we find that expression of HERVH/LTR7 integrants is more strongly associated with the primed state. We also explored the mechanisms controlling TE expression in naive and primed cells. In some cases, as for the ZNF534-LTR7/HERVH pair, we could match the downregulation of the KRAB-ZFP with KAP1 depletion, loss of repressive marks, and transcriptional activation at the corresponding TE target. While naive human cells in t2i/L+PKCi (Takashima et al., 2014) had a very similar gene expression profile to 5i/L/A, transposcriptome profiling revealed subtle differences in the expression of developmental stage-specific TE integrants. On the other hand, the NHSM protocol (Gafni et al., 2013) in our hands did not induce activation of blastocyst-associated TEs and appears to induce a distinct state. It will be of interest to explore how specific modifications of the culture environment bring the TE profile of naive human ESCs even closer to that of pluripotent cells in vivo.

Second, we show that a distinctive trademark of naive human ESCs is a genome-wide reduction in DNA methylation to levels equivalent to human eight-cell, morula, and blastocyst embryos. As predicted, expression of the different transposon classes correlated with reduced DNA methylation. DMRs of imprinted genes were differentially methylated in primed ESCs but demethylated in naive ESCs obtained in 5i/L/A or t2i/L+PKCi (Takashima et al., 2014), consistent with global DNA demethylation. However, while global DNA methylation levels were restored when naive cells were converted back to the primed state, imprinted DMRs remained hypomethylated. This is similar to previous results where global demethylation was induced by deletion of Dnmt1 in mouse ESCs, but upon re-expression of Dnmt1 methylation was restored in the bulk genome, but not at imprinted genes (Tucker et al., 1996). Both of these observations are consistent with the strict methylation dependence of the DNA binding of ZFP57, the KRAB-ZFP responsible for tethering the histone and DNA methylation maintenance machinery at imprinting control regions (Quenneville et al., 2011). Once methylation is completely lost, imprinting of the corresponding DMR cannot be restored because the methylation complex can no longer be recruited. While this study was in preparation, Clark and colleagues reported a similar loss of imprinting in naive human ESCs derived in 5i/L/A supplemented with recombinant FGF (Pastor et al., 2016). Loss of imprinting represents a departure from the methylome of pluripotent cells in vivo and limits future applications of naive human ESCs. These results underline the need to identify parameters in the culture environment that protect against extensive demethylation of imprinted DMRs upon long-term maintenance of naive human ESCs.

Third, recent studies have shown that X chromosome dynamics during human preimplantation development are markedly different from mouse, providing a unique blueprint for the naive human pluripotent state. In contrast with early mouse development, human female preimplantation embryos display two active X chromosomes but also express *XIST* (Okamoto et al., 2011, Petropoulos et al., 2016). We found that naive induction in 5i/L/A resulted in X chromosome reactivation, as indicated by reduced DNA methylation at X-linked promoter CGIs, the conversion of single-positive primed MECP2 reporter cells to GFP/tdTomato double-positive naive cells, and biallelic expression of X-linked genes. Unlike other protocols for generating naive-like human cells, 5i/L/A also induced derepression of *XIST*. Our previous conclusion that naive human cells contain an inactive X chromosome was based on a similar level of X-linked gene expression in male primed and female naive cells (Theunissen et al., 2014). However, it has recently been shown that female human embryos undergo progressive dosage compensation from the morula stage onward by downregulation of X-linked genes, resulting in female cells acquiring a total X-linked expression level similar to male cells by the late blastocyst stage (Petropoulos et al., 2016). As female naive cells had slightly elevated total X-linked expression compared to male naive cells, dosage compensation may still be incomplete in 5i/L/A. This provides another indication that these cells may correspond most closely to the late morula or early blastocyst.

Fourth, we used a quantitative and highly sensitive assay based on the detection of human mitochondrial DNA (Cohen et al., 2016) to rigorously assess the potential of naive human ESCs to contribute to interspecific chimera formation after injection into mouse morula- or blastocyst-stage embryos. Contribution to mouse-human chimeras has been proposed as an in vivo assay for developmental potency of naive human cells, as shown for rat ESCs (Kobayashi et al., 2010) and monkey iPSCs (Fang et al., 2014). In recent experiments, primed human ESCs distributed into different parts of the host epiblast when introduced into postimplantation mouse embryos cultured in vitro for 2 days (Mascetti and Pedersen, 2016, Wu et al., 2015). This suggested that matching the developmental stage of donor cells and host embryo may be important for integration of the cells into the embryo. In further support of this notion, when human neural crest cells were microinjected into mouse embryos at E8.5, the stage when the host neural crest cells leave the neural tube, pigmentation in post-natal chimeras revealed functional integration of the donor cells (Cohen et al., 2016). Here we injected a large number of mouse morula- or blastocyst-stage embryos (n = 2,951) with naive human cells generated in 6i/L/A, 5i/L/A, 4i/L/A, or KLF2/NANOG transgenic conditions and NHSM cells (Gafni et al., 2013). Only a small fraction of E9.5–E12.5 embryos was found to carry between one to at most five human cells per 10,000 mouse cells. Thus, while it may be possible to generate low-grade interspecies mouse-human chimeras, at least in our hands, the efficiency is currently too low for any definitive assessment of functional contribution of the human cells. Achieving efficient incorporation of human cells into mouse embryos may require “humanization” by constructing a host expressing suitable factors or niches that support the functional integration of human ESCs into interspecies chimeras.

In conclusion, we have outlined a set of molecular criteria for evaluating the naive state of human pluripotency. The gold standard for pluripotency in mouse is chimera formation. Our attempts to achieve functional integration of human pluripotent cells into interspecies chimeras were not successful, emphasizing the need for other defining criteria, such as transcriptional and epigenetic features of the human preimplantation embryo. Our results suggest that naive human ESCs provide a unique model system to dissect mechanisms of early human development that cannot be studied in mouse (due to evolutionary differences) or in conventional human ESCs (due to phenotypic drift from the embryo). For example, naive human ESCs provide a cellular model to interrogate the function of transposable elements that become activated during early human development. SVA integrants, which are highly enriched in the human cleavage embryo and appear to influence gene expression in naive human ESCs, are an evolutionary innovation of hominids. In addition, the vast majority of human KRAB-ZFPs target TEs not found in rodents and therefore have no ortholog in mouse, including the HERVH-controlling ZNF534. Naive human cells also provide a unique system to study X chromosome regulation during human embryogenesis, as both X chromosomes are actively transcribed despite upregulation of *XIST*. On the other hand, our analyses also highlight some notable differences between current naive human cells and the embryo, such as loss of methylation at imprinted DMRs and non-random X chromosome inactivation upon differentiation. These criteria provide a focus for future efforts to further refine the growth conditions for naive human cells and establish a cellular model that fully recapitulates the state of human pluripotent cells in vivo.

## Experimental Procedures

### Cell Culture

Conventional (primed) human ESC lines were maintained on mitomycin C inactivated mouse embryonic fibroblast (MEF) feeders in human ESC medium (hESM) and passaged mechanically using a drawn Pasteur pipette or enzymatically by treatment for 20 min with 1 mg/mL collagenase type IV (GIBCO), followed by sequential sedimentation steps in hESM to remove single cells. Naive human ESCs were cultured on mitomycin C-inactivated MEF feeder cells and were passaged by a brief PBS wash followed by single-cell dissociation using 3–5 min treatment with Accutase (GIBCO) and centrifugation in fibroblast medium. For conversion of pre-existing primed human ESC lines, we seeded 2 × 10^5^ trypsinized single cells on an MEF feeder layer in hESM supplemented with ROCK inhibitor Y-27632 (Stemgent, 10 μM). Two days later, medium was switched to 5i/L/A- or 4i/L/A (no IM12)-containing naive hESM. Following an initial wave of cell death, naive colonies appeared within 10 days and were expanded polyclonally using Accutase (GIBCO) on an MEF feeder layer. Naive cells generated with DOX-inducible KLF2 and NANOG transgenes (Theunissen et al., 2014) were maintained in 2i/L/DOX with optional inclusion of ROCK inhibitor Y-27632 (Stemgent, 10 μM). To adapt DOX-dependent naive cells to transgene-free culture conditions, 1 × 10^5^ single cells were seeded on an MEF feeder layer in 2i/L/DOX, and DOX was withdrawn the next day. For re-priming, semi-confluent cultures of naive cells were switched to hESM with ROCK inhibitor Y-27632 (Stemgent, 10 μM) and passaged with collagenase on MEFs. Neuronal precursor cells (NPCs) were derived from re-primed cells using a published differentiation protocol (Cohen et al., 2007). To culture naive human cells described by Gafni et al. (2013), we used the KSR-based version of the NHSM protocol. Tissue culture media were filtered using a low protein-binding 0.22 μm filter (Corning). All experiments in this paper were performed under physiological oxygen conditions (5% O_2_, 3% CO_2_). Detailed media compositions are described in Supplemental Experimental Procedures. All animal experiments were performed in compliance with protocol #1031-088-16 from the Committee on Animal Care at MIT. In addition, interspecies chimerism experiments were approved by the Embryonic Stem Cell Research Oversight (ESCRO) Committee at Whitehead Institute.

## Author Contributions

T.W.T., M.F., Y. He, D.T., J.R.E., and R.J. conceived of the study and designed experiments. T.W.T. developed naive conditions and performed tissue culture experiments. E.P., M.F., J. Duc, and D.T. developed transposcriptome methodology. E.P., A.I., J.P., and M.F. matched transposcriptome with public RNA-seq datasets. Y. He, R.C.O., R.C., Z.Z., J.R.N., and J.R.E. performed methylation analysis. J.P., M.I., and M.F performed chromatin IP analysis. H.W. performed gene targeting experiments. S.M., T.W.T., M.A.C., K.J.W., J. Drotar, and T.L. performed and analyzed embryo injections. Y. Huang performed bioinformatic analyses of X chromosome status. T.W.T., R.J., M.F., Y. He, and R.C.O. wrote the manuscript with input from D.T. and J.R.E.

## Accession Numbers

The accession number for the gene expression, methylation, and ChIP-seq analyses in this paper is GEO: GSE75868.

## Supplemental Information

Document S1. Supplemental Experimental Procedures and Figures S1–S7

Table S1. Mean Expression Counts for All TE Integrants that Yielded a Minimum of 20 Reads in at Least One ESC Sample Related to Figure 1.

Table S2. Top Ten Naive TEs with Highest Fold Change Compared with Primed ESCs Related to Figure 1.

Table S3. A Summary of Various Statistics for Methylomes Included in This Study Related to Figure 4.

Table S4. CpG Methylation Data on Candidate Imprinted DMRs Related to Figure 5.

Document S2. Article plus Supplemental Information

## References

[bib1] Bai Q., Assou S., Haouzi D., Ramirez J.M., Monzo C., Becker F., Gerbal-Chaloin S., Hamamah S., De Vos J. (2012). Dissecting the first transcriptional divergence during human embryonic development. Stem Cell Rev..

[bib2] Blakeley P., Fogarty N.M., del Valle I., Wamaitha S.E., Hu T.X., Elder K., Snell P., Christie L., Robson P., Niakan K.K. (2015). Defining the three cell lineages of the human blastocyst by single-cell RNA-seq. Development.

[bib3] Boroviak T., Loos R., Bertone P., Smith A., Nichols J. (2014). The ability of inner-cell-mass cells to self-renew as embryonic stem cells is acquired following epiblast specification. Nat. Cell Biol..

[bib4] Boroviak T., Loos R., Lombard P., Okahara J., Behr R., Sasaki E., Nichols J., Smith A., Bertone P. (2015). Lineage-specific profiling delineates the emergence and progression of naive pluripotency in mammalian embryogenesis. Dev. Cell.

[bib5] Chan Y.S., Göke J., Ng J.H., Lu X., Gonzales K.A., Tan C.P., Tng W.Q., Hong Z.Z., Lim Y.S., Ng H.H. (2013). Induction of a human pluripotent state with distinct regulatory circuitry that resembles preimplantation epiblast. Cell Stem Cell.

[bib6] Cohen M.A., Itsykson P., Reubinoff B.E. (2007). Neural differentiation of human ES cells. Curr. Protoc. Cell Biol..

[bib7] Cohen, M.A., Wert, K.J., Goldmann, J., Markoulaki, S., Buganim, Y., Fu, D., and Jaenisch, R. (2016). Human neural crest cells contribute to coat pigmentation in interspecies chimeras after in utero injection into mouse embryos. Proceedings of the National Academy of Sciences of the United States of America *113*, 1570–1575. http://dx.doi.org/10.1073/pnas.1525518113. 10.1073/pnas.1525518113 PMC4760776 26811475

[bib8] Court F., Tayama C., Romanelli V., Martin-Trujillo A., Iglesias-Platas I., Okamura K., Sugahara N., Simón C., Moore H., Harness J.V. (2014). Genome-wide parent-of-origin DNA methylation analysis reveals the intricacies of human imprinting and suggests a germline methylation-independent mechanism of establishment. Genome Res..

[bib9] De Los Angeles A., Ferrari F., Xi R., Fujiwara Y., Benvenisty N., Deng H., Hochedlinger K., Jaenisch R., Lee S., Leitch H.G. (2015). Hallmarks of pluripotency. Nature.

[bib10] Fang R., Liu K., Zhao Y., Li H., Zhu D., Du Y., Xiang C., Li X., Liu H., Miao Z. (2014). Generation of naive induced pluripotent stem cells from rhesus monkey fibroblasts. Cell Stem Cell.

[bib11] Friedli M., Trono D. (2015). The developmental control of transposable elements and the evolution of higher species. Annu. Rev. Cell Dev. Biol..

[bib12] Gafni O., Weinberger L., Mansour A.A., Manor Y.S., Chomsky E., Ben-Yosef D., Kalma Y., Viukov S., Maza I., Zviran A. (2013). Derivation of novel human ground state naive pluripotent stem cells. Nature.

[bib13] Guo H., Zhu P., Yan L., Li R., Hu B., Lian Y., Yan J., Ren X., Lin S., Li J. (2014). The DNA methylation landscape of human early embryos. Nature.

[bib14] Hancks D.C., Kazazian H.H. (2010). SVA retrotransposons: evolution and genetic instability. Semin. Cancer Biol..

[bib15] Huang K., Maruyama T., Fan G. (2014). The naive state of human pluripotent stem cells: a synthesis of stem cell and preimplantation embryo transcriptome analyses. Cell Stem Cell.

[bib16] Iyengar S., Farnham P.J. (2011). KAP1 protein: an enigmatic master regulator of the genome. J. Biol. Chem..

[bib17] Kobayashi T., Yamaguchi T., Hamanaka S., Kato-Itoh M., Yamazaki Y., Ibata M., Sato H., Lee Y.S., Usui J., Knisely A.S. (2010). Generation of rat pancreas in mouse by interspecific blastocyst injection of pluripotent stem cells. Cell.

[bib18] Lee H.J., Hore T.A., Reik W. (2014). Reprogramming the methylome: erasing memory and creating diversity. Cell Stem Cell.

[bib19] Lister R., Pelizzola M., Dowen R.H., Hawkins R.D., Hon G., Tonti-Filippini J., Nery J.R., Lee L., Ye Z., Ngo Q.M. (2009). Human DNA methylomes at base resolution show widespread epigenomic differences. Nature.

[bib20] Mascetti V.L., Pedersen R.A. (2016). Human-mouse chimerism validates human stem cell pluripotency. Cell Stem Cell.

[bib21] Matsui T., Leung D., Miyashita H., Maksakova I.A., Miyachi H., Kimura H., Tachibana M., Lorincz M.C., Shinkai Y. (2010). Proviral silencing in embryonic stem cells requires the histone methyltransferase ESET. Nature.

[bib22] Nichols J., Smith A. (2009). Naive and primed pluripotent states. Cell Stem Cell.

[bib23] Okamoto I., Patrat C., Thépot D., Peynot N., Fauque P., Daniel N., Diabangouaya P., Wolf J.P., Renard J.P., Duranthon V., Heard E. (2011). Eutherian mammals use diverse strategies to initiate X-chromosome inactivation during development. Nature.

[bib24] Pastor W.A., Chen D., Liu W., Kim R., Sahakyan A., Lukianchikov A., Plath K., Jacobsen S.E., Clark A.T. (2016). Naive human pluripotent cells feature a methylation landscape devoid of blastocyst or germline memory. Cell Stem Cell.

[bib25] Petropoulos S., Edsgärd D., Reinius B., Deng Q., Panula S.P., Codeluppi S., Plaza Reyes A., Linnarsson S., Sandberg R., Lanner F. (2016). Single-cell RNA-seq reveals lineage and X chromosome dynamics in human preimplantation embryos. Cell.

[bib26] Quenneville S., Verde G., Corsinotti A., Kapopoulou A., Jakobsson J., Offner S., Baglivo I., Pedone P.V., Grimaldi G., Riccio A., Trono D. (2011). In embryonic stem cells, ZFP57/KAP1 recognize a methylated hexanucleotide to affect chromatin and DNA methylation of imprinting control regions. Mol. Cell.

[bib27] Roode M., Blair K., Snell P., Elder K., Marchant S., Smith A., Nichols J. (2012). Human hypoblast formation is not dependent on FGF signalling. Dev. Biol..

[bib28] Rowe H.M., Jakobsson J., Mesnard D., Rougemont J., Reynard S., Aktas T., Maillard P.V., Layard-Liesching H., Verp S., Marquis J. (2010). KAP1 controls endogenous retroviruses in embryonic stem cells. Nature.

[bib29] Singh K., Cassano M., Planet E., Sebastian S., Jang S.M., Sohi G., Faralli H., Choi J., Youn H.D., Dilworth F.J., Trono D. (2015). A KAP1 phosphorylation switch controls MyoD function during skeletal muscle differentiation. Genes Dev..

[bib30] Takashima Y., Guo G., Loos R., Nichols J., Ficz G., Krueger F., Oxley D., Santos F., Clarke J., Mansfield W. (2014). Resetting transcription factor control circuitry toward ground-state pluripotency in human. Cell.

[bib31] Theunissen T.W., Powell B.E., Wang H., Mitalipova M., Faddah D.A., Reddy J., Fan Z.P., Maetzel D., Ganz K., Shi L. (2014). Systematic identification of culture conditions for induction and maintenance of naive human pluripotency. Cell Stem Cell.

[bib32] Tucker K.L., Beard C., Dausmann J., Jackson-Grusby L., Laird P.W., Lei H., Li E., Jaenisch R. (1996). Germ-line passage is required for establishment of methylation and expression patterns of imprinted but not of nonimprinted genes. Genes Dev..

[bib33] Turelli P., Castro-Diaz N., Marzetta F., Kapopoulou A., Raclot C., Duc J., Tieng V., Quenneville S., Trono D. (2014). Interplay of TRIM28 and DNA methylation in controlling human endogenous retroelements. Genome Res..

[bib34] Urich M.A., Nery J.R., Lister R., Schmitz R.J., Ecker J.R. (2015). MethylC-seq library preparation for base-resolution whole-genome bisulfite sequencing. Nat. Protoc..

[bib35] Wang J., Xie G., Singh M., Ghanbarian A.T., Raskó T., Szvetnik A., Cai H., Besser D., Prigione A., Fuchs N.V. (2014). Primate-specific endogenous retrovirus-driven transcription defines naive-like stem cells. Nature.

[bib36] Ware C.B., Nelson A.M., Mecham B., Hesson J., Zhou W., Jonlin E.C., Jimenez-Caliani A.J., Deng X., Cavanaugh C., Cook S. (2014). Derivation of naive human embryonic stem cells. Proc. Natl. Acad. Sci. USA.

[bib37] Weber M., Hellmann I., Stadler M.B., Ramos L., Pääbo S., Rebhan M., Schübeler D. (2007). Distribution, silencing potential and evolutionary impact of promoter DNA methylation in the human genome. Nat. Genet..

[bib38] Wu J., Izpisua Belmonte J.C. (2015). Dynamic pluripotent stem cell states and their applications. Cell Stem Cell.

[bib39] Wu J., Okamura D., Li M., Suzuki K., Luo C., Ma L., He Y., Li Z., Benner C., Tamura I. (2015). An alternative pluripotent state confers interspecies chimaeric competency. Nature.

[bib40] Yan L., Yang M., Guo H., Yang L., Wu J., Li R., Liu P., Lian Y., Zheng X., Yan J. (2013). Single-cell RNA-seq profiling of human preimplantation embryos and embryonic stem cells. Nat. Struct. Mol. Biol..

