# Supplementary material for: Molecular Criteria for Defining the Naive Human Pluripotent State
Source: Cell Stem Cell. 2016 Oct 6;19(4):502–15. doi: 10.1016/j.stem.2016.06.011 (PMC5065525; doi:10.1016/j.stem.2016.06.011)
Supplement: Document S1. Supplemental Experimental Procedures and Figures S1–S7 [file mmc1.pdf]

Cell Stem Cell, Volume *19*

Supplemental Information

Molecular Criteria for Defining the Naive Human Pluripotent State

Thorold W. Theunissen, Marc Friedli, Yupeng He, Evarist Planet, Ryan C. O'Neil, Styliani Markoulaki, Julien Pontis, Haoyi Wang, Alexandra Iouranova, Michaël Imbeault, Julien Duc, Malkiel A. Cohen, Katherine J. Wert, Rosa Castanon, Zhuzhu Zhang, Yanmei Huang, Joseph R. Nery, Jesse Drotar, Tenzin Lungjangwa, Didier Trono, Joseph R. Ecker, and Rudolf Jaenisch

Supplemental Information

Molecular Criteria for Defining the Naive Human Pluripotent State

Thorold W. Theunissen[1,*], Marc Friedli[2,*], Yupeng He[3,*], Evarist Planet[2], Ryan Oneil[3], Styliani Markoulaki[1], Julien Pontis[2], Haoyi Wang[1,‡], Alexandra Iouranova[2], Michaël Imbeault[2], Julien Duc[2], Malkiel A. Cohen[1], Katherine J. Wert[1], Rosa G. Castanon[3], Zhuzhu Zhang[3], Yanmei Huang[1], Joseph R. Nery[3], Jesse Drotar[1], Tenzin Lungjangwa[1], Didier Trono[2,#], Joseph R. Ecker[3,#] and Rudolf Jaenisch[1,4,#]

Contents:

- I. Supplemental Experimental Procedures
- II. Supplemental Figure Legends
- III. Supplemental Table Legends
- IV. Supplemental Figures
- V. Supplemental References

I. Supplemental Experimental Procedures

Culture conditions

Conventional (primed) human ESCs were maintained on mitomycin C inactivated mouse embryonic fibroblast (MEF) feeder layers and passaged using Collagenase (1 mg/mL) or manual methods. Primed human ESCs and human iPSCs were cultured in human ESC medium (hESM) [DMEM/F12 (Invitrogen) supplemented with 15% fetal bovine serum (Gibco HI FBS, 10082-147), 5% KnockOut Serum Replacement (Invitrogen), 2 mM L-glutamine (MPBio), 1% nonessential amino acids (Invitrogen), 1% penicillin-streptomycin (Lonza), 0.1 mM β-mercaptoethanol (Sigma) and 4 ng/ml FGF2 (R&D systems)]. Naive human ESCs were cultured on mitomycin C-inactivated MEF

feeder cells, and were passaged by a brief PBS wash followed by single-cell dissociation using 3-5 minute treatment with Accutase (Gibco) and centrifugation in fibroblast medium [DMEM (Invitrogen) supplemented with 10% FBS (Gibco HI FBS, 10082-147), 2 mM L-glutamine (MPBio), 1% nonessential amino acids (Invitrogen), 1% penicillin-streptomycin (Lonza) and 0.1 mM β-mercaptoethanol (Sigma)]. For conversion of pre-existing primed human ESC lines, we seeded 2 x 10^5 trypsinized single cells on a MEF feeder layer in hESM supplemented with ROCK inhibitor Y-27632 (Stemgent, 10 μM). 2 days later medium was switched to 5i/L/A or 4i/L/A (no IM12)-containing naive human ESC medium. Following an initial wave of cell death, naive colonies appeared within 10 days and were expanded polyclonally using Accutase (Gibco) on a MEF feeder layer. Naive human pluripotent cells were derived and maintained in serum-free N2B27-based media supplemented with inhibitors and cytokines. 500 mL of medium was generated by including: 240 mL DMEM/F12 (Invitrogen; 11320), 240 mL Neurobasal (Invitrogen; 21103), 5 mL N2 supplement (Invitrogen; 17502048), 10 mL B27 supplement (Invitrogen; 17504044), 10 μg recombinant human LIF (made in-house), 2 mM L-glutamine (MPBio), 1% nonessential amino acids (Invitrogen), 0.1 mM β-mercaptoethanol (Sigma), 1% penicillin-streptomycin (Lonza), 50 μg/ml BSA (Gibco Fraction V, 15260), and the following small molecules and cytokines: PD0325901 (Stemgent, 1 μM), IM-12 (Enzo, 1 μM or 0.5 μM), SB590885 (R&D systems, 0.5 μM), WH4-023 (A Chemtek or Selleckchem, 1 μM), Y-27632 (Stemgent, 10 μM) and Activin A (Peprotech, 10 ng/mL). Additional inhibitors described in this work include: GSK3 inhibitor CHIR99021 (Stemgent, 1 μM or 0.3 μM), JNK inhibitor SP600125 (R&D systems, 10 μM) and doxycycline (DOX) (Sigma, 2 μg/ml). Naive cells generated with DOX-inducible KLF2 and NANOG transgenes (Theunissen et al., 2014) were maintained in 1 μM PD0325901, 0.3 μM (t) or 1 μM CHIR99021, 20 ng/ml hLIF and 2 μg/ml DOX with optional inclusion of 10 μM ROCK inhibitor Y-27632 (t2i/L/DOX+RI). To adapt DOX-dependent naive cells to transgene-free culture conditions, 1 x 10^5 single cells were seeded on a MEF feeder layer in 2i/L/DOX and DOX was withdrawn the next day. For re-priming, semi-confluent cultures of naive cells were switched to hESM with ROCK inhibitor Y-27632 (10 μM) and passaged with Collagenase on MEFs. Neuronal precursor cells (NPCs) were derived from re-primed cells using a published

differentiation protocol (Cohen et al., 2007). To culture naive human cells described by Gafni et al. (2013), we used the KSR-based version of NHSM (updated version 15/12/2013 provided by Dr. Jacob Hanna's laboratory), consisting of KO-DMEM (Invitrogen) supplemented with 18% KSR (Invitrogen), 1% penicillin-streptomycin (Invitrogen), 1% L-glutamine (Invitrogen), 1% NEAA (Invitrogen), 0.1 mM β-mercaptoethanol (Sigma), 12.5 μg/mL recombinant human insulin (Sigma) and the following cytokines and inhibitors: 20 ng/mL human LIF (Peprotech), 8 ng/mL FGF2 (R&D), 2 ng/mL TGFβ1 (Peprotech), 3 μM Chir99021 (Axon Medchem), 1 μM PD0325901 (Axon Medchem), 2 μM BIRB796 (Axon Medchem), 10 μM SP600125 (Tocris), 5 μM Y-27632 (Axon Medchem) and 5 μM Go6983 (Tocris). Tissue culture media were filtered using a low protein-binding binding 0.22 μm filter (Corning). All experiments in this paper were performed under physiological oxygen conditions (5% O_2, 3% CO_2).

TALEN design and construction

TALENs were designed and assembled as previously described (Hockemeyer et al., 2011). Candidate TALENs constructs were transiently transfected into HEK293 cells, and active TALENs targeting the desired location at high efficiency were identified using a Surveyor assay. The sequences of primers used in the Surveyor assay are gcagactggcatgttctctgtg and tgctccatgagggatccttgtcc. The DNA binding sites of the TALENs used for targeting the exon 3 of MECP2 are gcagccatcagcccaccact and ctctgctttgcctgcct. To target both MECP2 alleles in WIBR2 human ESCs, two donor constructs were generated. One donor construct was engineered to contain an eGFP-polyA sequence in-frame with MECP2 exon 3 and a PGK-puro-polyA cassette flanked by two homology arms corresponding to the genomic sequence of MECP2. The other donor construct contains a tdTomato-polyA sequence along with a PGK-neo-polyA cassette, flanked by the same homology arms.

Genome editing in human ESCs

TALEN-mediated gene targeting was performed as previously described (Hockemeyer et al., 2011). Briefly, WIBR2 ESCs were cultured in Rho-associated protein kinase

(ROCK) inhibitor for 24 hours prior to electroporation. Cells were harvested and dissociated into single cells using trypsin/EDTA solution, followed by re-suspension in PBS. Electroporation was performed using 40ug of donor plasmid and 5ug of each TALEN plasmid. Cells were plated on DR4 MEF feeders, and cultured in maintenance medium containing ROCK inhibitor for the first 24 hours. Puromycin or G418 was added to maintenance medium 48 hours after electroporation, and individual resistant clones were picked and expanded. Southern blots were performed to identify correctly targeted clones, using 32P random primer-labeled probes (Stratagene) against 5' or 3' external sequences, or GFP (internal). Two independently double-targeted MECP2 reporter primed hESC clones were established. 29M-GP26-TN9 was established by targeting the first MECP2 allele using GFP (29M-GP), and then targeting the other allele with tdTomato. 29M-TN3-GP8 was established by targeting the first MECP2 allele using tdTomato (29M-TN), and then targeting the other allele with GFP.

Flow cytometry

Single cell suspensions were filtered, stained with DAPI (Life Technologies), and assessed on the LSRFortessa SORP (Beckton-Dickinson, San Jose, CA). PE-Texas Red (for detection of autofluorescence) was excited by a Coherent Compass 561 nm (50 mW) yellow/green laser and detected using a bandpass filter (emission) of 610/20, GFP was excited by a Coherent Sapphire Solid State 488 nm (100 mW) blue laser and detected using a bandpass filter (emission) of 530/30, and DAPI was excited by a Lightwave Xcyte 355 nm (60 mW) UV laser and detected using a bandpass filter (emission) of 450/50.

Interspecific embryo injections

Donor cells for interspecific embryos injection

Interspecific embryo injections were performed with the following constitutively labeled human ESC lines and culture conditions: C1 AAVS1-GFP iPSCs and WIBR3 AAVS1-GFP ESCs in KSR-based NHSM media (Gafni et al., 2013), WIBR3 AAVS1-GFP ESCs in 5i/L/A or 6i/L/A (with JNK inhibitor), WIBR3 AAVS1-tdTomato ESCs with inducible

KLF2 and NANOG transgenes in t2i/L/DOX+RI (1 µM PD0325901, 0.3 µM CHIR99021, 10 µM Y27632) or 4i/L/A after DOX withdrawal.

Morula and blastocyst injection

Embryo injections were performed using (C57Bl/6xDBA) B6D2F2 host embryos, either at the blastocyst or morula stage. For both types of injections, B6D2F1 females were hormone primed by an i.p. injection of PMS (Pregnant Mare Serum Gonadotropin, EMD Millipore) followed 46h later by an injection of hCG (human Chorionic Gonadrotropin, VWR). After mating with stud males, embryos were harvested at the one cell stage. For morula stage injections the embryos were cultured in a CO_2 incubator for 2 nights, whereas for blastocyst injections embryos were cultured for 3 nights. On the day of the injection, groups of embryos were placed in drops of M2 medium and using a 20 µm diameter injection pipet (Origio, Inc.) 3-10 cells were injected under the zona or in non-compacted embryos in the inner space of the embryo. For the injection of blastocysts, approximately 10 cells were injected into the blastocoel cavity. In both cases, the injection was assisted by an XY Clone laser (Hamilton Thorne, Inc.). About 20 blastocysts were subsequently transferred to each recipient female; the day of injection was considered as 2.5 dpc. Fetuses were collected at 9.5-12.5 dpc for further analyses. As previously, all animal experiments were performed in compliance with protocol # 1031-088-16 from the Committee on Animal Care at MIT. In addition, interspecies chimerism experiments were approved by the Embryonic Stem Cell Research Oversight (ESCRO) Committee at Whitehead Institute.

Human mitochondrial PCR assay

Genomic DNA was extracted from E9.5-12.5 injected mouse embryos. 25ng of genomic DNA per sample was used for qPCR analysis to measure the presence of human DNA. The existence of human DNA in embryos was determined by amplification of a human-specific mitochondrial (mtDNA) fragment. To correct for sample-to-sample variations in qPCR efficiency and errors in sample quantification the amplification of a specific ultra-conserved non-coding element (UCNE) was used as invariant endogenous control. All

samples were run in technical triplicates in a 384-well plate with Fast SYBR Green Master Mix (Life Technologies) using an ABI Prism 7900HT Sequence detection system (Life Technologies). Relative quantification ratios were calculated and determined by $R=2^{-\Delta\Delta Ct}$. For all experimental samples, a relative quantification (RQ) was measured in comparison to negative-control mouse DNA samples. In addition, for calibration, each assay contained the human:mouse cell serial dilutions. Additional details of this protocol are described in (Cohen et al., 2016).

qRT-PCR

Total RNA was isolated using the Rneasy Kit (QIAGEN) and reversed transcribed using the Superscript III First Strand Synthesis kit (Invitrogen). Quantitative RT-PCR analysis was performed in triplicate using the ABI 7900 HT system with FAST SYBR Green Master Mix (Applied Biosystems). Gene expression was normalized to a reference gene (RPLP0). Error bars represent the standard deviation (SD) of the mean of triplicate reactions. Primer sequences are included in the following primer table:

Primers used in this study:

**Gene**	**Primer sequence (5'- 3')**	**Application**
ZIC2-F	CCCTTCAAGGCCAAATACAA	RT-PCR
ZIC2-R	TGCATGTGCTTCTTCCTGTC	
STELLA-F	GTTACTGGGCGGAGTTCGTA	RT-PCR
STELLA-R	TGAAGTGGCTTGGTGTCTTG	
REX1-F	GGAATGTGGGAAAGCGTTCGT	RT-PCR
REX1-R	CCGTGTGGATGCGCACGT	
FUW-KLF2-F	GATTTTGCTGGGTTGGTTTTT	RT-PCR
FUW-KLF2-R	CCACATAGCGTAAAAGGAGCA	
FUW-NANOG-F	GCTGGGGAAGGCCTTAATGT	RT-PCR
FUW-NANOG-R	CCACATAGCGTAAAAGGAGCA	

GAPDH-F	CGAGATCCCTCCAAAATCAA	RT-PCR
GAPDH-R	ATCCACAGTCTTCTGGGTGG	
RPLP0-F	GCTTCCTGGAGGGTGTCC	RT-PCR
RPLP0-R	GGACTCGTTTGTACCCGTTG	

RNA-Seq analysis

To prepare RNA for sequencing, 1 million naive or primed human ESCs were trypsinized and purified from GFP-labeled or non-fluorescent MEFs using the FACSAria (Beckton-Dickinson) prior to lysis and RNA extraction. Samples were homogenized in 1 ml of TRIzol Reagent (Life Technologies, 15596-026), purified using the mirVANA miRNA isolation kit (Ambion, AM1560) following the manufacturer’s instructions and re-suspended in 100 µl nuclease-free water (Ambion, AM9938). Reads were mapped to the human genome (hg19) using TopHat (v2.0.11) in sensitive mode (the exact parameters are: tophat --library-type fr-firststrand -g 1 --no-novel-juncs --no-novel-indels -G $gtf --transcriptome-index $ transcriptome --b2-sensitive -o $localdir $index $reads1 $reads2). Gene counts were generated using HTSeq-count. For repetitive sequences, an in-house curated version of the Repbase database was used (fragmented LTR and internal segments belonging to a single integrant were merged). TEs counts were generated using the multiBamCov tool from the bedtools software. TEs which did not have at least one sample with 20 reads (after normalizing for sequencing depth) were discarded from the analysis. TEs overlapping exons were also removed from the analysis. Normalisation for sequencing depth and differential gene expression analysis was performed using Voom (Law et al., 2014) as it has been implemented in the limma package of Bioconductor (Gentleman et al., 2004). A gene (or TE) was considered to be differentially expressed when the fold change between groups was bigger than 2 and the p-value was smaller than 0.05. A moderated paired t-test (as implemented in the limma package of R) was used to test significance. P-values were corrected for multiple testing using the Benjamini-Hochberg’s method (Benjamini, 1995). Single cell RNA-Seq data was downloaded from GEO (Edgar et al., 2002) (GSE36552). Data was mapped and processed as explained above. When merging with in-house RNA-Seq, only TEs which were expressed in both datasets were used. To be considered expressed, the TE needed to have at least as many reads across all samples as samples existed in each dataset.

Transposon expression profiling was performed on the following primed cell lines: male WIBR1 hESCs (Lengner et al., 2010), male NPC 1.1#4 and NPC 1.1#13 hiPSCs

(Maetzel et al., 2014), and two female primed WIBR2 hESC clones independently targeted with fluorescent reporters at both alleles of MECP2 (29M-GP26-TN9 = GFP-positive in primed state; 29M-TN3-GP8 = tdTomato-positive in primed state). Transposon expression profiling was performed on the following naive cell lines: male WIN1 hESCs derived directly from the human embryo (Theunissen et al., 2014) and maintained in 5i/L/A for 19 passages, double-color WIBR2 MECP2 reporter cells derived in 5i/L/A for 10 passages, WIBR2 and WIBR3 naive hESCs derived in 4i/L/A for 4 passages and two clones (#12 and #16) of WIBR3 OCT4-ΔPE-GFP-positive naive cells maintained with DOX-inducible KLF2 and NANOG transgenes in t2i/L/DOX+RI. In addition, we performed transposon profiling on three additional lines cultured in KSR-based NHSM media on MEFs (Gafni et al., 2013): WIBR2 ESCs cultured in NHSM for 13 passages, C1 AAVS1-GFP iPSCs cultured in NHSM for 19 passages and LIS2 ESCs cultured in NHSM for 38 passages. C1 AAVS1-GFP iPSCs (P9 NHSM) and LIS2 ESCs (P35 NHSM) were shared by Dr. Jacob Hanna's lab (Weizmann Institute, Rehovot, Israel) and maintained in NHSM for 10 and 3 additional passages, respectively, before FACS purification to remove MEFs and RNA isolation.

Correspondence between naive/primed ESCs and single cell expression data from human embryonic stages

For every cell state we perform a statistical test to find the genes (or TEs) that have a different expression level compared to the other cell states (Yan et al., 2013). For this we use a moderated F-test (comparing the interest group against every other) as implemented in the limma package of Bioconductor. For a gene (or TE) to be selected as expressed in a specific cell state it needs to have a significant p-value (<0.05 after adjusting for multiple testing with the Benjamini and Hochberg method) and an average fold change respective to the other cell states greater than 10. Note that with this approach a gene (or TE) can be marked as expressed in more than one cell state.

Once we have the genes (or TEs) that are expressed in a specific cell state we ask if those genes are more expressed in primed or in naive. For that we see how many of the genes are up (or down) regulated in the primed/naive pairwise comparison. For a gene

to be considered differentially expressed it needs to have a p-value (after multiple testing correction with the Benjamini and Hochberg method) lower than 0.05 and a fold change greater than 2.

Genome-wide SNP Genotyping

Affymetrix human SNP array 6.0 was used to interrogate SNP genotypes of the WIBR2 and WIBR3 cell lines. For WIBR2, data were generated by hybridizing genomic DNA to the array according to manufacturer’s instructions. For WIBR3, data were obtained from a previous study (Soldner et al., 2011) deposited in GEO (GSM738141) that analyzed copy number variations (CNVs) rather than SNP genotypes. Array intensity data were analyzed by Affymetrix Genotyping Console. The BIRDSEED V2 algorithm and the default confidence threshold (0.1) were used for genotyping call. Five additional arrays from the same previous study (Soldner et al., 2011) generated from similar cell lines using the same platform (GSM738137, GSM738138, GSM738139, GSM738140, and GSM738142) were included in the analysis to boost sensitivity of the genotyping call.

Quantification of allele-specific gene expression

Genotype information for SNPs on X chromosome was obtained using the Affymetrix human SNP array 6.0 as described above. SNPs found to be heterozygous in WIBR2, i.e. having an “AB” genotype, were selected for further analysis (5044 such SNPs were found on X chromosome). To determine allele-specific gene expression, 2x100 paired-end sequencing reads of RNAs isolated from WIBR2 single color MECP2$^{GFP\text{-}ON/Tom\text{-}OFF}$ or MECP2$^{GFP\text{-}OFF/Tom\text{-}ON}$ primed cells and double color naive cells derived in 5i/L/A and maintained in 5i/L/A or 4i/L/A were mapped to the human reference genome hg38 using STAR (Dobin et al., 2013). Read depths of the two different alleles “A” and “B” at each of the SNP sites were analyzed for the RNA-seq data using samtools mpileup (Li, 2011). A stringent filter, requiring a total read depth of at least 10 in all samples was applied to select SNPs that can be used to reliably derive allelic expression information. Relative allelic expression level was calculated as number of reads originating from a

particular allele divided by total number of reads. Genes reported to escape X inactivation are labeled in red: CD99 and ZBED1 are positioned in the PAR region, while PIR is listed as Discordant in the consensus definition (Balaton et al., 2015).

Quantification of total X-linked gene expression

RNA-seq paired-end reads of 100 nt were aligned to human reference genome hg38 using STAR (Dobin et al., 2013). Gene expression was quantified using cufflinks (Roberts et al., 2011). Genes on X chromosome having an expression level of greater than one FPKM in at least one sample were selected for analysis. Selected genes were arranged according to their order on the X chromosome. For each gene, the average of the FPKM value in male naive samples was used as a baseline; Log2(FoldChange) was calculated for each individual sample compared to the baseline. Medians of the log2(FoldChange) values of a moving window of 50 genes were plotted. The quantification of total X-linked gene expression levels was performed using single-color MECP2 reporter female primed lines (n=2), male primed lines maintained in the same growth conditions (n=3), double-color MECP2 reporter naive cells obtained by conversion in 5i/L/A and maintenance in 5i/L/A, 4i/L/A or 4i/L/A+CHIR99021 for 10 passages (n=3) and naive male embryo-derived WIN1 ESCs maintained in 5i/L/A or 4i/L/A (n=2). Note that in this analysis all samples were normalized to the average expression in male naive samples, whereas in our previous study (Theunissen et al., 2014) we performed a similar analysis using male primed cells as baseline.

ChIP-Seq

Cells were cross-linked for 10 minutes at room temperature by the addition of one-tenth of the volume of 11% formaldehyde solution (11% Formaldehyde Sigma 2525459, 50mM HEPES pH 7.5, 100 mM NaCl, 1 mM EDTA pH 8.0, 0.5 mM EGTA pH 8.0) to the growth media followed by quenching with 100 mM glycine. Cells were washed twice with PBS, then the supernatant was aspirated and the cell pellet was flash frozen in liquid nitrogen.

Pellets were lysed, resuspended in 1mL of LB1 on ice for 10min (50 mM HEPES-KOH pH 7.4, 140 mM NaCl, 1 mM EDTA, 0.5 mM EGTA, 10% Glycerol, 0.5% NP40, 0.25% Tx100, protease inhibitors), then after centrifugation resuspend in LB2 on ice for 10min (10 mM Tris pH 8.0, 200 mM NaCl, 1 mM EDTA, 0.5 mM EGTA and protease inhibitors). After centrifugation, resuspend in LB3 (10 mM Tris pH 8.0, 200 mM NaCl, 1 mM EDTA, 0.5 mM EGTA, 0.1% NaDOC, 0.1% SDS and protease inhibitors) and sonicated (Covaris settings: 5% duty, 200 cycle, 140 PIP, 60 min), yielding genomic DNA fragments with a bulk size of 100-300 bp.

Coating of the beads with the specific antibodies (KAP1 ab10483 and H3K9me3 Diagenode pAb-056-050) was carried out during the day at 4°C, then chromatin was added overnight at 4°C. Subsequently, washes were performed with 3x Wash Buffer (100 mM Tris pH 8.0, 1 mM EDTA, 0.5 mM EGTA, 500 mM LiCl, 1% NP40, 1% NaDOC) and 1 with TE buffer. Final DNA was purified with Qiagen Elute Column.

Up to 10 nanograms of ChIPed DNA or input DNA (Input) were prepared for sequencing. Library was quality checked by DNA high sensitivity chip (Agilent). Quality controlled samples were then quantified by picogreen (Qubit® 2.0 Fluorometer,Invitrogen). Cluster amplification and following sequencing steps strictly followed the Illumina standard protocol.

Sequenced reads were de-multiplexed to attribute each read to a DNA sample and then aligned to reference human genome hg19 with bowtie2 (v2.2.4 with parameters -k 1 --end-to-end). PCR duplicates removal (MarkDuplicates using picard tools v1.8 and parameters: VALIDATION_STRINGENCY=LENIENT REMOVE_DUPLICATES=true), samples were downsampled (DownsampleSam using picard tools v1.8) to the lowest dataset count for H3K9me3 and KAP1 (app. 23 millions reads) and for H3K4me3 used from (Theunissen et al., 2014) (app. 9 millions reads).

Heatmaps and profile averages were calculated using ngs.plot R package (Shen et al., 2014) over 5/20kb windows around the 5' of repeat elements from BAM files (Repbase

X version), (Parameters used with ngs.plot -SC global -I 0 -L 2500 -MQ0 -RB 0.05). Datasets are normalized to their respective Input DNA sample. Screenshots were made with bigwig file produce with MACS1.4 and every scale has been adjusted to the same value.

MethylC-Seq library construction

Primed methylomes were mapped in WIBR1, WIBR2, WIBR3, WIBR3 AAVS1-GFP and previously analyzed H9 ESCs (Takashima et al., 2014). Naive methylomes were mapped in WIBR3 (AAVS1-GFP) ESCs in 5i/L/A (P21), WIN1 naive embryo-derived ESCs in 5i/L/A (P15), WIBR2 and WIBR3 ESCs in 4i/L/A (P4), two lines of WIBR3 OCT4-ΔPE-GFP DOX-inducible KLF2+NANOG naive cells (#12 and #16), and previously analyzed H9 naive ESCs maintained in t2i/L+PKCi (Takashima et al., 2014). Additional methylomes were mapped in re-primed cells (P12 and P19, respectively) obtained from WIBR3 4i/L/A naive cells and WIBR3 (AAVS1-GFP) 5i/L/A naive cells and neural precursors derived from WIBR3 4i/L/A re-primed cells.

To prepare DNA for MethylC-Seq, 2 million naive or primed human ESCs were trypsinized and purified from GFP-labeled or non-fluorescent MEFs using the FACSAria (Beckton-Dickinson). Genomic DNA was extracted using the DNeasy Blood and Tissue Kit (Qiagen, Valencia, CA). 2 µg of genomic DNA was spiked with 10 ng unmethylated cl857 Sam7 Lambda DNA (Promega, Madison, WI). The DNA was fragmented with a Covaris S2 (Covaris, Woburn, MA) to 150-200 bp, followed by end repair and addition of a 3’ A base. Cytosine-methylated adapters provided by Illumina (Illumina, San Diego, CA) were ligated to the sonicated DNA at 16˚C for 16 hours with T4 DNA ligase (New England Biolabs). Adapter-ligated DNA was isolated by two rounds of purification with AMPure XP beads (Beckman Coulter Genomics, Danvers, MA). Adapter-ligated DNA (≤450 ng) was subjected to sodium bisulfite conversion using the MethylCode kit (Life Technologies, Carlsbad, CA) as per manufacturer’s instructions. The bisulfite-converted, adapter-ligated DNA molecules were enriched by 4 cycles of PCR with the following reaction composition: 25 µL of Kapa HiFi Hotstart Uracil+ Readymix (Kapa Biosystems, Woburn, MA) and 5 µl TruSeq PCR Primer Mix (Illumina) (50 µl final). The

thermocycling parameters were: 95˚C 2 min, 98˚C 30 sec, then 4 cycles of 98˚C 15 sec, 60˚C 30 sec and 72˚C 4 min, ending with one 72˚C 10 min step. The reaction products were purified using AMPure XP beads. Up to two separate PCR reactions were performed on subsets of the adapter-ligated, bisulfite-converted DNA, yielding up to two independent libraries from the same biological sample.

MethylC-Seq data processing

MethylC-Seq data were processed as described in Schultz et al. (Schultz et al., 2015) with some modification. Briefly, adapter sequences on MethylC-Seq reads were trimmed using Cutadapt (version 1.2.1) (Martin, 2011). Next, cytosines (Cs) in the trimmed reads were computationally converted to thymines (Ts). The converted reads were mapped twice, to the forward strand of a converted Watson strand reference and the reversed strand of a converted Crick strand reference. A converted reference is created by replacing all cytosines with thymines (Watson strand) or by replacing all guanines with adenines (Crick strand). For mapping single-end reads, Bowtie 2 (version 2.2.5) (Langmead and Salzberg, 2012) was used along with default options. For aligning paired-end reads, bowtie 2 (version 2.2.5) was used with following options: "-k 2 -q --no-mixed --no-discordant --maxins 1000 --score-min L,0,-0.2". Reads were mapped to hg19 reference genome. Any read that mapped to multiple locations was removed and one read from each starting location on each strand from each library was kept (i.e., clonal reads were removed).

To identify the sites showing evidence of methylation (i.e. methylated sites), we first counted the number of reads that supported methylation and the number of reads covering. Then, binomial test was performed on these counts to test whether the methylation counts are beyond bisulfite non-conversion events. In the test, the probability of observing methylation in null hypothesis, which is that methylation observed comes from incomplete bisulfite conversion, equals the non-conversion rate, which was determined by computing the fraction of methylated reads in the lambda genome which was spiked in during library construction. For samples (Naive H9 and Primed H9 ESCs from Takashima et al. (Takashima et al., 2014) without unmethylated

DNA spiked in, the genome-wide non-CG methylation level was used as an estimate of non-conversion rate. The false discovery rate (FDR) was computed using Benjamini-Hochberg method and p-value cutoff was calculated given 1% FDR. Considering that the p-value distributions for each methylation context are different, this procedure was applied to each three nucleotide context independently (e.g., a p-value cutoff was calculated for CAC cytosines). AnnoJ browser (Wang et al., 2013) was used for visualization of MethylC-Seq data.

Reduced representation bisulfite sequencing (RRBS) data processing

RRBS reads were first trimmed using trim_galore [http://www.bioinformatics.babraham.ac.uk/projects/trim_galore/] with options "--rrbs -paired -trim1". Then, trimmed reads were mapped to hg19 human reference genome and lambda genome together by bowtie2 using the bismark pipeline (--bowtie2 -X 1000). Bisulfite non-conversion rate was estimated in the same way as in MethylC-seq data. In Figure S4A only CpG sites covered in at least one of the two RRBS experiments (8 cells and morula) (Guo et al., 2014) were included, i.e. CpG sites covered in MethylC-seq but not in RRBS were excluded.

Calculation of methylation levels

Methylation level for single site is defined as the fraction of reads supporting methlyation out of reads that cover the site subtracting bisulfite non-conversion rate. For region with multiple sites, methylation level is defined as weighted methylation (Schultz et al., 2012) subtracting bisulfite non-conversion rate. CpG methylation level is also called as mCpG/CpG, which non-CpG methylation level is named as mCpH/CpH (where H = A, C or T).

Methylation levels across various genomic features

Figures S4C and S4D show the methylation levels across multiple genomic features. Exon and intron annotation was based on GENCODE (Harrow et al., 2012) annotation

(Release 19). Promoters are defined as +/- 1kb regions around transcription start sites of all genes from GENCODE annotation. CpG island (CGI) coordinates were downloaded from UCSC genome browser (Dec 1st, 2015). Enhancer center list was downloaded from Xie et al. (Xie et al., 2013). For each enhancer center, we extended 1kb on each side and then liftover the coordinates (hg18) to hg19 reference. Repeat annotation was obtained from Repbase. For Figure S4C-D, only regions that were covered in all samples were included in the calculation.

Methylation of transposable elements (TEs)

Figure 4C-E compared the methylation of overexpressed TEs with non-overexpressed TEs from the same family in naive versus primed cells or vice versa. TEs showing significantly increased expression were considered as overexpressed copies (1% FDR, See “RNA-Seq analysis” for details). Other integrants in the same family were used as control. Only integrants that were covered in all samples were included.

Analysis of imprinted Differentially Methylated Regions (DMRs)

A list of candidate imprinted DMRs was obtained from Table 1 in Court et al. (Court et al., 2014), excluding placenta-specific imprinted DMRs. In total, 50 candidate imprinted DMRs were included. For analysis on samples related to 4i/L/A condition (Figure 5B and C; Figure S5A, C and E), we filtered out imprinted DMRs with extreme CpG methylation level (less than 0.3 or greater than 0.7) in primed WIBR3, since imprinted DMRs should have an intermediate methylation level (here defined as CpG methylation level between 0.3 and 0.7). In total, 26 imprinted DMRs were included in more advanced analyses. For analysis on samples related to 5i/L/A condition (Figure 5B and D; Figure S5B, D and F), we filtered out imprinted DMRs with extreme CpG methylation level in the AAVS1-GFP targeted subclone of primed WIBR3, resulting in 28 imprinted DMRs. In Figure 5B, “Erased” is defined as regions where the intermediate methylation in primed WIBR3 (or AAVS1-GFP targeted subclone) becomes hypomethylated (mCpG/CpG < 0.3) in both naive and re-primed cells.

For analysis of imprinted DMRs in primed H9 ESCs from Takashima et al. (Takashima et al., 2014), we started with the same 50 candidate imprinted DMRs and filtered out regions that were not intermediately methylated in primed H9, i.e. mCpG/CpG is either greater than 0.7 or less than 0.3. After filtering, 31 imprinted DMRs remained. Out of the 31 regions, 24 regions showed less than 0.3 in CpG methylation level in naive H9 ESCs. Therefore, in total, 24 out 31 imprinted DMRs in primed H9 were lost in naive H9 ESCs using the conditions described in Takashima et al. (2014).

To plot the methylation changes in imprinted DMRs and flanking regions (Figure S5A and B), each imprinted DMR along with its upstream 1kb region and downstream 1kb region were equally divided into 10 bins, respectively (30 bins in total). Next, we calculated and plotted the CpG methylation level of each bin. In Figure S5C and D, instead of CpG methylation levels, we calculated the $\log_2$ fold change of CpG methylation levels compared to the parental primed line. Specifically, for each bin, we calculated the ratio of CpG methylation level in one specific sample to the CpG methylation level of the corresponding bin in the parental primed line. In Figure S5E and F, for each sample, we plotted the distribution of the $\log_2$ fold change of bins in imprinted DMRs and flanking regions respectively. The boxplots clearly showed that in naive cells, bins in both imprinted DMRs and flanking regions were generally hypomethylated compared to the parental primed cells, while the methylation decrease in imprinted DMRs was greater. In re-primed and neural precursor cells (NPCs), the bins in flanking regions were able to restore methylation to similar levels as in the parental primed cells, but methylation in bins in imprinted DMRs failed to fully restore.

Analysis of allelic CpG methylation in imprinted DMRs

To decode allelic methylation patterns, MethylC-seq reads were first assigned to alleles based on the overlaps between basecalls on reads and WIBR3 heterozygous SNPs on the genome. Reads overlapping multiple heterozygous SNPs were discarded. Bisulfite conversion was taken into account:

- When distributing reads mapped to Watson strand to alleles, we only considered non C-T SNPs (i.e. we ignored heterozygous SNPs that have C and T as alleles).

Then, both C and T basecalls on reads are both considered as matched to Cs on the genome because under bisulfite conversion, they correspond to methylated and unmethylated cytosines respectively.

- Similarly, for reads mapped to crick strand, only non G-A SNPs were used to determinate their original allele and consider both G and A basecalls on reads as matches to Gs on the genome.

Across all CpGs within the 26 primed WIBR3 imprinted DMRs, only two CpGs (chr15:25,069,480−25,069,481) were found to be covered by reads that could be assigned to alleles and at least 5 reads in each allele of this CpG pair in all samples included in the allelic methylation analysis: WIBR3 4i/L/A, WIBR3 5i/L/A, Re-primed WIBR3 4i/L/A, Re-primed WIBR3 5i/L/A, NPC Re-primed WIBR3 4i/L/A and Primed WIBR3. Then, we calculated the allelic CpG methylation difference (Figure S5G), defined as the CpG methylation difference between alleles.

CpG methylation states of X chromosome

In Figure 6A, CGI coordinates were downloaded from UCSC genome browser and promoters are +/- 1kb regions around transcription start sties of X-linked genes. Figure 6A and Figure S6A show the CGIs with at least 1bp overlap with promoter. In Figure 6B and Figure S6B, promoters that do not overlap any CGI are shown. In this analysis, gene annotation from UCSC genome browser was used to be consistent with the annotation used in RNA-Seq analysis. In the lower panel of Figure 6B, random 2kb bins were chosen from regions outside CGI and non-CGI promoters.

II. Supplemental Figure Legends

Figure S1. Titration of GSK3 inhibitor IM12 and correspondence of naive and primed cells to human embryonic stages by gene or TE expression [related to Figures 1-2]

(**A**) Phase images (Top) and flow cytometric analysis (Bottom) of the proportion of OCT4-ΔPE-GFP+ cells obtained after withdrawal of DOX and culture for 9 passages at low density splitting (1:10) in four distinct growth conditions: 2i/L, 5i/L/A, t5i/L/A (4i/L/A + 0.5 µM IM12) and 4i/L/A (-IM12). Wild-type WIBR3 and WIBR3 OCT4-ΔPE-GFP primed cells are included for comparison.

(**B**) Quantification of the cell number after two passages at low density splitting (1:10) following DOX withdrawal and expansion in 5i/L/A, t5i/L/A (4i/L/A + 0.5 µM IM12) and 4i/L/A (-IM12). Note that reduction or removal of the GSK3 inhibitor IM12 enhances the proliferation of naive human cells in a dose-dependent manner. Error bars indicate ± 1 SD.

(**C**) Quantitative gene expression analysis for exogenous *KLF2*, exogenous *NANOG* and endogenous *ZIC2*, *REX1* and *STELLA* in primed human ESCs and naive human ESCs maintained in 2i/L/DOX, t5i/L/A (4i/L/A + 0.5 µM IM12) and 4i/L/A. Error bars indicate ± 1 SD.

(**D-E**) Dotplots showing the expression of human embryo stage-specific genes (**D**) or TEs (**E**) in naive and primed human ESCs. Using single cell RNA-Seq data from early human embryos (Yan et al., 2013), a statistical test was performed to find the genes (or TEs) that have a different expression level for every stage of human embryonic development compared to the other stages. Stage-specific genes (or TEs) that are upregulated (p-value < 0.05, fold change 2) in naive or primed cells are indicated in orange and blue, respectively, while genes (or TEs) that did not change expression are indicated in grey. The naive samples include all three conditions of naive cells that we examined in the PCA analyses shown in Figure 2A-B (i.e. 5i/L/A, 4i/L/A and t2i/L/DOX+RI). See Supplemental Experimental Procedures for details of the analysis.

Figure S2. Evaluating the transposcriptome in alternative conditions for naive human pluripotency [related to Figures 1-2]

(**A**) RNA-Seq quantification of LTR7 integrants used as naive-like reporters by Izsvák and colleagues (Wang et al., 2014) in naive or primed ESCs. The naive samples include all three conditions of naive cells that we examined in the PCA analyses shown in Figure 2A-B (i.e. 5i/L/A, 4i/L/A and t2i/L/DOX+RI).

(**B**) Correspondence between gene expression (Left) or TE expression (Right) in naive/primed ESCs described by Smith and colleagues (Takashima et al., 2014) and single cell human embryonic stages (Yan et al., 2013). For every stage of human embryonic development a statistical test was performed to find the genes (or TEs) that have a different expression level

compared to the other stages. The proportions of developmental stage-specific genes (or TEs) that are upregulated (p-value < 0.05, fold change 2) in t2i/L+Gö or primed cells are indicated in orange and blue, respectively, while genes (or TEs) that did not change expression are indicated in grey. See Supplemental Experimental Procedures for details of the analysis.

(**C**) Correspondence between gene expression (Left) or TE expression (Right) in naive ESCs cultured in KSR-based NHSM conditions as described by Hanna and colleagues (Gafni et al., 2013) and single cell human embryonic stages (Yan et al., 2013). For every stage of human embryonic development a statistical test was performed to find the genes (or TEs) that have a different expression level compared to the other stages. The proportions of developmental stage-specific genes (or TEs) that are upregulated (p-value < 0.05, fold change 2) in NHSM or primed cells are indicated in orange and blue, respectively, while genes (or TEs) that did not change expression are indicated in grey. See Supplemental Experimental Procedures for details of the analysis.

Figure S3. Expression of trophectoderm-associated transcription factors in naive human ESCs [related to Figure 2]

RNA-Seq quantification of 8 transcription factors that are upregulated in naive human ESCs and were previously reported to be overexpressed in trophectoderm and placenta compared to conventional human ESCs (Bai et al., 2012).

Figure S4. Genome-wide DNA demethylation in naive human ESCs [related to Figure 4]

(**A**) Genome-wide CpG methylation levels of all samples using regions covered by Reduced Representation Bisulfite Sequencing (RRBS) to enable incorporation of RRBS-generated 8 cell and morula methylome datasets (Guo et al., 2014).

(**B**) Genome-wide non-CpG methylation levels in naive and primed human ESCs.

(**C-D**) Two bar charts showing the (**C**) CpG and (**D**) non-CpG methylation levels of various genomic features in all samples. The order of samples in each bar subplot is the same as shown in the figure legend on the left.

Figure S5. Disruption of imprinted DMRs in naive human ESCs [related to Figure 5]

(**A-B**) Heatmaps showing the CpG methylation levels of imprinted DMRs and the flanking regions (+/- 1kb) in WIBR3 primed ESCs before and after conversion in 4i/L/A (**A**) or WIBR3 (AAVS1-GFP targeted subclone) ESCs before and after conversion in 5i/L/A (**B**) together with re-primed derivatives and differentiated NPC (neural progenitor cells). Upstream and downstream flanking regions were divided into 10 100bp bins respectively while imprinted DMRs were equally divided into 10 bins. Imprinted DMRs highlighted in blue are ones that do not lose DNA methylation in re-primed cells but it is unclear whether the allelic methylation patterns in imprinted DMRs are maintained.

(**C-D**) Heatmaps showing CpG methylation levels $\log_2$ fold change of imprinted DMRs and the flanking regions (+/- 1kb) in naive ESCs converted in 4i/L/A and their re-primed and neural derivatives compared to the parental WIBR3 primed ESCs (**C**) or naive ESCs converted in 5i/L/A and their re-primed derivatives compared to the parental WIBR3 (AAVS1-GFP targeted subclone) primed ESCs (**D**).

(**E**) Boxplot displaying the CpG methylation levels $\log_2$ fold change of bins within imprinted DMRs (blue) and within flanking regions (grey) in 4i/L/A and derivative re-primed cells and neural precursors. Similar to (C), $\log_2$ fold change was calculated compared to the parental WIBR3 primed ESCs. Bold line in the center of box indicates the median and notch around it shows the confidence interval (calculated by the "boxplot" function in R). Whiskers indicate maximum or minimum values within 1.5 interquartile range to upper (or lower) quartile.

(**F**) Boxplot displaying the CpG methylation levels $\log_2$ fold change of bins within imprinted DMRs (blue) and within flanking regions (grey) in 5i/L/A and derivative re-primed cells. Similar to (D), $\log_2$ fold change was calculated compared to the parental WIBR3 (AAVS1-GFP targeted) primed ESCs. Bold line in the center of box indicates the median and notch around it shows the confidence interval (calculated by the "boxplot" function in R). Whiskers indicate maximum or minimum values within 1.5 interquartile range to upper (or lower) quartile.

(**G**) Allelic CpG methylation levels of region with two CpGs (chr15:25,069,480-25,069,481), which is within imprinted DMR near *SNRPN* gene.

Figure S6. Methylation status of the X chromosome and double-color MECP2 reporter system [related to Figure 6]

(**A-C**) Boxplots showing CpG methylation levels at (**A**) X-linked promoter CpG islands (CGIs), (**B**) non-CGI promoter regions and (**C**) random 2 kb bins in naive and primed human ESCs. The random 2kb bins do not overlap any CGIs or non-CG promoters. Promoters are defined as +/- 1kb regions around transcription start sites. Bold line in the center of box indicates the median and notch around it shows the confidence interval (calculated by the “boxplot” function in R). Whiskers indicate maximum or minimum values within 1.5 interquartile range to upper (or lower) quartile.

(**D**) Strategy for establishing a dual color reporter system of X chromosome status in human ESCs by TALEN-mediated targeting of the two alleles of MECP2 with tdTomato and GFP sequences.

(**E**) Southern blot confirming the generation of double-targeted MECP2 reporter cells with MECP2exon3-Tomato-PGK-NEO and MECP2exon3-GFP-PGK-PURO donor vectors.

(**F**) Phase and fluorescence images of MECP2$^{GFP\text{-}ON/Tom\text{-}OFF}$ primed cells in hESM, and after conversion to the naive state in 5i/L/A and 4i/L/A. Note that the maximum intensity of MECP2-GFP is significantly weaker than MECP2-tdTomato (see also Figure S6G) and is more readily discerned by fluorescence microscopy in dome-shaped naive colonies than flat primed colonies.

(**G**) Histograms reporting the levels of MECP2-GFP and MECP-tdTomato in MECP2$^{GFP\text{-}ON/Tom\text{-}OFF}$ primed cells in hESM, and after conversion to the naive state in 5i/L/A and 4i/L/A. Note that the maximum intensity of MECP2-GFP is significantly weaker than MECP2-tdTomato.

Figure S7. Further characterization of X chromosome status in naive human ESCs [related to Figure 6]

(**A**) Flow cytometric analysis showing the distribution of MECP2-GFP and MECP-tdTomato in MECP2$^{GFP\text{-}ON/Tom\text{-}OFF}$ primed cells in hESM, and after conversion to the naive state in 5i/L/A and 4i/L/A.

(**B**) Quantification of total X-linked gene expression using a moving median plot in single-color MECP2 reporter female primed lines (n=2, yellow), male primed lines maintained in the same growth conditions (n=3, red), double-color MECP2 reporter naive cells obtained by conversion in 5i/L/A and maintenance in 5i/L/A, 4i/L/A or 4i/L/A+CHIR99021 for 10 passages (n=3, blue) and naive male embryo-derived WIN1 ESCs in 5i/L/A or 4i/L/A (n=2, green). For all samples,

the X-linked expression level is normalized to that of male naive cells. Note that in our previous study (Theunissen et al., 2014), a similar analysis was performed using male primed cells as the baseline. This graph indicates that naive and primed human ESCs have different transcriptional outputs.

(**C**) Comparison of total X-linked gene expression in double-color MECP2 reporter naive cells obtained by conversion in 5i/L/A and maintenance in 5i/L/A, 4i/L/A or 4i/L/A+CHIR99021 for 10 passages (n=3, red) and, as controls in the same cell state, naive male ESCs (WIN1) maintained in 5i/L/A or 4i/L/A (n=2, green). Note that female naive cells have slightly increased X-linked gene expression when compared to male naive control cells.

(**D**) Normalized levels of *XIST* expression in RNA-Seq analysis of MECP2 reporter single-color primed cells and double color naive cells obtained by conversion in 5i/L/A and maintained in 5i/L/A or 4i/LA for 10 passages. Male ESCs grown in the same conditions (primed and naive) were used for comparison.

III. Supplemental Table Legends

Supplemental Table 1. Mean expression counts for all TE integrants that yielded a minimum of 20 reads in at least one ESC sample **[related to Figure 1]**.

Supplemental Table 2. Top 10 naive TEs with highest fold change compared with primed ESCs **[related to Figure 1]**.

Supplemental Table 3. A summary of various statistics for methylomes included in this study **[related to Figure 4]**.

Supplemental Table 4. CpG methylation data on candidate imprinted DMRs **[related to Figure 5]**.

IV. Supplemental Figures

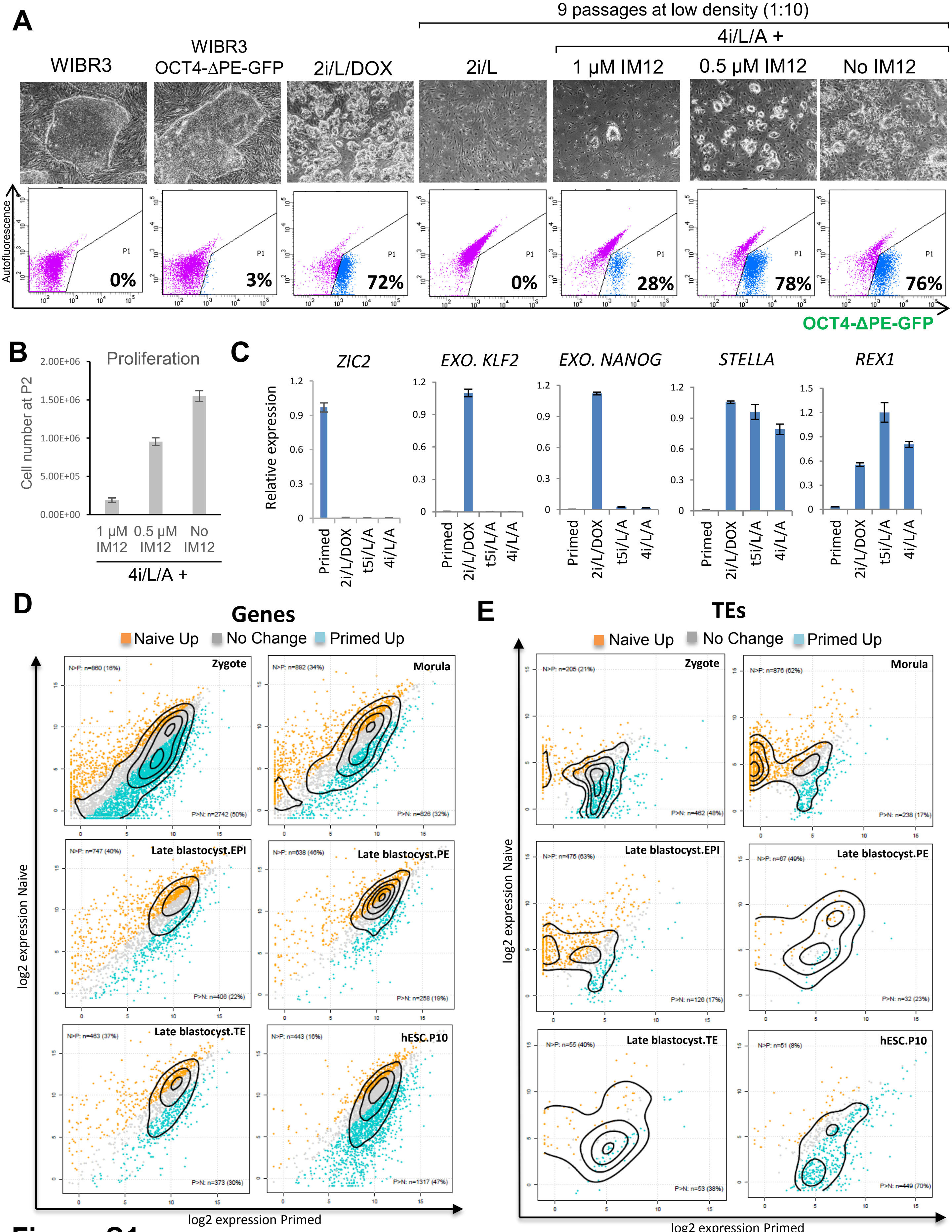
A
9 passages at low density (1:10)
4i/L/A +
WIBR3
WIBR3 OCT4-ΔPE-GFP
2i/L/DOX
2i/L
1 µM IM12
0.5 µM IM12
No IM12
Autofluorescence
P1
0%
3%
72%
0%
28%
78%
76%
OCT4-ΔPE-GFP
B
Proliferation
Cell number at P2
1 µM IM12
0.5 µM IM12
No IM12
4i/L/A +
C
ZIC2
EXO. KLF2
EXO. NANOG
STELLA
REX1
Relative expression
Primed
2i/L/DOX
t5i/L/A
4i/L/A
D
Genes
Naive Up
No Change
Primed Up
Zygote
Morula
Late blastocyst.EPI
Late blastocyst.PE
Late blastocyst.TE
hESC.P10
log2 expression Naive
log2 expression Primed
E
TEs
Naive Up
No Change
Primed Up
Zygote
Morula
Late blastocyst.EPI
Late blastocyst.PE
Late blastocyst.TE
hESC.P10
log2 expression Naive
log2 expression Primed

Figure S1

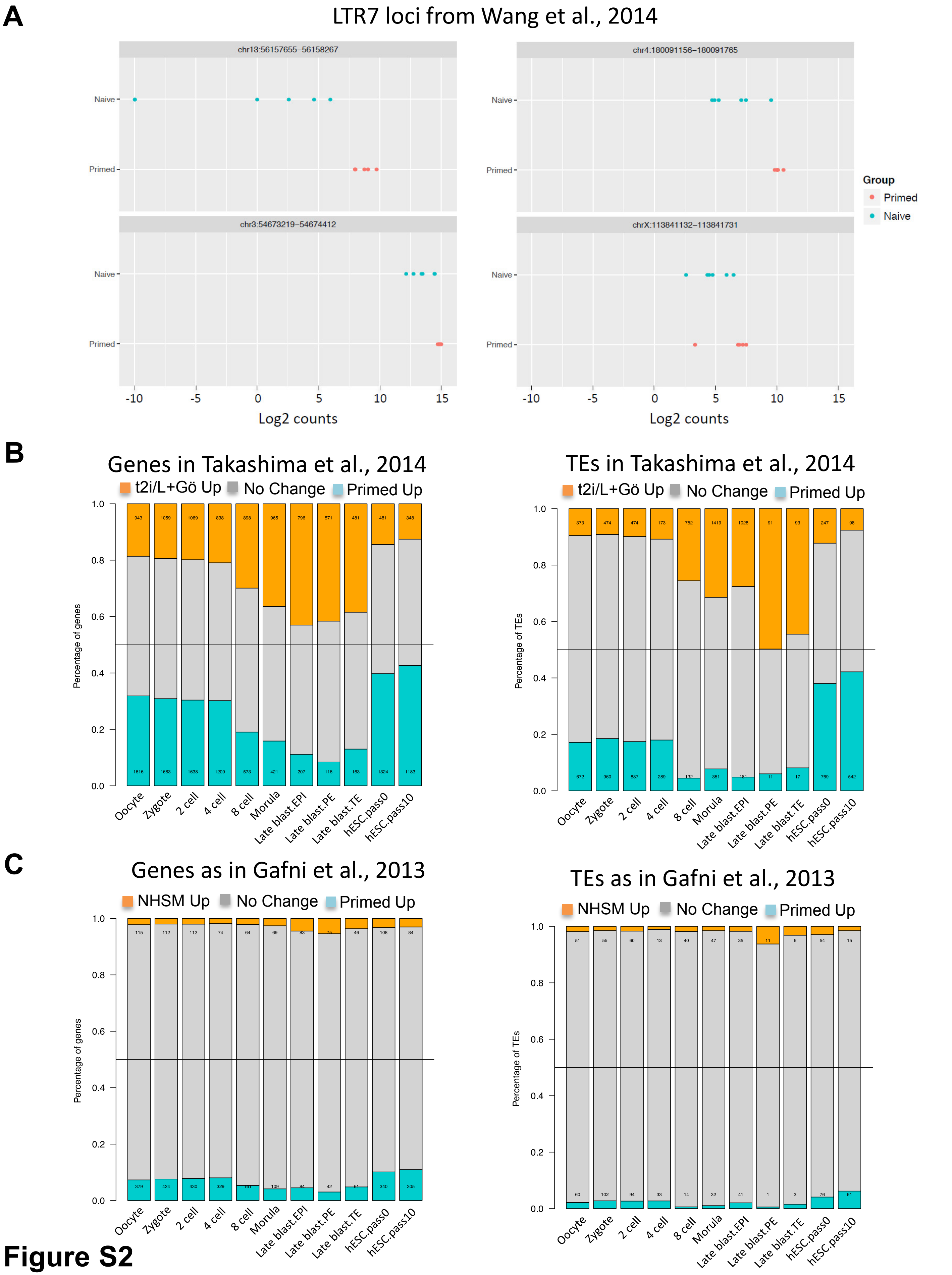
A
LTR7 loci from Wang et al., 2014
chr13:56157655–56158267
chr4:180091156–180091765
chr3:54673219–54674412
chrX:113841132–113841731
Naive
Primed
Group
Primed
Naive
Log2 counts
B
Genes in Takashima et al., 2014
TEs in Takashima et al., 2014
t2i/L+Gö Up
No Change
Primed Up
Percentage of genes
Percentage of TEs
Oocyte
Zygote
2 cell
4 cell
8 cell
Morula
Late blast.EPI
Late blast.PE
Late blast.TE
hESC.pass0
hESC.pass10
C
Genes as in Gafni et al., 2013
TEs as in Gafni et al., 2013
NHSM Up
No Change
Primed Up

Figure S2

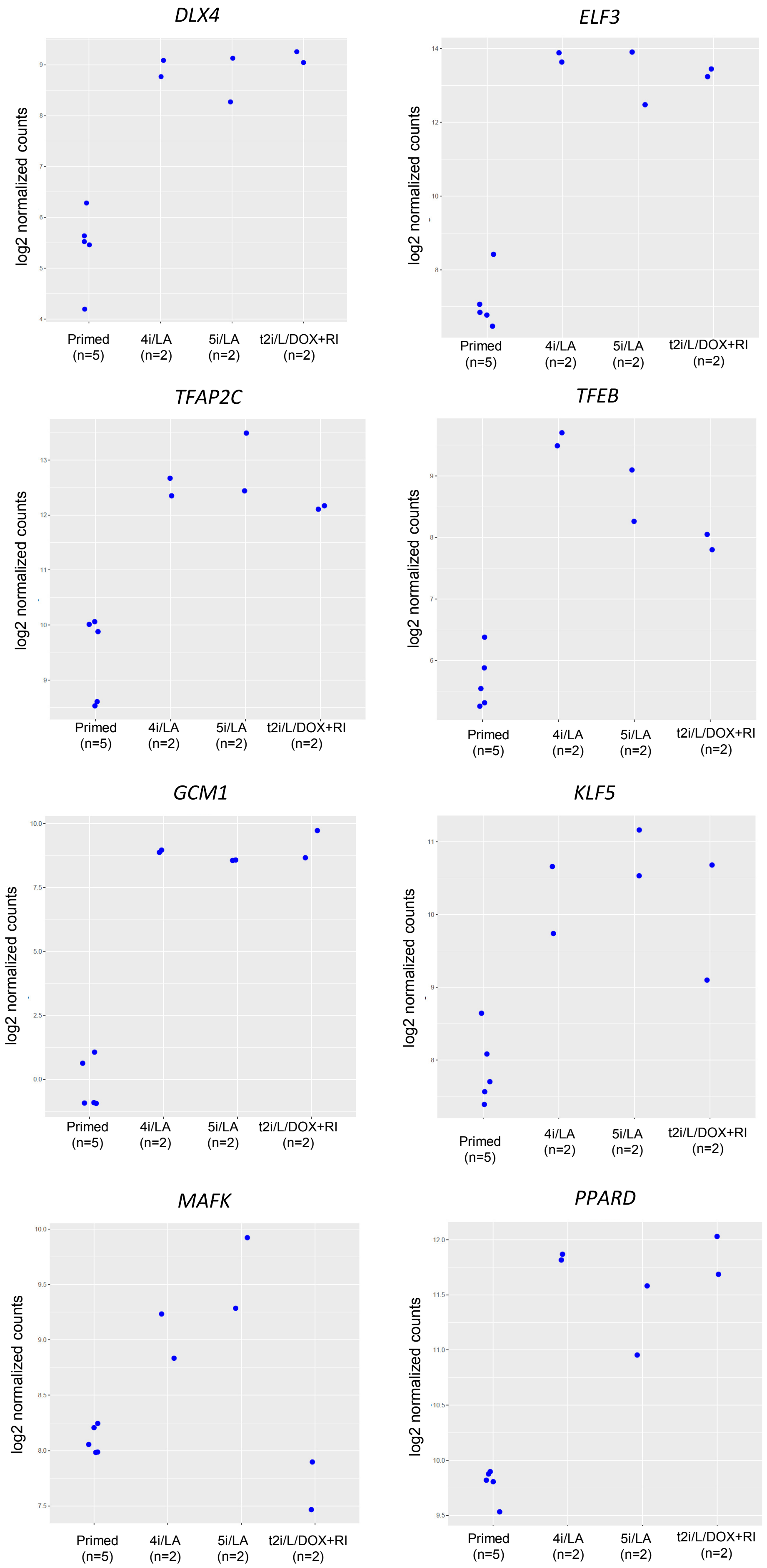
DLX4
ELF3
TFAP2C
TFEB
GCM1
KLF5
MAFK
PPARD
log2 normalized counts
Primed (n=5)
4i/LA (n=2)
5i/LA (n=2)
t2i/L/DOX+RI (n=2)

Figure S3

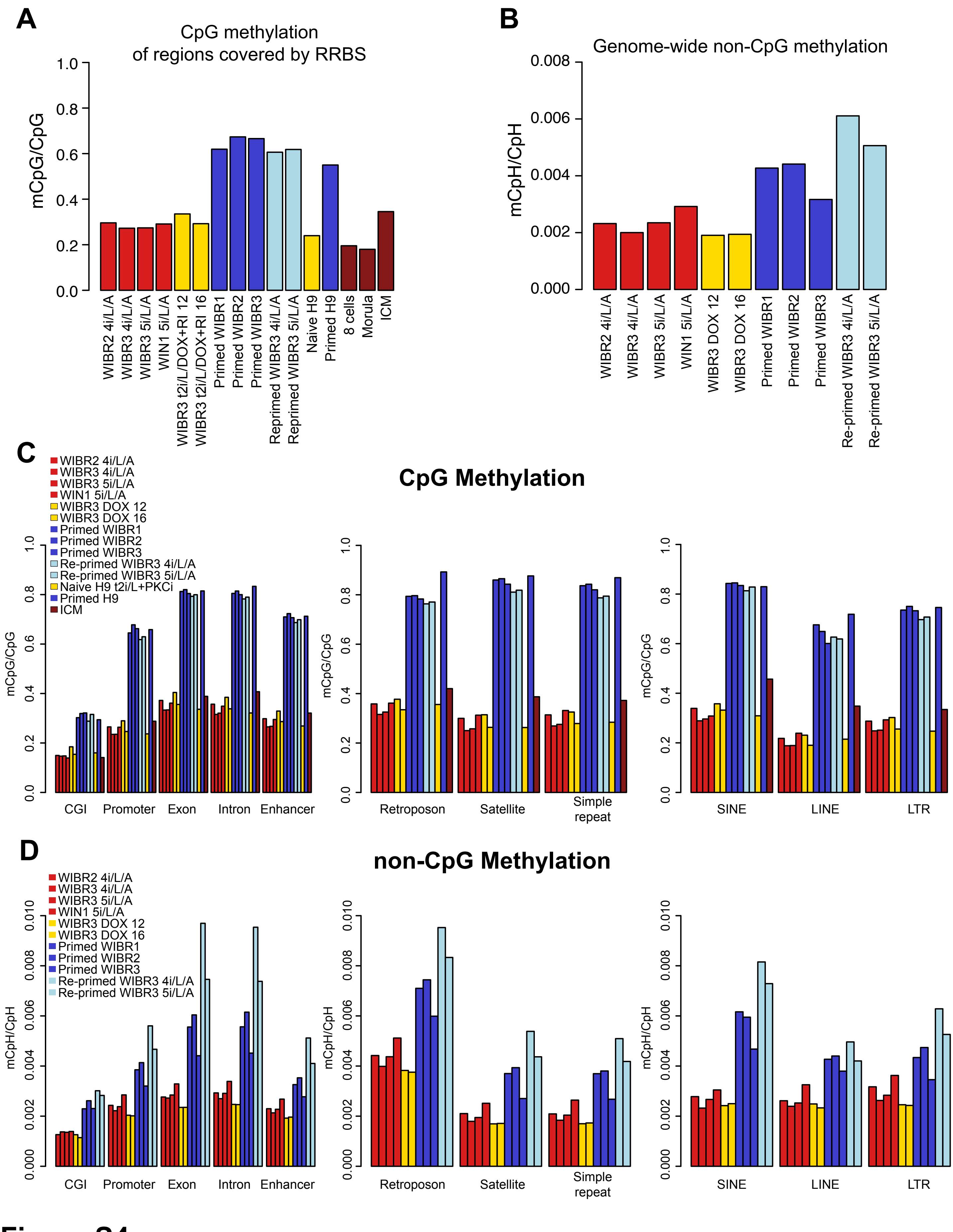
A
CpG methylation
of regions covered by RRBS
mCpG/CpG
1.0
0.8
0.6
0.4
0.2
0.0
WIBR2 4i/L/A
WIBR3 4i/L/A
WIBR3 5i/L/A
WIN1 5i/L/A
WIBR3 t2i/L/DOX+RI 12
WIBR3 t2i/L/DOX+RI 16
Primed WIBR1
Primed WIBR2
Primed WIBR3
Reprimed WIBR3 4i/L/A
Reprimed WIBR3 5i/L/A
Naive H9
Primed H9
8 cells
Morula
ICM
B
Genome-wide non-CpG methylation
mCpH/CpH
0.008
0.006
0.004
0.002
0.000
WIBR2 4i/L/A
WIBR3 4i/L/A
WIBR3 5i/L/A
WIN1 5i/L/A
WIBR3 DOX 12
WIBR3 DOX 16
Primed WIBR1
Primed WIBR2
Primed WIBR3
Re-primed WIBR3 4i/L/A
Re-primed WIBR3 5i/L/A
C
CpG Methylation
WIBR2 4i/L/A
WIBR3 4i/L/A
WIBR3 5i/L/A
WIN1 5i/L/A
WIBR3 DOX 12
WIBR3 DOX 16
Primed WIBR1
Primed WIBR2
Primed WIBR3
Re-primed WIBR3 4i/L/A
Re-primed WIBR3 5i/L/A
Naive H9 t2i/L+PKCi
Primed H9
ICM
mCpG/CpG
CGI
Promoter
Exon
Intron
Enhancer
Retroposon
Satellite
Simple repeat
SINE
LINE
LTR
D
non-CpG Methylation
WIBR2 4i/L/A
WIBR3 4i/L/A
WIBR3 5i/L/A
WIN1 5i/L/A
WIBR3 DOX 12
WIBR3 DOX 16
Primed WIBR1
Primed WIBR2
Primed WIBR3
Re-primed WIBR3 4i/L/A
Re-primed WIBR3 5i/L/A
mCpH/CpH
0.010
0.008
0.006
0.004
0.002
0.000
CGI
Promoter
Exon
Intron
Enhancer
Retroposon
Satellite
Simple repeat
SINE
LINE
LTR

Figure S4

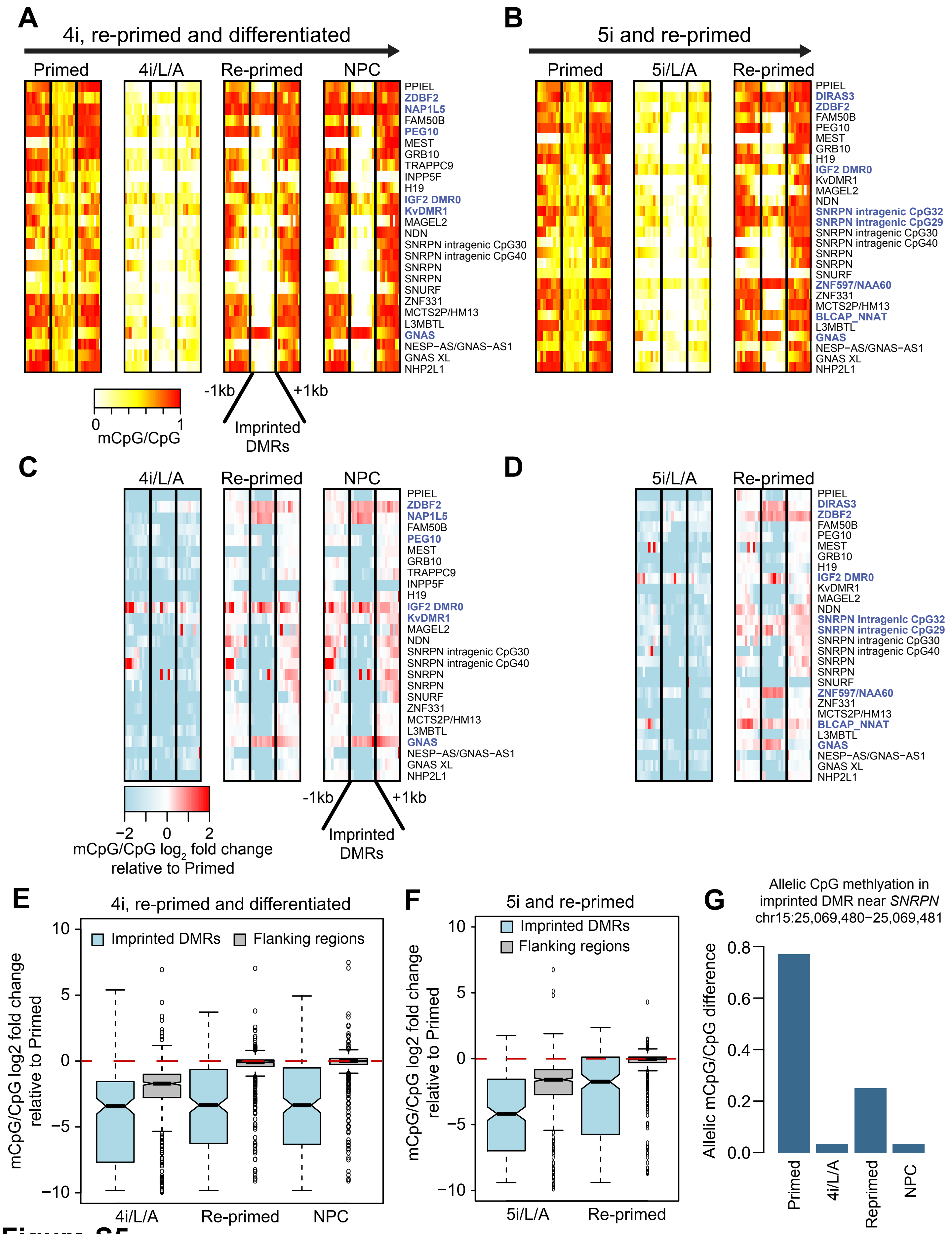
A
4i, re-primed and differentiated
Primed
4i/L/A
Re-primed
NPC
PPIEL
ZDBF2
NAP1L5
FAM50B
PEG10
MEST
GRB10
TRAPPC9
INPP5F
H19
IGF2 DMR0
KvDMR1
MAGEL2
NDN
SNRPN intragenic CpG30
SNRPN intragenic CpG40
SNRPN
SNRPN
SNURF
ZNF331
MCTS2P/HM13
L3MBTL
GNAS
NESP-AS/GNAS-AS1
GNAS XL
NHP2L1
0
1
mCpG/CpG
-1kb
+1kb
Imprinted DMRs
B
5i and re-primed
Primed
5i/L/A
Re-primed
PPIEL
DIRAS3
ZDBF2
FAM50B
PEG10
MEST
GRB10
H19
IGF2 DMR0
KvDMR1
MAGEL2
NDN
SNRPN intragenic CpG32
SNRPN intragenic CpG29
SNRPN intragenic CpG30
SNRPN intragenic CpG40
SNRPN
SNRPN
SNURF
ZNF597/NAA60
ZNF331
MCTS2P/HM13
BLCAP_NNAT
L3MBTL
GNAS
NESP-AS/GNAS-AS1
GNAS XL
NHP2L1
C
4i/L/A
Re-primed
NPC
−2
0
2
mCpG/CpG log2 fold change relative to Primed
-1kb
+1kb
Imprinted DMRs
D
5i/L/A
Re-primed
E
4i, re-primed and differentiated
Imprinted DMRs
Flanking regions
mCpG/CpG log2 fold change relative to Primed
10
5
0
−5
−10
4i/L/A
Re-primed
NPC
F
5i and re-primed
Imprinted DMRs
Flanking regions
5i/L/A
Re-primed
G
Allelic CpG methlyation in imprinted DMR near SNRPN chr15:25,069,480−25,069,481
Allelic mCpG/CpG difference
0.0
0.2
0.4
0.6
0.8
Primed
4i/L/A
Reprimed
NPC

Figure S5

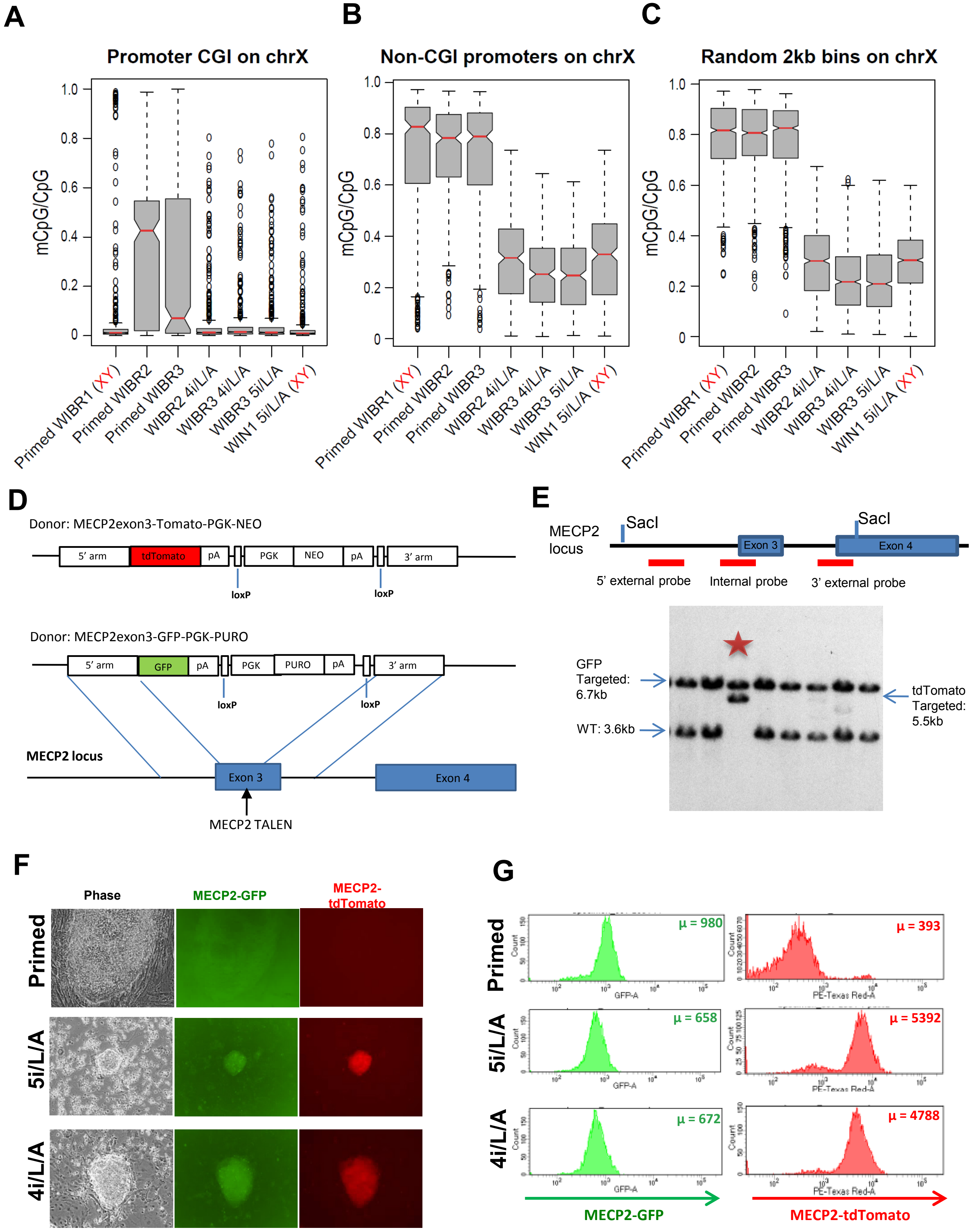
A
Promoter CGI on chrX
B
Non-CGI promoters on chrX
C
Random 2kb bins on chrX
mCpG/CpG
1.0
0.8
0.6
0.4
0.2
0
Primed WIBR1 (XY)
Primed WIBR2
Primed WIBR3
WIBR2 4i/L/A
WIBR3 4i/L/A
WIBR3 5i/L/A
WIN1 5i/L/A (XY)
D
Donor: MECP2exon3-Tomato-PGK-NEO
5' arm
tdTomato
pA
PGK
NEO
pA
3' arm
loxP
Donor: MECP2exon3-GFP-PGK-PURO
5' arm
GFP
pA
PGK
PURO
pA
3' arm
loxP
MECP2 locus
Exon 3
Exon 4
MECP2 TALEN
E
MECP2 locus
SacI
Exon 3
Exon 4
5' external probe
Internal probe
3' external probe
GFP Targeted: 6.7kb
WT: 3.6kb
tdTomato Targeted: 5.5kb
F
Phase
MECP2-GFP
MECP2-tdTomato
Primed
5i/L/A
4i/L/A
G
Primed
5i/L/A
4i/L/A
μ = 980
μ = 393
μ = 658
μ = 5392
μ = 672
μ = 4788
Count
GFP-A
PE-Texas Red-A
MECP2-GFP
MECP2-tdTomato

Figure S6

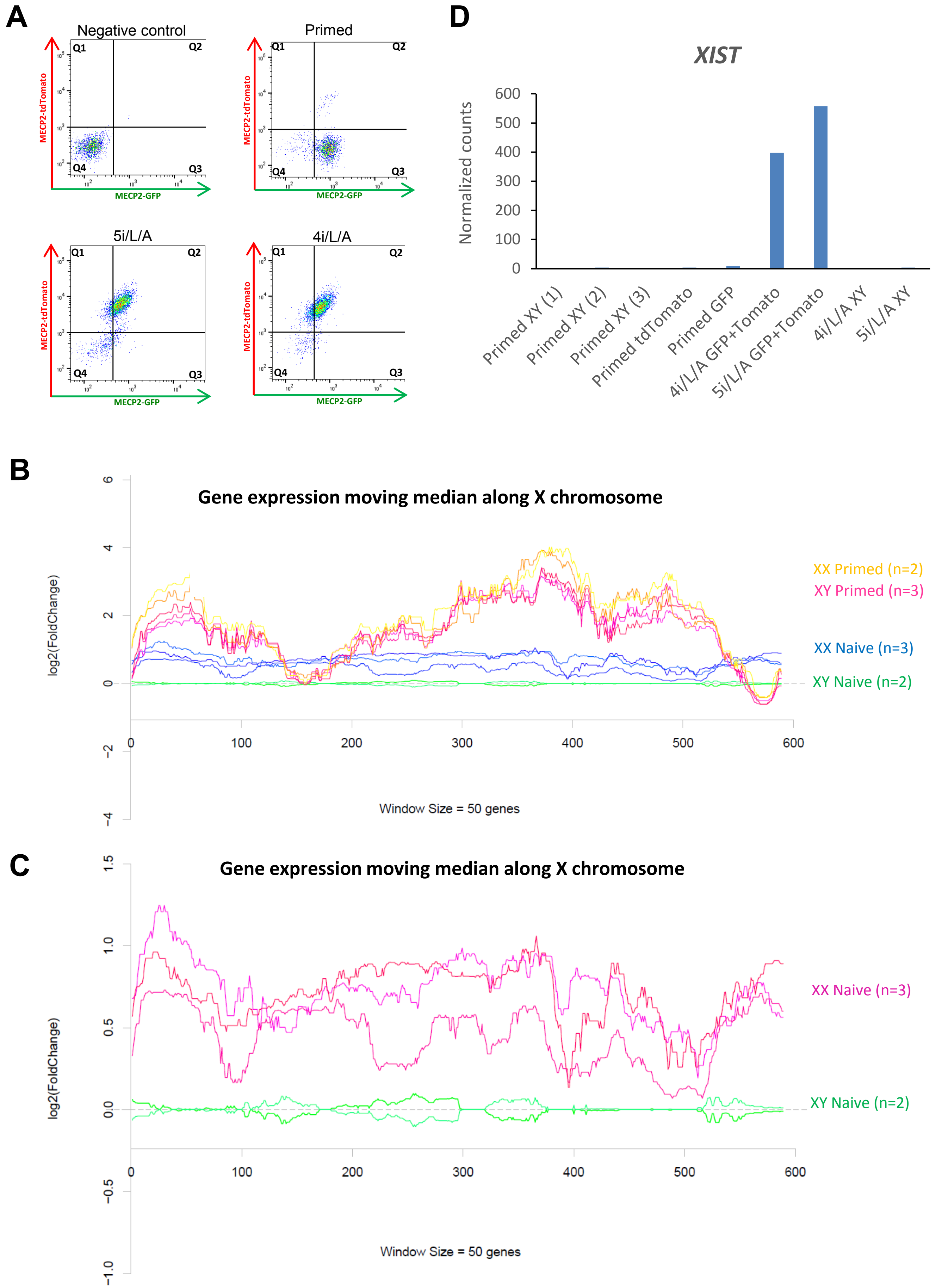
A
Negative control
Primed
5i/L/A
4i/L/A
Q1
Q2
Q3
Q4
MECP2-tdTomato
MECP2-GFP
D
XIST
Normalized counts
600
500
400
300
200
100
0
Primed XY (1)
Primed XY (2)
Primed XY (3)
Primed tdTomato
Primed GFP
4i/L/A GFP+Tomato
5i/L/A GFP+Tomato
4i/L/A XY
5i/L/A XY
B
Gene expression moving median along X chromosome
log2(FoldChange)
Window Size = 50 genes
XX Primed (n=2)
XY Primed (n=3)
XX Naive (n=3)
XY Naive (n=2)
C
Gene expression moving median along X chromosome
log2(FoldChange)
Window Size = 50 genes
XX Naive (n=3)
XY Naive (n=2)

Figure S7

V. Supplemental References

Bai, Q., Assou, S., Haouzi, D., Ramirez, J.M., Monzo, C., Becker, F., Gerbal-Chaloin, S., Hamamah, S., and De Vos, J. (2012). Dissecting the first transcriptional divergence during human embryonic development. Stem Cell Rev *8*, 150-162.

Balaton, B.P., Cotton, A.M., and Brown, C.J. (2015). Derivation of consensus inactivation status for X-linked genes from genome-wide studies. Biology of sex differences *6*, 35.

Benjamini, Y.a.H., Y. (1995). Controlling the false discovery rate: A practical and powerful approach to multiple testing. Journal of the Royal Statistical Society B.

Cohen, M.A., Itsykson, P., and Reubinoff, B.E. (2007). Neural differentiation of human ES cells. Curr Protoc Cell Biol *Chapter 23*, Unit 23 27.

Cohen, M.A., Wert, K.J., Goldmann, J., Markoulaki, S., Buganim, Y., Fu, D., and Jaenisch, R. (2016). Human neural crest cells contribute to coat pigmentation in interspecies chimeras after in utero injection into mouse embryos. Proceedings of the National Academy of Sciences of the United States of America.

Court, F., Tayama, C., Romanelli, V., Martin-Trujillo, A., Iglesias-Platas, I., Okamura, K., Sugahara, N., Simon, C., Moore, H., Harness, J.V.*, et al.* (2014). Genome-wide parent-of-origin DNA methylation analysis reveals the intricacies of human imprinting and suggests a germline methylation-independent mechanism of establishment. Genome research *24*, 554-569.

Dobin, A., Davis, C.A., Schlesinger, F., Drenkow, J., Zaleski, C., Jha, S., Batut, P., Chaisson, M., and Gingeras, T.R. (2013). STAR: ultrafast universal RNA-seq aligner. Bioinformatics *29*, 15-21.

Edgar, R., Domrachev, M., and Lash, A.E. (2002). Gene Expression Omnibus: NCBI gene expression and hybridization array data repository. Nucleic Acids Res *30*, 207-210.

Gafni, O., Weinberger, L., Mansour, A.A., Manor, Y.S., Chomsky, E., Ben-Yosef, D., Kalma, Y., Viukov, S., Maza, I., Zviran, A.*, et al.* (2013). Derivation of novel human ground state naive pluripotent stem cells. Nature *504*, 282-286.

Gentleman, R.C., Carey, V.J., Bates, D.M., Bolstad, B., Dettling, M., Dudoit, S., Ellis, B., Gautier, L., Ge, Y., Gentry, J., *et al.* (2004). Bioconductor: open software development for computational biology and bioinformatics. Genome Biol *5*, R80.

Guo, H., Zhu, P., Yan, L., Li, R., Hu, B., Lian, Y., Yan, J., Ren, X., Lin, S., Li, J., *et al.* (2014). The DNA methylation landscape of human early embryos. Nature *511*, 606-610.

Harrow, J., Frankish, A., Gonzalez, J.M., Tapanari, E., Diekhans, M., Kokocinski, F., Aken, B.L., Barrell, D., Zadissa, A., Searle, S., *et al.* (2012). GENCODE: the reference human genome annotation for The ENCODE Project. Genome research *22*, 1760-1774.

Langmead, B., and Salzberg, S.L. (2012). Fast gapped-read alignment with Bowtie 2. Nat Methods *9*, 357-359.

Law, C.W., Chen, Y., Shi, W., and Smyth, G.K. (2014). voom: Precision weights unlock linear model analysis tools for RNA-seq read counts. Genome Biol *15*, R29.

Lengner, C.J., Gimelbrant, A.A., Erwin, J.A., Cheng, A.W., Guenther, M.G., Welstead, G.G., Alagappan, R., Frampton, G.M., Xu, P., Muffat, J., *et al.* (2010). Derivation of pre-X inactivation human embryonic stem cells under physiological oxygen concentrations. Cell *141*, 872-883.

Li, H. (2011). A statistical framework for SNP calling, mutation discovery, association mapping and population genetical parameter estimation from sequencing data. Bioinformatics *27*, 2987-2993.

Maetzel, D., Sarkar, S., Wang, H., Abi-Mosleh, L., Xu, P., Cheng, A.W., Gao, Q., Mitalipova, M., and Jaenisch, R. (2014). Genetic and chemical correction of cholesterol accumulation and impaired autophagy in hepatic and neural cells derived from Niemann-Pick Type C patient-specific iPS cells. Stem cell reports *2*, 866-880.

Martin, M. (2011). Cutadapt removes adapter sequences from high-throughput sequencing reads. EMBnetjournal *17*.

Roberts, A., Trapnell, C., Donaghey, J., Rinn, J.L., and Pachter, L. (2011). Improving RNA-Seq expression estimates by correcting for fragment bias. Genome Biol *12*, R22.

Schultz, M.D., He, Y., Whitaker, J.W., Hariharan, M., Mukamel, E.A., Leung, D., Rajagopal, N., Nery, J.R., Urich, M.A., Chen, H., *et al.* (2015). Human body epigenome maps reveal noncanonical DNA methylation variation. Nature *523*, 212-216.

Schultz, M.D., Schmitz, R.J., and Ecker, J.R. (2012). 'Leveling' the playing field for analyses of single-base resolution DNA methylomes. Trends Genet *28*, 583-585.

Shen, L., Shao, N., Liu, X., and Nestler, E. (2014). ngs.plot: Quick mining and visualization of next-generation sequencing data by integrating genomic databases. BMC genomics *15*, 284.

Soldner, F., Laganiere, J., Cheng, A.W., Hockemeyer, D., Gao, Q., Alagappan, R., Khurana, V., Golbe, L.I., Myers, R.H., Lindquist, S., *et al.* (2011). Generation of isogenic pluripotent stem cells differing exclusively at two early onset Parkinson point mutations. Cell *146*, 318-331.

Takashima, Y., Guo, G., Loos, R., Nichols, J., Ficz, G., Krueger, F., Oxley, D., Santos, F., Clarke, J., Mansfield, W., *et al.* (2014). Resetting transcription factor control circuitry toward ground-state pluripotency in human. Cell *158*, 1254-1269.

Theunissen, T.W., Powell, B.E., Wang, H., Mitalipova, M., Faddah, D.A., Reddy, J., Fan, Z.P., Maetzel, D., Ganz, K., Shi, L., *et al.* (2014). Systematic identification of culture conditions for induction and maintenance of naive human pluripotency. Cell stem cell *15*, 471-487.

Wang, J., Xie, G., Singh, M., Ghanbarian, A.T., Rasko, T., Szvetnik, A., Cai, H., Besser, D., Prigione, A., Fuchs, N.V., *et al.* (2014). Primate-specific endogenous retrovirus-driven transcription defines naive-like stem cells. Nature *516*, 405-409.

Wang, T., Liu, J., Shen, L., Tonti-Filippini, J., Zhu, Y., Jia, H., Lister, R., Whitaker, J.W., Ecker, J.R., Millar, A.H., *et al.* (2013). STAR: an integrated solution to management and visualization of sequencing data. Bioinformatics *29*, 3204-3210.

Xie, W., Schultz, M.D., Lister, R., Hou, Z., Rajagopal, N., Ray, P., Whitaker, J.W., Tian, S., Hawkins, R.D., Leung, D., *et al.* (2013). Epigenomic analysis of multilineage differentiation of human embryonic stem cells. Cell *153*, 1134-1148.

Yan, L., Yang, M., Guo, H., Yang, L., Wu, J., Li, R., Liu, P., Lian, Y., Zheng, X., Yan, J., *et al.* (2013). Single-cell RNA-Seq profiling of human preimplantation embryos and embryonic stem cells. Nat Struct Mol Biol *20*, 1131-1139.
