# Supplementary material for: Molecular Criteria for Defining the Naive Human Pluripotent State
Source: Cell Stem Cell. 2016 Oct 6;19(4):502–15. doi: 10.1016/j.stem.2016.06.011 (PMC5065525; doi:10.1016/j.stem.2016.06.011)
Supplement: Document S2. Article plus Supplemental Information [file mmc6.pdf]

Cell Stem Cell

Molecular Criteria for Defining the Naive Human Pluripotent State

Graphical Abstract

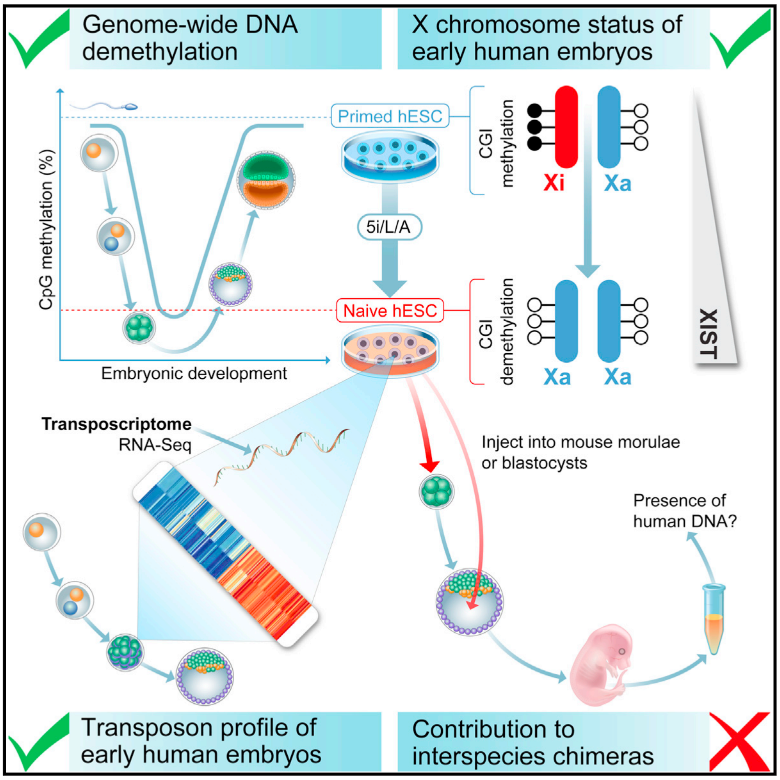

Authors

Thorold W. Theunissen, Marc Friedli, Yupeng He, ..., Didier Trono, Joseph R. Ecker, Rudolf Jaenisch

Correspondence

didier.trono@epfl.ch (D.T.),
ecker@salk.edu (J.R.E.),
jaenisch@wi.mit.edu (R.J.)

In Brief

Theunissen et al. use molecular criteria based on transposon expression, DNA methylation, and X chromosome status to compare naive human pluripotent cells to human preimplantation embryos. Current approaches yield cells that most closely resemble the morula/early blastocyst stage.

Highlights

- Naive human ESCs share a unique transposon signature with cleavage-stage embryos
- Global DNA demethylation in naive human ESCs is reversible except at imprinted loci
- The X chromosome status of naive human ESCs resembles the preimplantation embryo
- Naive human ESCs incorporate into the mouse morula or blastocyst very inefficiently

Accession Numbers

GSE75868

Theunissen et al., 2016, Cell Stem Cell *19*, 502–515
October 6, 2016 © 2016 The Authors. Published by Elsevier Inc.
http://dx.doi.org/10.1016/j.stem.2016.06.011

CellPress

Cell Stem Cell
Resource

Molecular Criteria for Defining the Naive Human Pluripotent State

Thorold W. Theunissen,[1,6] Marc Friedli,[2,6] Yupeng He,[3,5,6] Evarist Planet,[2] Ryan C. O'Neil,[3,5] Styliani Markoulaki,[1] Julien Pontis,[2] Haoyi Wang,[1,7] Alexandra Iouranova,[2] Michaël Imbeault,[2] Julien Duc,[2] Malkiel A. Cohen,[1] Katherine J. Wert,[1] Rosa Castanon,[3] Zhuzhu Zhang,[3] Yanmei Huang,[1] Joseph R. Nery,[3] Jesse Drotar,[1] Tenzin Lungjangwa,[1] Didier Trono,[2,*] Joseph R. Ecker,[3,*] and Rudolf Jaenisch[1,4,8,*]
[1]Whitehead Institute for Biomedical Research, Cambridge, MA 02142, USA
[2]School of Life Sciences, Ecole Polytechnique Fédérale de Lausanne (EPFL), 1015 Lausanne, Switzerland
[3]Genomic Analysis Laboratory and Howard Hughes Medical Institute, Salk Institute for Biological Studies, La Jolla, CA 92037, USA
[4]Department of Biology, Massachusetts Institute of Technology, Cambridge, MA 02142, USA
[5]Bioinformatics Program, University of California, San Diego, La Jolla, CA 92093, USA
[6]Co-first author
[7]Present address: State Key Laboratory of Stem Cell and Reproductive Biology, Institute of Zoology, Chinese Academy of Sciences, Beijing 100864, People's Republic of China
[8]Lead Contact
*Correspondence: didier.trono@epfl.ch (D.T.), ecker@salk.edu (J.R.E.), jaenisch@wi.mit.edu (R.J.)
http://dx.doi.org/10.1016/j.stem.2016.06.011

SUMMARY

Recent studies have aimed to convert cultured human pluripotent cells to a naive state, but it remains unclear to what extent the resulting cells recapitulate in vivo naive pluripotency. Here we propose a set of molecular criteria for evaluating the naive human pluripotent state by comparing it to the human embryo. We show that transcription of transposable elements provides a sensitive measure of the concordance between pluripotent stem cells and early human development. We also show that induction of the naive state is accompanied by genome-wide DNA hypomethylation, which is reversible except at imprinted genes, and that the X chromosome status resembles that of the human preimplantation embryo. However, we did not see efficient incorporation of naive human cells into mouse embryos. Overall, the different naive conditions we tested showed varied relationships to human embryonic states based on molecular criteria, providing a backdrop for future analysis of naive human pluripotency.

INTRODUCTION

Pluripotent stem cells from mouse and human have distinct morphologies, signaling requirements and epigenetic configurations. It has been proposed that mouse embryonic stem cells (ESCs) and induced pluripotent stem cells (iPSCs) represent a naive state of pluripotency corresponding to the inner cell mass (ICM), whereas human ESCs/iPSCs correspond to a more advanced, or "primed," state of pluripotency found in the postimplantation epiblast (Nichols and Smith, 2009). A number of protocols have been described for inducing naive features in human ESCs, mostly testing candidates (Chan et al., 2013; Gafni et al., 2013; Takashima et al., 2014; Ware et al., 2014). We took a systematic approach by screening a kinase inhibitor library for the ability to maintain activity of the distal enhancer of *OCT4* (Theunissen et al., 2014). Through iterative screening, we identified a combination of five kinase inhibitors that, together with LIF and activin A (5i/L/A), enabled the conversion of pre-existing human ESCs to the naive state in the absence of transgenes. An independent analysis concluded that naive human cells generated with this method or in titrated 2i/L medium supplemented with a protein kinase C (PKC) inhibitor (Takashima et al., 2014) displayed the closest transcriptional similarity to both the human blastocyst and mouse ESCs in 2i/L (Huang et al., 2014).

It has been challenging to define the naive state of pluripotency in humans, particularly in view of the expanding number of protocols for deriving putative naive human cells (De Los Angeles et al., 2015; Wu and Izpisua Belmonte, 2015). While robust standards such as chimera formation can be used to definitively define naive pluripotency in mouse ESCs, such assays are not available in the human system, necessitating the establishment of alternative criteria. We and others previously assessed naive human cells according to features of naive pluripotency observed in mouse, such as *OCT4* distal enhancer activity, expression of naive-specific transcripts, and reduced bivalent domains (Chan et al., 2013; Gafni et al., 2013; Takashima et al., 2014; Theunissen et al., 2014; Ware et al., 2014). However, a comprehensive examination of the extent to which naive cells resemble early human embryos has not been described so far. Here we propose rigorous criteria for evaluating naive human pluripotency based on emerging insights into human preimplantation development (Guo et al., 2014; Okamoto et al., 2011; Petropoulos et al., 2016; Yan et al., 2013). A priori, the expectation based on parallels with naive mouse ESCs would be that naive human ESCs would be most closely related to the ICM of the blastocyst. We show using a range of molecular assays that naive human cells in 5i/L/A and other conditions acquire key features of corresponding pluripotent cells in vivo but fail to recapitulate the embryonic context entirely. Our results present a framework for future analysis and improvement of naive culture conditions.

 © 2016 The Authors. Published by Elsevier Inc.
This is an open access article under the CC BY license (http://creativecommons.org/licenses/by/4.0/).

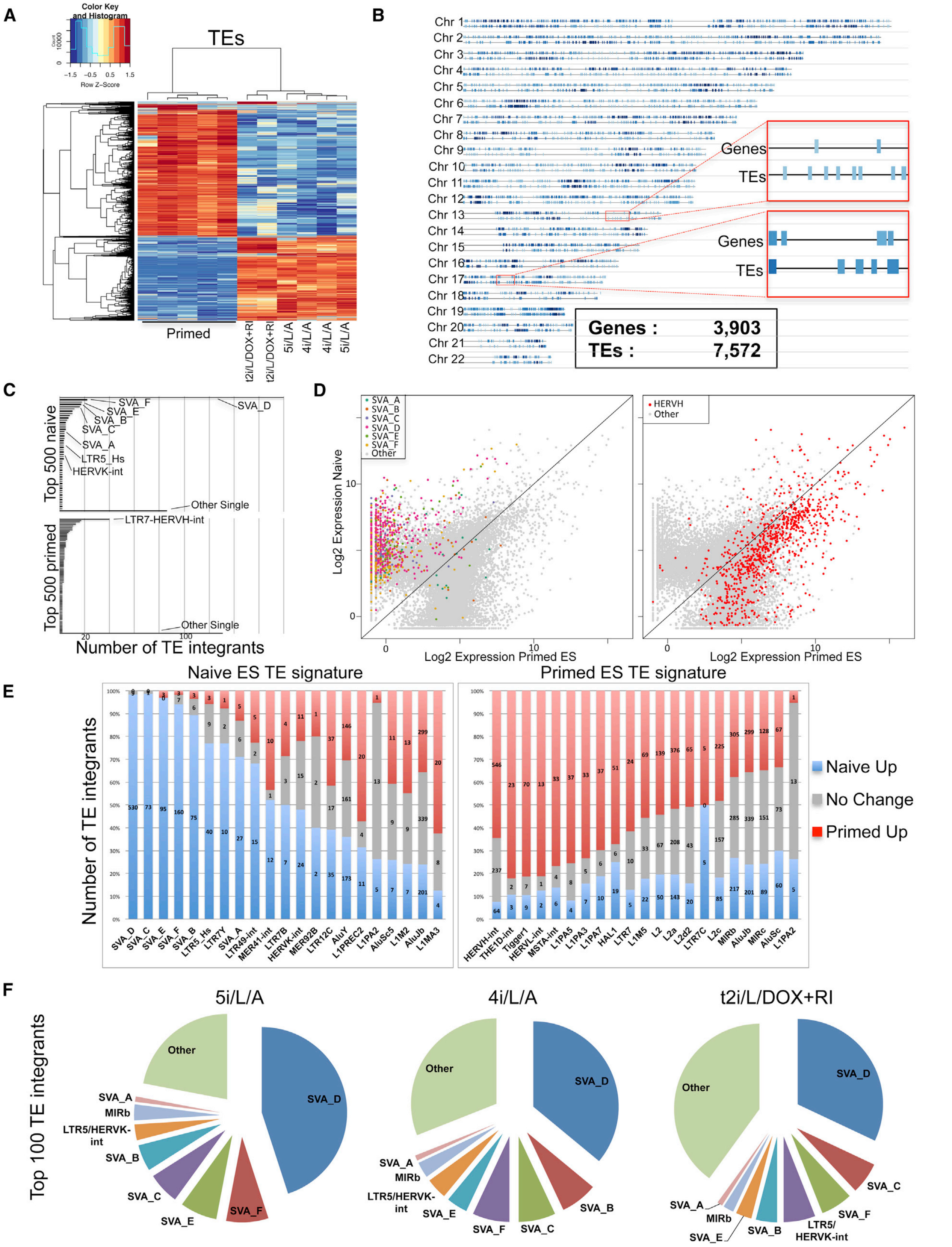

(legend on next page)

RESULTS

Naive Human ESCs Display a Transposon Transcription Signature of Cleavage-Stage Embryos

Transposable elements (TEs) are mobile genetic entities that constitute over half the human genome, and whose sequential expression during embryonic development is tightly regulated by species-specific *trans*-acting factors (Friedli and Trono, 2015). We recently generated an updated census of the transposcriptome, or sum of TE-derived transcripts in a cell, which provides a high-density barcode that can be used to characterize cellular identity. Here we explored whether TE expression profiling can be used to distinguish naive and primed human ESCs, and measure the correspondence of pluripotent stem cells to distinct stages of human embryogenesis.

TE-derived transcripts were analyzed using an improved RNA sequencing (RNA-seq) methodology in conventional (primed) human ESCs and three conditions for naive human pluripotency: (1) ectopic expression of doxycycline (DOX)-inducible KLF2 and NANOG transgenes (Takashima et al., 2014; Theunissen et al., 2014); (2) our previously described 5i/L/A conditions (Theunissen et al., 2014); and (3) a modified version of 5i/L/A in which the GSK3 inhibitor is removed (4i/L/A), which confers enhanced proliferation but results in a flatter colony morphology (Figures S1A–S1C, available online). The top 10,000 TEs with largest SD perfectly separated naive and primed cells (Figure 1A). Taking a 2-fold cutoff and $p < 0.05$, 16,311 loci (Table S1) were differentially expressed between naive and primed cells, with a slight imbalance (38.8% versus 61.2%) in favor of the latter. Differentially expressed TEs provided much denser coverage of human chromosomes than genes, as expected from their relative abundance in the genome (>four million TE integrants versus ~25,000 genes), producing a high-density barcode for the state of human ESCs (Figure 1B).

We asked which TEs were overexpressed in naive cells and found that members of the SINE-VNTR-*Alu* (SVA) family of TEs, in particular the SVA-D subgroup, were transcribed almost exclusively in the naive state. Of the top ten integrants with the highest naive-to-primed difference, four were SVA-Ds (Table S2), and of the top 500, 258 were SVAs, with a strong predominance of SVA-Ds (181) (Figures 1C and 1D). Of 539 SVA-Ds that produced RNA-seq reads above detection threshold, 530 were differentially expressed (98.3%), all of them at higher levels in the naive state (Figure 1E). SVAs are an evolutionarily young (hominid-specific) class of retroelements, and active retrotransposition of some SVA integrants has been reported in the human genome (Hancks and Kazazian, 2010). We also found the HERVK-associated LTR, LTR5-Hs, to be more readily transcribed in naive cells (52 loci expressed, 82.7% differentially, 93.0% of them more strongly in naive cells).

Surprisingly, none of the top 100 naive-expressed TEs belonged to the LTR7-HERVH family (only two LTR7-HERVHs and two unmerged LTR7s in the top 500), contrary to the recent suggestion that this endogenous retrovirus and its promoters are specific to naive-like cells (Wang et al., 2014) (Figure S2A). Instead, LTR7-HERVH integrants were collectively more expressed in primed cells, with 40 of them among the top 500 TEs of this category (Figures 1C and 1D). Furthermore, of 847 HERVH-int elements for which transcription was detected in at least one of our ESC samples, 610 (72.0%) were differentially expressed, with 546 (89.5%) higher in primed cells (Figure 1E). Globally, the transcription of SVAs (SVA-A to a lesser extent) and of HERVH-int was thus highly polarized, with a majority of integrants from these two TE subfamilies displaying preferential expression in naive and primed states, respectively.

The three conditions for naive human cells that we examined induced comparable upregulation of SVAs (mainly SVA-D) and LTR5_Hs integrants (Figure 1F). The transposcriptomes of naive cells in 5i/L/A and 4i/L/A were especially similar (Figure 1A), reflecting the close alignment of their global gene expression profiles (Figure 2A). Transgene-dependent naive cells were enriched in SVAs and LTR5_Hs expression (Figure 1F), but formed a discrete cluster in terms of TE expression (Figure 1A) or gene expression (Figure 2A). Principal component analysis (PCA) based on differentially expressed TEs uncovered greater variability between biological replicates than gene expression-based PCA (Figures 2A and 2B), highlighting the ability of TEs to uncover subtle differences between cell states.

Given the enhanced sensitivity of TE profiling, we hypothesized that the transposcriptome might provide a precise measure of the degree to which naive and primed ESCs resemble pluripotent cells in vivo. We therefore matched TE-derived RNA-seq data from naive and primed human ESCs with previous single-cell analyses of early human embryos containing an appropriate read length for transposcriptome analysis (the source loci of many TE-derived transcripts cannot be properly

Figure 1. TEs Accurately Discriminate Naive and Primed Human ESCs

(A) Heatmap of RNA-seq expression data from primed human ESCs and naive human ESCs derived in 5i/L/A-, 4i/L/A-, and transgene (KLF2/NANOG)-dependent naive cells maintained in t2i/L/DOX+RI. Data shown include 10,000 TEs with the highest SD between the samples.

(B) Naive/primed differentially expressed TEs (bottom) or genes (top) are depicted along human chromosomes. A cutoff fold change of 10× ($p < 0.05$) between naive and primed states was used to visualize the distribution of the most significantly differentially expressed TEs and genes. Note that TEs provide a much denser coverage of the genome.

(C) The top 500 TE integrants with the highest fold change between primed and naive ESCs are heavily enriched for HERVH-int-LTR7 (primed) or SVA and LTR5_Hs integrants (naive).

(D) Dot plot of all expressed TE integrants in naive versus primed ESCs. SVA-D, SVA-F, SVA-E, SVA-C, and SVA-B integrants are broadly expressed in naive ESCs (left), while SVA-A integrants are more evenly distributed. Similarly, HERVH-ints were by and large overexpressed in primed ESCs (right)

(E) TE signatures of naive or primed ESCs. The most heavily primed or naive-biased TE families are represented as columns split into three segments: naive-overexpressed integrants (2-fold cutoff, p adj. < 0.05), primed-overexpressed integrants, and no change (integrants with expression ratio not contained in the above cutoff).

(F) Pie charts comparing the top 100 TEs with highest fold change for different naive media (5i/L/A, 4i/L/A, and t2i/L/DOX+RI) compared to primed ESCs. The overall distribution is similar, with SVA-D representing about one-third of the top 100 TEs.

See also Figures S1 and S2.

CellPress

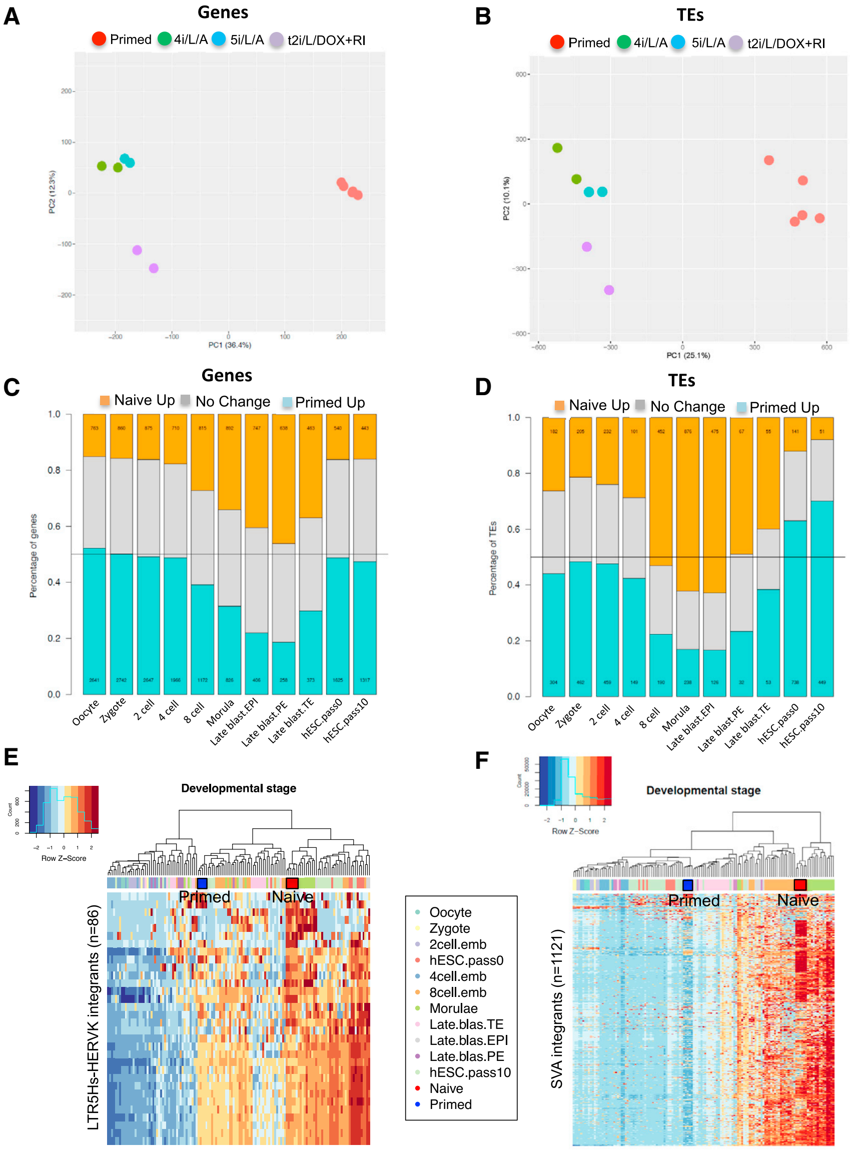

Figure 2. Naive Human ESCs Have a Transposon Transcription Signature of the Human Preimplantation Embryo

(A and B) Principal component analysis (PCA) of primed or naive human ESCs derived in 5i/L/A-, 4i/L/A-, and transgene (KLF2/NANOG)-dependent naive cells maintained in t2i/L/DOX+RI based on the differential expression of genes (A) or transposable elements (B).

(C and D) Correspondence between gene expression (C) or TE expression (D) in naive/primed ESCs and single-cell human embryonic stages (Yan et al., 2013). For every stage of human embryonic development, a statistical test was performed to find the genes (or TEs) that have a different expression level compared to the other stages. The proportions of developmental stage-specific genes (or TEs) that are upregulated ($p < 0.05$, 2-fold change) in naive or primed cells are indicated in orange and blue, respectively, while genes (or TEs) that did not change expression are indicated in gray. The naive samples include all three conditions of naive cells that we examined in the PCA analyses (i.e., 5i/L/A, 4i/L/A, and t2i/L/DOX+RI). See Supplemental Experimental Procedures for details of the analysis.

(E and F) Heatmaps of RNA-seq data from naive/primed ESCs and from single-cell human embryonic stages (Yan et al., 2013). Clustering is performed with all LTR5_Hs/HERVK elements with expression data (n = 86) (E) or all SVA elements with expression data (n = 1121) (F). The naive samples shown in red include all three conditions of naive cells that we examined in the PCA analyses (i.e., 5i/L/A, 4i/L/A, and t2i/L/DOX+RI).

See also Figures S1–S3.

mapped if reads are below 100 bp) (Yan et al., 2013). Naive cells displayed the most significant overlap with the human morula and epiblast stages: 62% (n = 876) of morula-enriched TEs and 63% (n = 475) of epiblast-enriched TEs were upregulated in naive compared to primed human ESCs ($p < 0.05$, 2-fold change) (Figures 2D and S1E). This suggests that 5i/L/A and 4i/L/A induce a pluripotent state that corresponds most closely to a late morula or early blastocyst identity. Based on gene expression, naive cells were most closely aligned with the late blastocyst (Figures 2C and S1D), but TE profiling revealed a greater contrast between naive and primed states (Figures 2D and S1E). Naive human cells generated with inducible KLF2 and NANOG transgenes in t2i/L+PKCi (Takashima et al., 2014) presented largely similar gene and TE expression profiles, but upregulation of morula-specific TEs was more pronounced in 5i/L/A and 4i/L/A (Figure S2B). In contrast, the NHSM protocol (Gafni et al., 2013) did not induce blastocyst-specific genes or TEs to a significant extent in our hands (Figure S2C), though the original dataset (Gafni et al., 2013) cannot be analyzed in a comparable way. Primed cells shared gene and TE expression with early-passage human ESCs (post-natal day 0 [P0] and P10) and very early (one- to four-cell-stage) human cleavage embryos. The overlap in gene expression between primed cells and one- to four-cell-stage embryos was noted before and attributed to upregulation of mitotic regulators that are expressed before embryonic genome activation occurs (Huang et al., 2014). Thus, we conclude that the transposcriptome provides a robust sensor of the correspondence between pluripotent states in vitro and the human preimplantation embryo.

Epiblast cells from late-stage mouse blastocysts have been identified as the closest in vivo counterpart of naive mouse ESCs based on their gene expression profile and capacity as a source for ESC derivation (Boroviak et al., 2014). Hence, the finding that naive human cells display comparable induction of morula and epiblast-associated TEs was unexpected. The overlap between the transposcriptomes of naive human cells and morula-stage embryos was driven largely by the pronounced upregulation of SVA and LTR5_Hs integrants: unsupervised clustering based on the expression of these two TE families revealed a strong convergence between naive cells and morulae, whereas primed cells clustered most closely with previously analyzed

human ESCs at passage 10 (Figures 2E and 2F). This transposon transcription signature points to a slightly earlier developmental stage as being the equivalent of naive human cells than would necessarily be predicted. However, as a result it is possible that these naive human cells might also be able to differentiate into the extra-embryonic lineages. Indeed, we observed significant upregulation in the naive state of 8 out of 16 transcription factors reported to be overexpressed in trophectoderm and placenta compared to conventional human ESCs (Bai et al., 2012) (Figure S3). These observations suggest that naive human cells could provide a convenient model system to study the initiation of trophectoderm differentiation.

Control of Transposon Transcription in Naive and Primed Human ESCs

The group-like behavior of SVA-D and LTR7/HERVH integrants suggested that these TEs might be collectively subjected to the influence of *trans*-regulators capable of controlling multiple members of a same family. The KRAB-ZFP/KAP1 system is central to the repression of a broad range of TEs during early embryogenesis (Matsui et al., 2010; Rowe et al., 2010; Turelli et al., 2014), and we recently determined that a vast majority of human KRAB-ZFPs have TEs as their preferential genomic targets (unpublished data). Correspondingly, most KRAB-ZFP genes displayed highly state-specific modes of expression in human ESCs, being markedly more expressed either in naive or in primed cells (Figure 3A). Levels of *ZNF534*, a KRAB-ZFP we identified as responsible for recognizing HERVH (unpublished data), were significantly higher in naive than primed ESCs (Figure 3B).

Among the top 100 TEs preferentially expressed in primed ESCs, twelve were enriched for KAP1 in naive cells and none in primed cells, as determined by chromatin immunoprecipitation followed by deep sequencing (ChIP-seq). As expected from the transposcriptome data, HERVH integrants were much less frequently KAP1 bound in primed than naive ESCs (Figure 3C). Interestingly, KAP1 binding extended further 3′ into LTR7-HERVH-int merged elements in naive ESCs, suggesting an additional specific KAP1 binding site within HERVH-int in this context (Figure 3C). Consistent with their repression, we detected H3K9me3 enrichment at many HERVH elements in naïve, but not primed, ESCs (Figures 3D and 3E). No overall difference in H3K4me3 was observed at these elements, consistent with a poised state in naive ESCs (Figure 3E). As expected from their naive-restricted expression, SVA elements exhibited reduced H3K9me3 and increased H3K4me3 enrichment in naive ESCs (Figures 3D and 3E), despite slightly increased KAP1 deposition in this setting (Figure 3C). This strongly suggests that on these elements, as previously observed at other loci (Iyengar and Farnham, 2011; Singh et al., 2015), KAP1 does not act as a co-repressor. However, members of the SVA-A subfamily, the only one not to display strong naive-biased expression, bore H3K9me3 in both primed and naive ESCs (Figure 3E). Thus, LTR7-HERVH and SVAs show dichotomous transcriptional and epigenetic marks specific to primed and naive states, respectively.

TEs can influence transcription through a variety of mechanisms (Friedli and Trono, 2015). Supportive of promoter or proximal enhancer effects, we found a significant correlation between expression of genes and their closest TEs. This correlation was independent of the respective orientations of the two components of a TE-gene pair (Figure 3F) and tailed off as their distance increased (Figures 3G and 3H). Interestingly, the effect on gene expression appeared more local for LTR-containing TEs with expression correlation for TEs located within the gene boundaries or in near vicinity, consistent with promoter activity (Figure 3G). SVAs, on the other hand, did not influence gene expression when located within genes and appeared to act at longer distances from genes, suggesting enhancer effects (Figure 3H).

Naive Induction Is Accompanied by a Genome-Wide Depletion in DNA Methylation that Is Reversible upon Differentiation except at Imprinted DMRs

Human preimplantation development is marked by a global reduction in DNA methylation (Guo et al., 2014). We generated high-coverage base-resolution methylomes (>30× coverage) of naive and primed human ESCs using MethylC-seq (Lister et al., 2009; Urich et al., 2015) (Table S3). All conditions examined for naive human cells (5i/L/A, 4i/L/A, and DOX-inducible expression of KLF2 and NANOG transgenes) were characterized by global hypomethylation in both CpG (26.9%–33.2%) and non-CpG contexts (0.19%–0.29%) and compared to their primed and re-primed counterparts (CpG, 75.2%–81.0%; non-CpG, 0.32%–0.60%) (Figures 4A, 4B, S4A, and S4B; Table S3). Similarly, widespread hypomethylation was observed in human cleavage-stage embryos (Guo et al., 2014) and naive human ESCs maintained in t2i/L+PKCi (Takashima et al., 2014) (Figures 4B and S4A; Table S3). Such CpG and non-CpG demethylation in naive cells was not restricted to specific genomic features but occurred at CpG islands (CGIs), exons, introns, promoters, and enhancers (Figures S4C and S4D).

CpG methylation is a major mechanism controlling TE expression during human embryonic development (Guo et al., 2014). We therefore investigated the correlation between CpG methylation and TE expression in naive and primed human ESCs. Along with genome-wide demethylation, TEs in naive cells were hypomethylated relative to primed cells (Figures S4C and S4D). Consequently, directly comparing the methylation levels of TEs in naive and primed cells was uninformative. Instead, we compared the methylation levels of overexpressed TEs and non-overexpressed copies from the same repeat family (as control). In naive cells, we focused on the most overexpressed family, SVA. Notwithstanding the overall low methylation in naive ESCs, overexpressed TE integrants tended to be further hypomethylated compared to non-overexpressed copies in naive cells (Figure 4C; $p < 0.05$, Mann-Whitney test). Likewise, for the primed cells we focused on the most overexpressed groups, HERVH-int and LTR7 sub families (LTR7, LTR7Y, LTR7B, and LTR7C), and found these to be hypomethylated compared to their non-primed-overexpressed counterparts (Figures 4D and 4E; $p < 0.05$, Mann-Whitney test).

Differentially methylated regions (DMRs) within imprinted regions are protected from both active and passive demethylation during preimplantation development to safeguard the transmission of parent-of-origin-specific epigenetic marks (Lee et al., 2014). We examined whether imprinted DMRs in human ESCs (Court et al., 2014) were resistant to methylation erasure upon conversion to the naive state. While imprinted DMRs had an intermediate level of CpG methylation (0.3–0.7) in primed cells, methylation in these regions was dramatically reduced in naive lines, suggesting demethylation of both alleles (Figures 5A

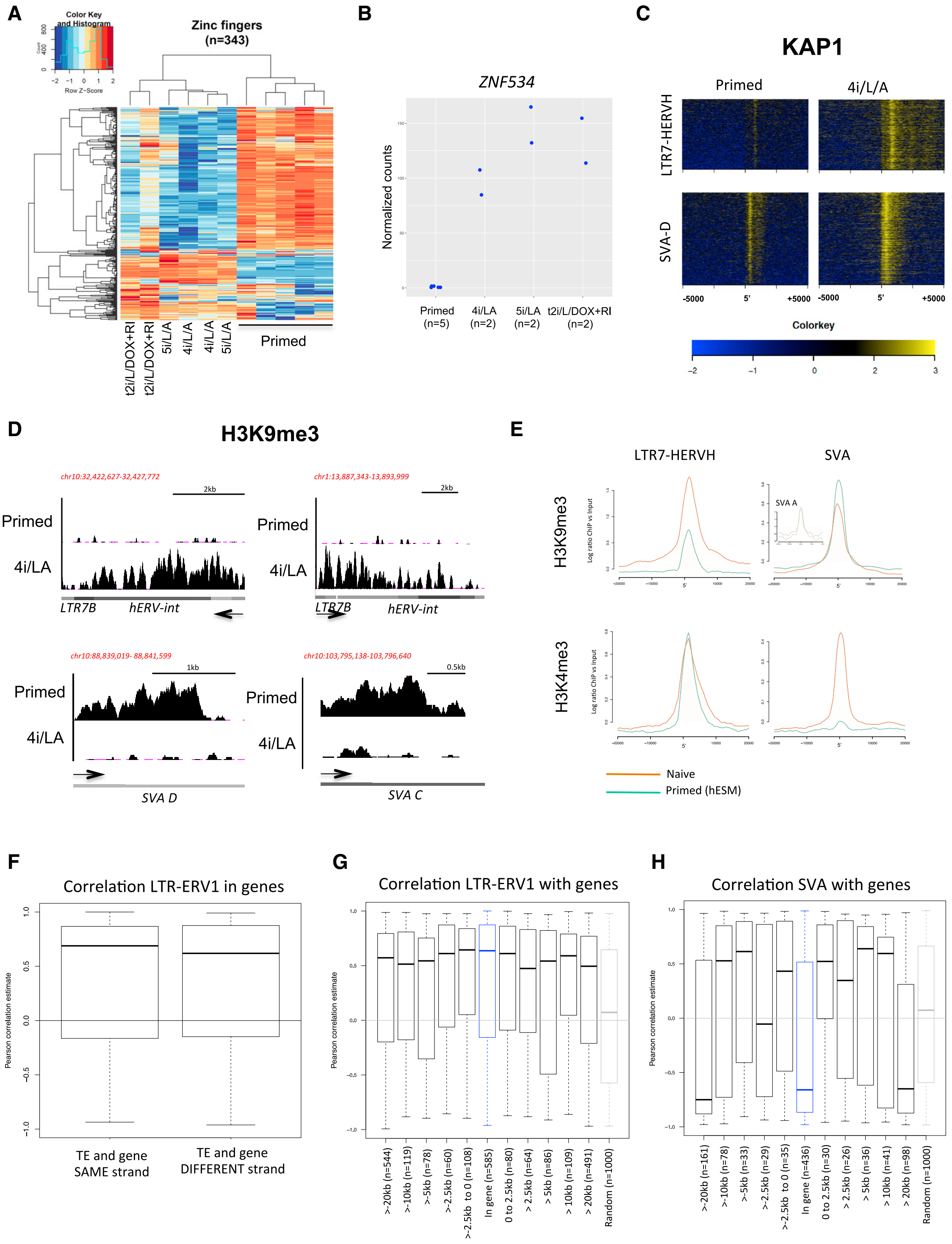

Figure 3. Control of Transposon Transcription in Naive and Primed Human ESCs

(A) Heatmap of RNA-seq data from naive/primed ESCs depicting all KRAB-ZFP genes with expression data (n = 345).

(B) RNA-seq quantification of *ZNF534* in naive and primed ESCs.

(C) Density heatmaps of KAP1 for SVA or LTR7-HERVH. The average profile was determined at ± 20 kb around the 5′ of all elements for each family. This average is calculated from log ratio of ChIP reads over input DNA. KAP1 was chromatin immunoprecipitated in naive (WIBR3 in 4i/L/A) or primed ESC conditions (WIBR3 in hESM). For ChIP, naive cells were cultured in 4i/L/A, as these conditions enabled more rapid large-scale expansion.

(D) Examples of H3K9me3 ChIP signals in naive and primed cells over primed or naive-specific TEs. Screenshot of H3K9me3 ChIP-seq in WIBR3 cells in primed media (hESM) versus naive media (4i/L/A).

(legend continued on next page)

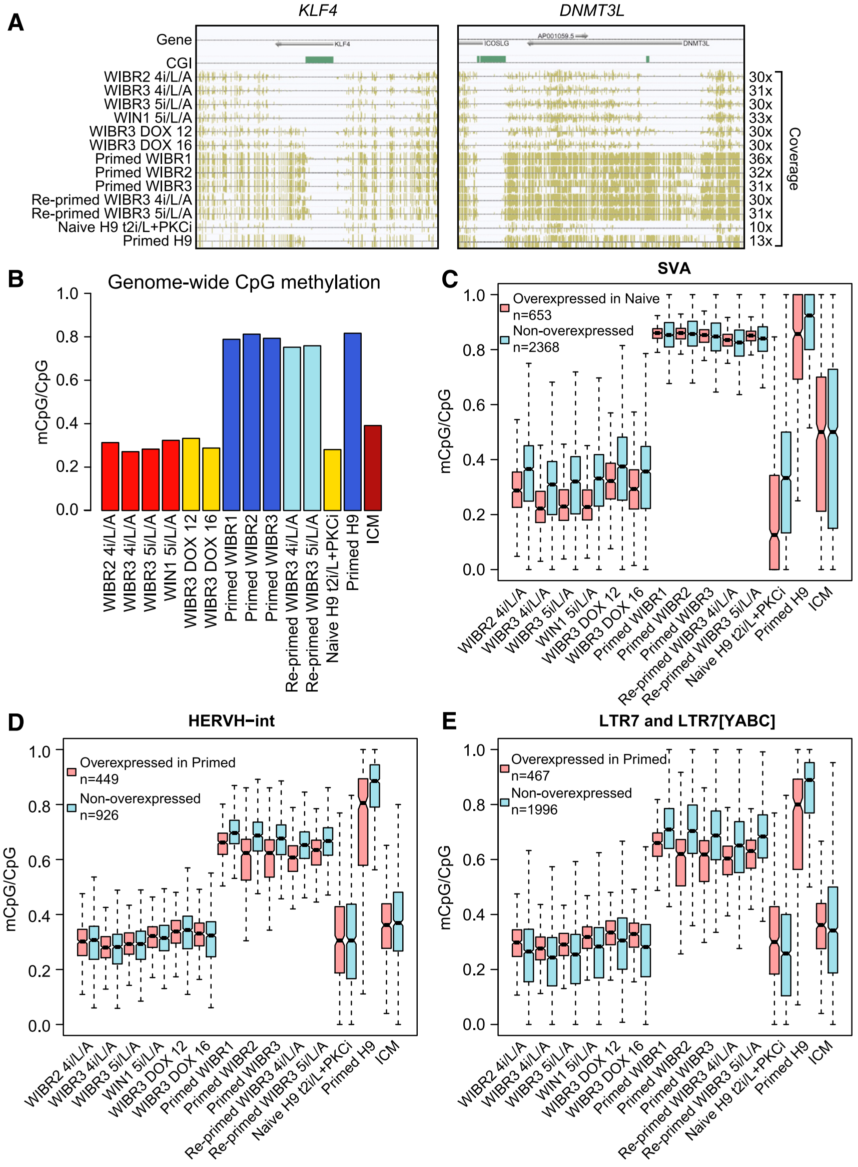

Figure 4. Base-Resolution DNA Methylomes of Naive and Primed Human ESCs

(A) Browser screenshots of two representative regions showing CpG hypomethylation in naive human ESCs compared with primed ESCs. Gene annotations are displayed in the top track. The second track shows the locations of CGIs. CpG methylation is shown as gold tick marks and the heights are proportional to the methylation levels. For each track, ticks projecting upward and downward indicate the methylation on the Watson and Crick strand, respectively. Mean coverage on cytosines in each sample is shown on the right.

(B) Genome-wide CpG methylation level of all samples described in (A) and ICM data from Guo et al. (2014).

(C) Boxplot displaying the methylation levels of SVA copies that were overexpressed in naive versus primed ESCs (red) as well as the methylation state of the non-overexpressed copies (blue). "Overexpressed" defines elements with significantly increased expression in naive ESCs versus primed ESCs (1% FDR; see Supplemental Experimental Procedures for details). Bold line in the center of box indicates the median and notch around it shows the confidence interval (calculated by the "boxplot" function in R). Whiskers indicate maximum or minimum values within 1.5 interquartile range to upper (or lower) quartile.

(D and E) Boxplot displaying the methylation levels of (D) HERVH-int and (E) LTR7 and LTR7[YABC] copies that are overexpressed in primed versus naive human ESCs as well as other non-overexpressed copies from the same family.

See also Figure S4.

and 5B; Table S4). In total, 77% of imprinted DMRs were erased upon 4i/L/A conversion and 71% upon 5i/L/A conversion, where "erased" is defined as regions where the intermediate methylation in starting primed cells becomes hypomethylated (mCpG/CpG < 0.3) in both naive and re-primed cells (Figure 5B; see Supplemental Experimental Procedures). Re-analysis of methylation data from naive human cells in t2i/L+PKCi (Takashima et al., 2014) revealed a similar reduction in CpG methylation at imprinted DMRs: 77% of imprinted DMRs in primed H9 were erased in naive H9 (Table S4). While methylation levels were restored upon re-priming at flanking regions, imprinted DMR methylation remained low regardless of whether the naive state was induced in 5i/L/A or 4i/L/A (Figures 5A–5D and S5A–S5F; Supplemental Experimental Procedures). SNPs revealed the loss of allele-specific methylation at two CpGs in an imprinted DMR near the *SNRPN* gene (Figure S5G; Supplemental Experimental Procedures). We also observed transcriptional amplification of some imprinted genes in naive cells following the combined loss of DNA methylation, KAP1 binding, and H3K9me3 deposition (Figures 5E and 5F; Table S4). The presence of a SNP in a transcribed region of *MEG3* showed that this imprinted gene was expressed

(E) H3K9me3 and H3K4me3 ChIP-seq average profiles for LTR7-HERVH (all LTR7 associated 5′ to an HERVH-int) and all SVA elements. The average profile was determined at ± 20 kb around the 5′ of all those elements. This average corresponds to log ratio of number of reads in chromatin immunoprecipitated over input DNA. H3K9me3 and H3K4me3 were produced from primed ESCs, H3K4me3 in 5i/L/A and JNK inhibitor (6i/L/A) (Theunissen et al., 2014), and H3K9me3 in 4i/L/A (this study).

(F) Boxplot showing TE-gene pair expression correlation for TEs located within genes in either sense or antisense orientations. The correlation is not strand specific. Line in the center of box indicates the median. Whiskers indicate maximum or minimum values within 1.5 interquartile range to upper (or lower) quartile.

(G and H) Boxplots displaying correlation between the expression of LTR-ERV1 integrants (n = 2,324), including HERVH-int (G) or SVA integrants (n = 1003) (H) with expression of closest genes. TEs were categorized according to distance to genes with the following bins: in gene (introns), <2.5, 2.5–5, 5–10, 10–20 kb, and random. Correlation is greatest near genes and tails off as distance increases. Line in the center of box indicates the median. Whiskers indicate maximum or minimum values within 1.5 interquartile range to upper (or lower) quartile.

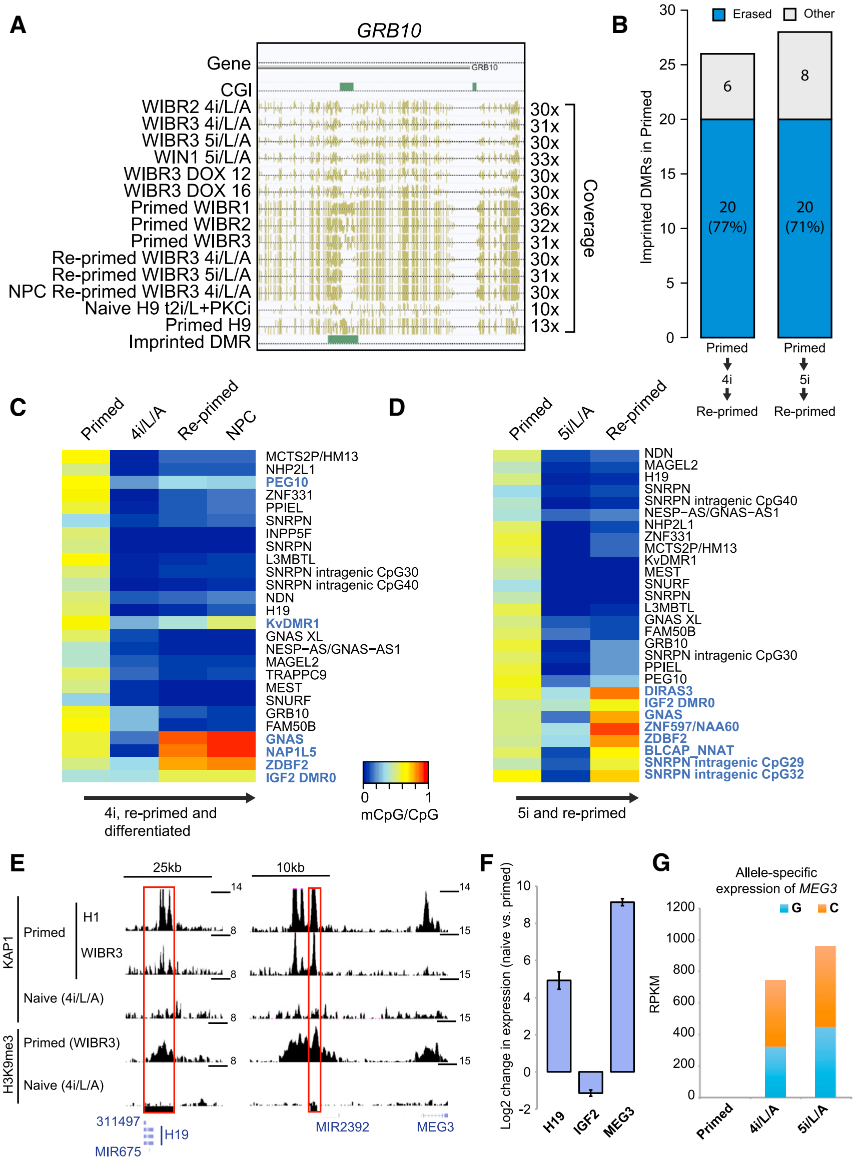

Figure 5. Global DNA Demethylation in Naive Human ESCs Is Reversible except at Imprinted DMRs

(A) Browser screenshot of one representative imprinted DMR where methylation is erased in naive human ESCs and is not restored to primed methylation state in re-primed cells or differentiated cells (NPC re-primed WIBR3 4i/L/A).

(B) The number and fraction of imprinted DMRs in primed WIBR3 that were erased (blue) in both naive and re-primed cells. Gray proportion marks the imprinted DMRs that did not lose DNA methylation in either naive or re-primed ESCs.

(C and D) Heatmaps displaying the CpG methylation levels of imprinted DMRs in WIBR3-primed ESCs before and after conversion in 4i/L/A (C) or WIBR3 (AAVS1-GFP targeted subclone) ESCs before and after conversion in 5i/L/A (D) together with re-primed derivatives. Names of nearby genes for each region are shown on the right. Imprinted DMRs highlighted in blue retained some CpG methylation in either naive or re-primed cells.

(E) KAP1 and H3K9me3 ChIP-seq signals at imprinted DMRs near *H19* and *MEG3* genes in naive and primed ESCs. KAP1 was chromatin immunoprecipitated in naive (WIBR3 4i/LA) or two primed ES conditions (primed WIBR3 from this study and H1 in mTesR1; Turelli et al., 2014). H3K9me3 was chromatin immunoprecipitated in primed WIBR3 ESCs or WIBR3 4i/L/A.

(F) RNA-seq quantification of *H19*, *IGF*, and *MEG3* expression in WIBR2-primed ESCs and naive ESCs in 5i/L/A or 4i/L/A. Error bars indicate ± 1 SD.

(G) SNP analysis shows that *MEG3* is expressed from both alleles in WIBR2 naive ESCs in 5i/L/A or 4i/L/A.

See also Figure S5.

from both alleles in naive cells (Figure 5G). The loss of imprinted DMR methylation in naive human cells indicates that they do not entirely recapitulate the in vivo naive pluripotent state. However, it is possible that maintaining naive cells in culture causes extensive demethylation events and erasure of CpG methylation in imprinted DMRs, which may not occur during the few cell divisions in cleavage embryos.

X Chromosome Status of Female Naive ESCs Resembles the Human Preimplantation Embryo

In the human preimplantation embryo, both X chromosomes are actively transcribed despite expression of *XIST* (Okamoto et al., 2011; Petropoulos et al., 2016). We previously showed that conversion of female primed lines to the naive state results in activation of *XIST*, but reduced expression of X-linked genes suggested the presence of an inactive X chromosome (Theunissen et al., 2014). However, a recent study reported that X chromosome genes maintain biallelic expression while dosage compensation proceeds during human preimplantation development (Petropoulos et al., 2016). Here we re-examined the X chromosome status of naive human cells using methylation data and allele-specific expression methods.

Genes that undergo X inactivation have been shown to acquire DNA methylation at CGIs in promoter regions (Weber et al., 2007). Substantial fractions of X-linked promoter CGIs showed an intermediate methylation level in female primed lines, consistent with the presence of both an unmethylated (active) and methylated (inactive) allele (Figures 6A and S6A). In contrast, female naive cells displayed uniform hypomethylation at X-linked promoter CGIs, similar to male cells (Figures 6A and S6A). Most non-CGI-containing X-linked promoters were not differentially methylated between male and female primed cells, but instead reflected the genome-wide methylation levels of naive and primed states (Figures 6B, S6B, and S6C). The dramatic reduction in methylation of X-linked promoter CGIs suggested that naive conversion may result in X chromosome reactivation.

To distinguish between monoallelic and biallelic expression of X-linked genes, we targeted both alleles of *MECP2* with GFP or tdTomato sequences fused in frame with exon 3 (Figures S6D and S6E). Fluorescent activity was followed in

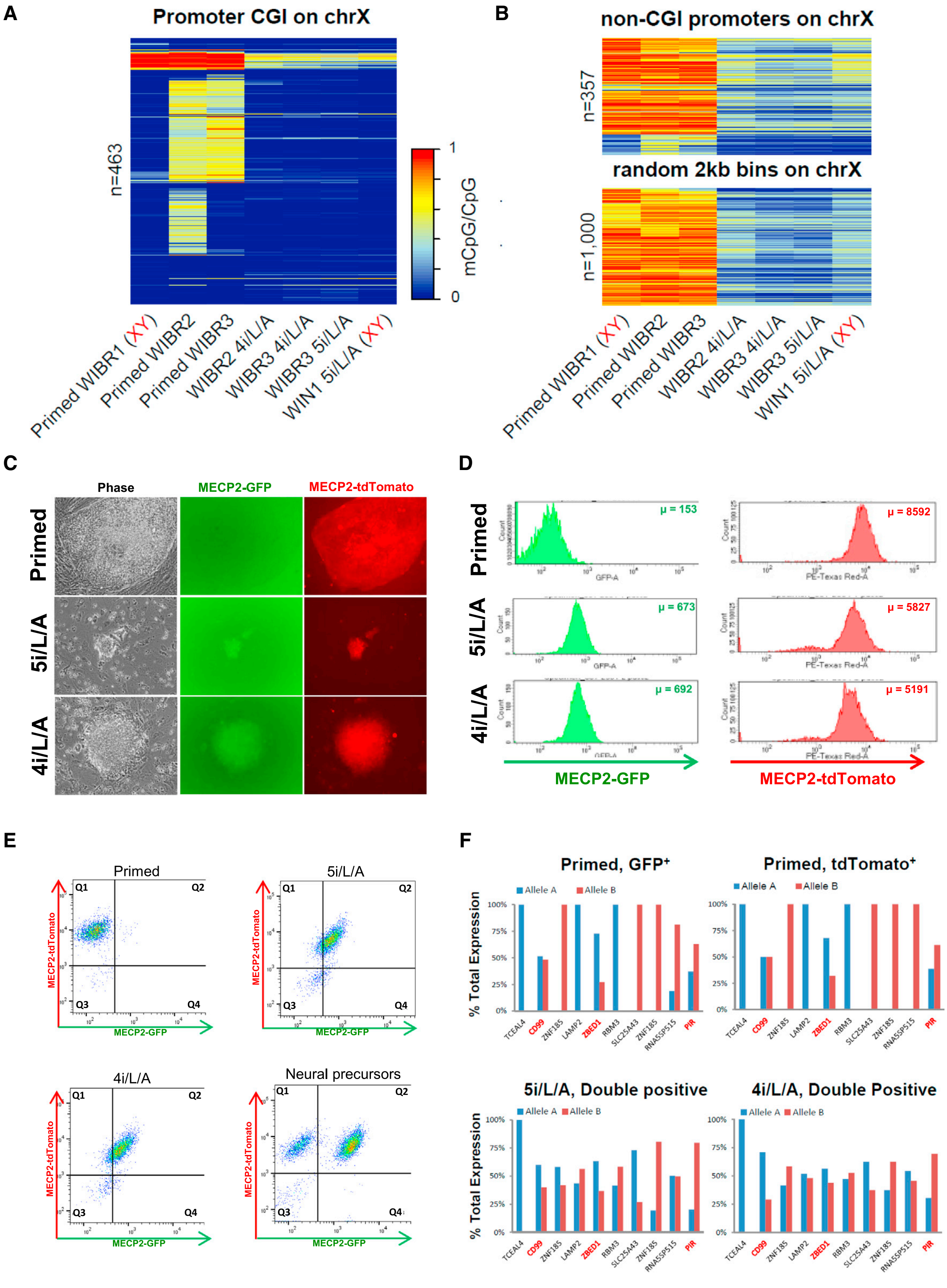

Figure 6. X Chromosome Status of Female Naive Human ESCs

(A) CpG methylation of X-linked promoter CpG islands (CGIs) (n = 463) in naive and primed human ESCs. Male control lines are indicated in red.

(B) CpG methylation levels of non-CGI promoter regions (n = 357) or randomly chosen 2 kb bins (n = 1,000) along the X chromosome in naive and primed human ESCs. The 2 kb bins do not overlap any CGIs or non-CGI promoters. Male control lines are indicated in red.

(C) Phase and fluorescence images of $MECP2^{GFP\text{-}OFF/Tom\text{-}ON}$ primed cells in hESM, and after conversion to the naive state in 5i/L/A and 4i/L/A.

(legend continued on next page)

two independent double-targeted MECP2 reporter clones. The first clone expressed the GFP allele in the primed state but contained a fraction of cells with GFP and tdTomato co-expression. Upon conversion to the naive state, both MECP2 alleles became uniformly expressed (Figures S6F, S6G, and S7A). A second clone that expressed the tdTomato allele in the primed state also displayed activation of both MECP2 alleles upon naive conversion (Figures 6C–6E). Differentiation into neural precursors resulted in the re-appearance of single-color-positive cells (Figure 6E). However, expression was biased toward one of the MECP2 alleles, suggesting that most cells did not undergo random X inactivation upon differentiation.

We used SNPs to quantify allele-specific gene expression in MECP2-reporter cells converted in 5i/L/A and maintained with or without GSK3 inhibition for ten passages. To ensure a direct comparison between naive and primed states, this analysis was restricted to SNP-containing X-linked transcripts with at least ten reads in all samples. Whereas the starting primed cells showed a largely monoallelic expression profile at the interrogated transcripts, the naive cells displayed a shift toward biallelic expression (Figure 6F). As we reported previously (Theunissen et al., 2014), total X-linked gene expression was reduced upon naive conversion when compared to female primed cells (Figure S7B). However, we now realized that X-linked expression also differed substantially between male primed and male naive cells (Figure S7B). To control for the observed variation in transcriptional output, we compared total X-linked expression levels in female naive cells to male naive cells and found that female naive cells had slightly increased X-linked expression (Figure S7C). A recent study described a progressive dampening of expression from both X chromosomes during human preimplantation development, which results in female cells acquiring a total X-linked expression level similar to male cells by the late blastocyst stage (Petropoulos et al., 2016). The observation that dosage compensation is still incomplete in 5i/L/A provides a further indication that this state may correspond most closely to the late-morula or early-blastocyst rather than the late-blastocyst stage, as was suggested from the transposcriptome profile (Figure 2D). In summary, the presence of two active X chromosomes and upregulation of *XIST* (Figure S7D) indicate that naive cells acquire an X chromosome signature close to that observed in early human development (Okamoto et al., 2011; Petropoulos et al., 2016) and suggest that they could be a useful model for studying human X inactivation mechanisms.

A Sensitive Mitochondrial PCR Assay Shows that Naive Human ESCs Rarely Contribute to Interspecies Chimeras after Morula or Blastocyst Injection

Contribution to in vivo development after injection into morula- or blastocyst-stage embryos is the most stringent criterion of naive pluripotent cells in the mouse system. In a previous study, we investigated the potential of 5i/L/A naive human ESCs as well as the naive cells described in Gafni et al. (2013) to contribute to interspecific chimera formation following injection into early mouse embryos, as assessed by the presence of fluorescence in embryos (Theunissen et al., 2014). Because the sensitivity of detecting GFP reporter cells in embryos is low, and because auto-fluorescence may lead to false-positive results, we repeated these experiments using a quantitative and highly sensitive molecular assay based on the detection of an amplified human mitochondrial DNA segment by PCR (Cohen et al., 2016). GFP-labeled naive human ESCs described in Gafni et al. (2013) and tdTomato-labeled naive human ESCs maintained by KLF2 and NANOG transgenes or small molecules were injected at the morula or blastocyst stages. Embryos were isolated between embryonic day 9.5 (E9.5) and E12.5 and inspected under a fluorescence microscope. Dissected embryos were further screened using the PCR assay for human mitochondrial DNA, which has a detection limit of one human cell in 10,000 mouse cells (Cohen et al., 2016). No contribution of human cells was detected after injecting naive cells derived in NHSM (n = 119) (Gafni et al., 2013), 5i/L/A and JNK inhibition (6i/L/A) (n = 224), or DOX-dependent naive cells (n = 244) (Figures 7A, 7B, and 7E). A small number of embryos with discernable human DNA signal were obtained using 5i/L/A (0.7%, n = 139) or 4i/L/A (0.9%, n = 660), indicating the presence of one human cell in 10,000 mouse cells (up to 1 in 2,000 mouse cells in a few embryos; Figures 7C–7E). Thus, though human donor cells were detected in the mouse embryos, the rare incidence of human cell incorporation precluded the examination of their fate. The results summarized in Figure 7E failed to detect a significant difference between the various human donor cells in their low efficiency in colonizing mouse embryos. Moreover, it should be emphasized that the PCR assay can only detect the presence of human DNA but is not suitable to ascertain survival of human cells in the embryo, as the human DNA could be derived from lysed or dead cells.

We conclude that, at least in our hands, the generation of interspecies mouse-human chimeras by injection of naive ESCs into the mouse morula or blastocyst is currently too sporadic for definitive assessment of functional contribution, and we do not propose including it as part of the molecular assessment of naive human pluripotency.

DISCUSSION

Recent years have seen a surge of interest in capturing human ESCs in a naive state more akin to mouse ESCs. However, evidence has accumulated that mouse and human preimplantation development are highly divergent, and that mouse ESCs by extension do not provide an appropriate standard for naive

(D) Histograms reporting the levels of MECP2-GFP and MECP-tdTomato in MECP2$^{GFP-OFF/Tom-ON}$-primed cells in hESM, and after conversion to the naive state in 5i/L/A and 4i/L/A. Note that the maximum intensity of MECP2-GFP is significantly weaker than MECP2-tdTomato.

(E) Flow cytometric analysis showing the distribution of MECP2-GFP and MECP-tdTomato in MECP2$^{GFP-OFF/Tom-ON}$-primed cells in hESM, after conversion to the naive state in 5i/L/A and 4i/L/A, and upon differentiation into neural precursors via a re-primed intermediate step.

(F) Allele-specific gene expression analysis using SNPs located within transcribed regions of X-linked genes in single-color MECP2$^{GFP-ON/Tom-OFF}$- or MECP2$^{GFP-OFF/Tom-ON}$-primed cells in hESM (top) and double-color naive cells maintained in 5i/L/A or 4i/L/A for ten passages (bottom). Red asterisks mark genes reported to escape X inactivation (see Supplemental Experimental Procedures).

See also Figures S6 and S7.

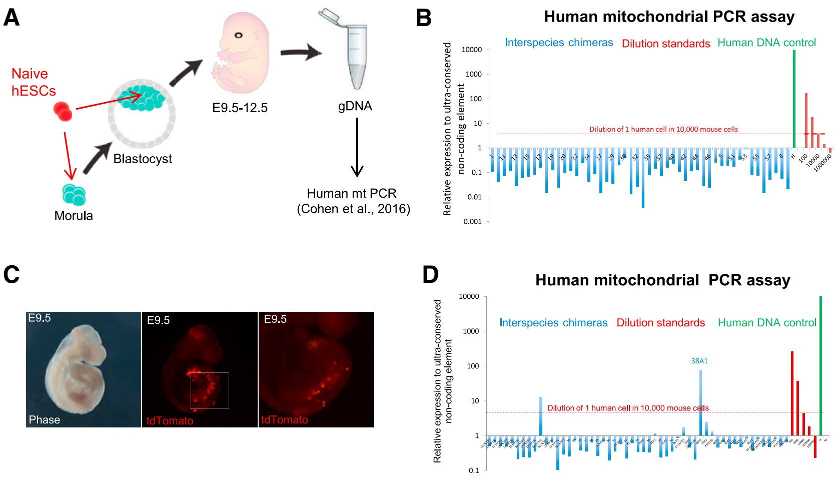

E

Growth condition	Stage of injection	Injected embryos	Dissected embryos	Positive embryos
NHSM	Morula	240	119	0
t2i/L/DOX+RI	Morula	106	94	0
	Blastocyst	333	150	0
6i/L/A	Morula	580	224	0
5i/L/A	Morula	320	108	0
	Blastocyst	65	31	1
4i/L/A	Morula	995	498	4
	Blastocyst	312	162	2
	**Total:**	**2951**	**1386**	**7 (0.5%)**

Figure 7. Assessing the Potential of Naive Human Cells to Generate Interspecies Chimeras Using a Sensitive Assay for Human Mitochondrial DNA

(A) Schematic overview of strategy to assess the contribution of naive human ESCs to interspecific chimera formation after injection into mouse morula- or blastocyst-stage embryos.

(B) Sample qPCR analysis for human mitochondrial DNA following injection of naive human ESCs. This chart shows the lack of human DNA detection after injection of naive human ESCs maintained by inducible overexpression of KLF2 and NANOG transgenes into mouse blastocysts. Embryos were harvested at E10.5. A human DNA control (green bar) and a series of human-mouse cell dilutions (red bars) were run in parallel to estimate the degree of human cell contribution. The dotted line indicates the detection level of human mitochondrial DNA equivalent to a dilution of one human cell in 10,000 mouse cells.

(C) Phase and fluorescence images of a mouse embryo at E9.5 with discernable tdTomato signal following injection with tdTomato-labeled naive ESCs in 4i/L/A at the morula stage and transfer to a pseudopregnant female.

(D) qPCR analysis for human mitochondrial DNA indicated the presence of human cells in mouse embryos at E9.5–E12.5 following injection of naive human ESCs in 4i/L/A at the morula or blastocyst stages. The presence of human cells in embryo 38A1 was confirmed by visual inspection for the presence of tdTomato (C).

(E) Summary of injections of naive human ESCs at the mouse morula or blastocyst stages, numbers of injected and tested embryos, and positive embryos based on a sensitive human mitochondrial PCR assay with a detection threshold of approximately one human cell for every 10,000 mouse cells. Genomic DNA was isolated between E9.5 and E12.5.

human cells. Notable differences in early development between the two species include diapause in mouse (Nichols and Smith, 2009), the failure to restrict hypoblast formation upon FGF/MEK inhibition in humans (Roode et al., 2012), distinct expression patterns of pluripotency and lineage markers (Blakeley et al., 2015; Yan et al., 2013), and the activation of *XIST* in early human embryos (Okamoto et al., 2011; Petropoulos et al., 2016). Here we propose that putative naive human cells should be examined according to their resemblance to human pluripotent cells in vivo. We established stringent criteria for evaluating the naive state of human pluripotency using three comparative molecular parameters for which data from early human embryos are available: (1) the expression profile of transposable elements based on single-cell RNA-seq data (Yan et al., 2013), (2) the DNA methylation landscape of human preimplantation development (Guo et al., 2014), and (3) the X chromosome status of female human embryos (Okamoto et al., 2011; Petropoulos et al., 2016). As an additional criterion, we examined whether the generation of interspecies chimeras by injection of naive human cells into early mouse embryos would provide a functional assay for the naive human pluripotent state.

First, we demonstrate that a comprehensive examination of the transposcriptome offers a more sensitive measure of the correspondence between pluripotent stem cells and the early human embryo than gene expression profiling. As expected from their relative abundance in the genome, differentially expressed TEs between naive and primed human ESCs provided much denser coverage of human chromosomes than differentially expressed genes. Comparison with single-cell RNA-seq data from early human embryos (Yan et al., 2013) revealed a close similarity between naive human ESCs in 5i/L/A and both morula- and epiblast-stage embryos. The upregulation of morula-associated TEs, in particular SVA and LTR5_Hs integrants, was unexpected in view of mounting evidence that epiblast cells of the late blastocyst represent the in vivo equivalent of naive mouse ESCs (Boroviak et al., 2014, 2015). Our findings suggest that 5i/L/A induces a pluripotent state in human cells that corresponds most closely to a late morula or early blastocyst identity. Contrary to previous reports based on naive-like cells derived in 2i/L conditions (Wang et al., 2014), we find that expression of HERVH/LTR7 integrants is more strongly associated with the primed state. We also explored the mechanisms controlling TE expression in naive and primed cells. In some cases, as for the ZNF534-LTR7/HERVH pair, we could match the downregulation of the KRAB-ZFP with KAP1 depletion, loss of repressive marks, and transcriptional activation at the corresponding TE target. While naive human cells in t2i/L+PKCi (Takashima et al., 2014) had a very similar gene expression profile to 5i/L/A, transposcriptome

profiling revealed subtle differences in the expression of developmental stage-specific TE integrants. On the other hand, the NHSM protocol (Gafni et al., 2013) in our hands did not induce activation of blastocyst-associated TEs and appears to induce a distinct state. It will be of interest to explore how specific modifications of the culture environment bring the TE profile of naive human ESCs even closer to that of pluripotent cells in vivo.

Second, we show that a distinctive trademark of naive human ESCs is a genome-wide reduction in DNA methylation to levels equivalent to human eight-cell, morula, and blastocyst embryos. As predicted, expression of the different transposon classes correlated with reduced DNA methylation. DMRs of imprinted genes were differentially methylated in primed ESCs but demethylated in naive ESCs obtained in 5i/L/A or t2i/L+PKCi (Takashima et al., 2014), consistent with global DNA demethylation. However, while global DNA methylation levels were restored when naive cells were converted back to the primed state, imprinted DMRs remained hypomethylated. This is similar to previous results where global demethylation was induced by deletion of Dnmt1 in mouse ESCs, but upon re-expression of Dnmt1 methylation was restored in the bulk genome, but not at imprinted genes (Tucker et al., 1996). Both of these observations are consistent with the strict methylation dependence of the DNA binding of ZFP57, the KRAB-ZFP responsible for tethering the histone and DNA methylation maintenance machinery at imprinting control regions (Quenneville et al., 2011). Once methylation is completely lost, imprinting of the corresponding DMR cannot be restored because the methylation complex can no longer be recruited. While this study was in preparation, Clark and colleagues reported a similar loss of imprinting in naive human ESCs derived in 5i/L/A supplemented with recombinant FGF (Pastor et al., 2016). Loss of imprinting represents a departure from the methylome of pluripotent cells in vivo and limits future applications of naive human ESCs. These results underline the need to identify parameters in the culture environment that protect against extensive demethylation of imprinted DMRs upon long-term maintenance of naive human ESCs.

Third, recent studies have shown that X chromosome dynamics during human preimplantation development are markedly different from mouse, providing a unique blueprint for the naive human pluripotent state. In contrast with early mouse development, human female preimplantation embryos display two active X chromosomes but also express *XIST* (Okamoto et al., 2011; Petropoulos et al., 2016). We found that naive induction in 5i/L/A resulted in X chromosome reactivation, as indicated by reduced DNA methylation at X-linked promoter CGIs, the conversion of single-positive primed MECP2 reporter cells to GFP/tdTomato double-positive naive cells, and biallelic expression of X-linked genes. Unlike other protocols for generating naive-like human cells, 5i/L/A also induced derepression of *XIST*. Our previous conclusion that naive human cells contain an inactive X chromosome was based on a similar level of X-linked gene expression in male primed and female naive cells (Theunissen et al., 2014). However, it has recently been shown that female human embryos undergo progressive dosage compensation from the morula stage onward by downregulation of X-linked genes, resulting in female cells acquiring a total X-linked expression level similar to male cells by the late blastocyst stage (Petropoulos et al., 2016). As female naive cells had slightly elevated total X-linked expression compared to male naive cells, dosage compensation may still be incomplete in 5i/L/A. This provides another indication that these cells may correspond most closely to the late morula or early blastocyst.

Fourth, we used a quantitative and highly sensitive assay based on the detection of human mitochondrial DNA (Cohen et al., 2016) to rigorously assess the potential of naive human ESCs to contribute to interspecific chimera formation after injection into mouse morula- or blastocyst-stage embryos. Contribution to mouse-human chimeras has been proposed as an in vivo assay for developmental potency of naive human cells, as shown for rat ESCs (Kobayashi et al., 2010) and monkey iPSCs (Fang et al., 2014). In recent experiments, primed human ESCs distributed into different parts of the host epiblast when introduced into postimplantation mouse embryos cultured in vitro for 2 days (Mascetti and Pedersen, 2016; Wu et al., 2015). This suggested that matching the developmental stage of donor cells and host embryo may be important for integration of the cells into the embryo. In further support of this notion, when human neural crest cells were microinjected into mouse embryos at E8.5, the stage when the host neural crest cells leave the neural tube, pigmentation in post-natal chimeras revealed functional integration of the donor cells (Cohen et al., 2016). Here we injected a large number of mouse morula- or blastocyst-stage embryos (n = 2,951) with naive human cells generated in 6i/L/A, 5i/L/A, 4i/L/A, or KLF2/NANOG transgenic conditions and NHSM cells (Gafni et al., 2013). Only a small fraction of E9.5–E12.5 embryos was found to carry between one to at most five human cells per 10,000 mouse cells. Thus, while it may be possible to generate low-grade interspecies mouse-human chimeras, at least in our hands, the efficiency is currently too low for any definitive assessment of functional contribution of the human cells. Achieving efficient incorporation of human cells into mouse embryos may require "humanization" by constructing a host expressing suitable factors or niches that support the functional integration of human ESCs into interspecies chimeras.

In conclusion, we have outlined a set of molecular criteria for evaluating the naive state of human pluripotency. The gold standard for pluripotency in mouse is chimera formation. Our attempts to achieve functional integration of human pluripotent cells into interspecies chimeras were not successful, emphasizing the need for other defining criteria, such as transcriptional and epigenetic features of the human preimplantation embryo. Our results suggest that naive human ESCs provide a unique model system to dissect mechanisms of early human development that cannot be studied in mouse (due to evolutionary differences) or in conventional human ESCs (due to phenotypic drift from the embryo). For example, naive human ESCs provide a cellular model to interrogate the function of transposable elements that become activated during early human development. SVA integrants, which are highly enriched in the human cleavage embryo and appear to influence gene expression in naive human ESCs, are an evolutionary innovation of hominids. In addition, the vast majority of human KRAB-ZFPs target TEs not found in rodents and therefore have no ortholog in mouse, including the HERVH-controlling ZNF534. Naive human cells also provide a unique system to study X chromosome regulation during human embryogenesis, as both X chromosomes are actively

transcribed despite upregulation of *XIST*. On the other hand, our analyses also highlight some notable differences between current naive human cells and the embryo, such as loss of methylation at imprinted DMRs and non-random X chromosome inactivation upon differentiation. These criteria provide a focus for future efforts to further refine the growth conditions for naive human cells and establish a cellular model that fully recapitulates the state of human pluripotent cells in vivo.

EXPERIMENTAL PROCEDURES

Cell Culture

Conventional (primed) human ESC lines were maintained on mitomycin C inactivated mouse embryonic fibroblast (MEF) feeders in human ESC medium (hESM) and passaged mechanically using a drawn Pasteur pipette or enzymatically by treatment for 20 min with 1 mg/mL collagenase type IV (GIBCO), followed by sequential sedimentation steps in hESM to remove single cells. Naive human ESCs were cultured on mitomycin C-inactivated MEF feeder cells and were passaged by a brief PBS wash followed by single-cell dissociation using 3–5 min treatment with Accutase (GIBCO) and centrifugation in fibroblast medium. For conversion of pre-existing primed human ESC lines, we seeded 2×10^5 trypsinized single cells on an MEF feeder layer in hESM supplemented with ROCK inhibitor Y-27632 (Stemgent, 10 μM). Two days later, medium was switched to 5i/L/A- or 4i/L/A (no IM12)-containing naive hESM. Following an initial wave of cell death, naive colonies appeared within 10 days and were expanded polyclonally using Accutase (GIBCO) on an MEF feeder layer. Naive cells generated with DOX-inducible KLF2 and NANOG transgenes (Theunissen et al., 2014) were maintained in 2i/L/DOX with optional inclusion of ROCK inhibitor Y-27632 (Stemgent, 10 μM). To adapt DOX-dependent naive cells to transgene-free culture conditions, 1×10^5 single cells were seeded on an MEF feeder layer in 2i/L/DOX, and DOX was withdrawn the next day. For re-priming, semi-confluent cultures of naive cells were switched to hESM with ROCK inhibitor Y-27632 (Stemgent, 10 μM) and passaged with collagenase on MEFs. Neuronal precursor cells (NPCs) were derived from re-primed cells using a published differentiation protocol (Cohen et al., 2007). To culture naive human cells described by Gafni et al. (2013), we used the KSR-based version of the NHSM protocol. Tissue culture media were filtered using a low protein-binding 0.22 μm filter (Corning). All experiments in this paper were performed under physiological oxygen conditions (5% O_2, 3% CO_2). Detailed media compositions are described in Supplemental Experimental Procedures. All animal experiments were performed in compliance with protocol #1031-088-16 from the Committee on Animal Care at MIT. In addition, interspecies chimerism experiments were approved by the Embryonic Stem Cell Research Oversight (ESCRO) Committee at Whitehead Institute.

ACCESSION NUMBERS

The accession number for the gene expression, methylation, and ChIP-seq analyses in this paper is GEO: GSE75868.

SUPPLEMENTAL INFORMATION

Supplemental Information includes Supplemental Experimental Procedures, seven figures, and four tables and can be found with this article online at http://dx.doi.org/10.1016/j.stem.2016.06.011.

AUTHOR CONTRIBUTIONS

T.W.T., M.F., Y. He, D.T., J.R.E., and R.J. conceived of the study and designed experiments. T.W.T. developed naive conditions and performed tissue culture experiments. E.P., M.F., J. Duc, and D.T. developed transposcriptome methodology. E.P., A.I., J.P., and M.F. matched transposcriptome with public RNA-seq datasets. Y. He, R.C.O., R.C., Z.Z., J.R.N., and J.R.E. performed methylation analysis. J.P., M.I., and M.F performed chromatin IP analysis. H.W. performed gene targeting experiments. S.M., T.W.T., M.A.C., K.J.W., J. Drotar, and T.L. performed and analyzed embryo injections. Y. Huang performed bioinformatic analyses of X chromosome status. T.W.T., R.J., M.F., Y. He, and R.C.O. wrote the manuscript with input from D.T. and J.R.E.

ACKNOWLEDGMENTS

We thank Ruth Flannery for embryo processing, Patti Wisniewski and Colin Zollo for cell sorting, the University of Lausanne Genomic core facility for sequencing, and Dr. Jacob Hanna (Weizmann Institute) for sharing cells. This study was supported by grants from the Simons Foundation (SFLIFE #286977 to R.J.) and in part by the NIH (RO1-CA084198) to R.J., and from the Swiss National Science Foundation and the European Research Council (KRABnKAP, No. 268721) to D.T. The work in the J.R.E. laboratory was supported by the Howard Hughes Medical Institute and Gordon and Betty Moore Foundation (GBMF3034) and the Mary K. Chapman Foundation. J.R.E. is an Investigator of the Howard Hughes Medical Institute. T.W.T. is supported by a Sir Henry Wellcome Postdoctoral Fellowship (098889/Z/12/Z), J.P. by a Foundation Bettencourt Award and by the Association pour la Recherche sur le Cancer (ARC), and M.I. by a postdoctoral training grant from the Fonds de la Recherche en Santé du Québec. R.J. is co-founder of Fate Therapeutics and an adviser to Stemgent.

Received: February 10, 2016
Revised: April 15, 2016
Accepted: June 17, 2016
Published: July 14, 2016

REFERENCES

Bai, Q., Assou, S., Haouzi, D., Ramirez, J.M., Monzo, C., Becker, F., Gerbal-Chaloin, S., Hamamah, S., and De Vos, J. (2012). Dissecting the first transcriptional divergence during human embryonic development. Stem Cell Rev. *8*, 150–162.

Blakeley, P., Fogarty, N.M., del Valle, I., Wamaitha, S.E., Hu, T.X., Elder, K., Snell, P., Christie, L., Robson, P., and Niakan, K.K. (2015). Defining the three cell lineages of the human blastocyst by single-cell RNA-seq. Development *142*, 3151–3165.

Boroviak, T., Loos, R., Bertone, P., Smith, A., and Nichols, J. (2014). The ability of inner-cell-mass cells to self-renew as embryonic stem cells is acquired following epiblast specification. Nat. Cell Biol. *16*, 516–528.

Boroviak, T., Loos, R., Lombard, P., Okahara, J., Behr, R., Sasaki, E., Nichols, J., Smith, A., and Bertone, P. (2015). Lineage-specific profiling delineates the emergence and progression of naive pluripotency in mammalian embryogenesis. Dev. Cell *35*, 366–382.

Chan, Y.S., Göke, J., Ng, J.H., Lu, X., Gonzales, K.A., Tan, C.P., Tng, W.Q., Hong, Z.Z., Lim, Y.S., and Ng, H.H. (2013). Induction of a human pluripotent state with distinct regulatory circuitry that resembles preimplantation epiblast. Cell Stem Cell *13*, 663–675.

Cohen, M.A., Itsykson, P., and Reubinoff, B.E. (2007). Neural differentiation of human ES cells. Curr. Protoc. Cell Biol. http://dx.doi.org/10.1002/0471143030.cb2307s36.

Cohen, M.A., Wert, K.J., Goldmann, J., Markoulaki, S., Buganim, Y., Fu, D., and Jaenisch, R. (2016). Human neural crest cells contribute to coat pigmentation in interspecies chimeras after in utero injection into mouse embryos. Proceedings of the National Academy of Sciences of the United States of America *113*, 1570–1575. http://dx.doi.org/10.1073/pnas.1525518113.

Court, F., Tayama, C., Romanelli, V., Martin-Trujillo, A., Iglesias-Platas, I., Okamura, K., Sugahara, N., Simón, C., Moore, H., Harness, J.V., et al. (2014). Genome-wide parent-of-origin DNA methylation analysis reveals the intricacies of human imprinting and suggests a germline methylation-independent mechanism of establishment. Genome Res. *24*, 554–569.

De Los Angeles, A., Ferrari, F., Xi, R., Fujiwara, Y., Benvenisty, N., Deng, H., Hochedlinger, K., Jaenisch, R., Lee, S., Leitch, H.G., et al. (2015). Hallmarks of pluripotency. Nature *525*, 469–478.

Fang, R., Liu, K., Zhao, Y., Li, H., Zhu, D., Du, Y., Xiang, C., Li, X., Liu, H., Miao, Z., et al. (2014). Generation of naive induced pluripotent stem cells from rhesus monkey fibroblasts. Cell Stem Cell *15*, 488–496.

Friedli, M., and Trono, D. (2015). The developmental control of transposable elements and the evolution of higher species. Annu. Rev. Cell Dev. Biol. *31*, 429–451.

Gafni, O., Weinberger, L., Mansour, A.A., Manor, Y.S., Chomsky, E., Ben-Yosef, D., Kalma, Y., Viukov, S., Maza, I., Zviran, A., et al. (2013). Derivation of novel human ground state naive pluripotent stem cells. Nature *504*, 282–286.

Guo, H., Zhu, P., Yan, L., Li, R., Hu, B., Lian, Y., Yan, J., Ren, X., Lin, S., Li, J., et al. (2014). The DNA methylation landscape of human early embryos. Nature *511*, 606–610.

Hancks, D.C., and Kazazian, H.H., Jr. (2010). SVA retrotransposons: evolution and genetic instability. Semin. Cancer Biol. *20*, 234–245.

Huang, K., Maruyama, T., and Fan, G. (2014). The naive state of human pluripotent stem cells: a synthesis of stem cell and preimplantation embryo transcriptome analyses. Cell Stem Cell *15*, 410–415.

Iyengar, S., and Farnham, P.J. (2011). KAP1 protein: an enigmatic master regulator of the genome. J. Biol. Chem. *286*, 26267–26276.

Kobayashi, T., Yamaguchi, T., Hamanaka, S., Kato-Itoh, M., Yamazaki, Y., Ibata, M., Sato, H., Lee, Y.S., Usui, J., Knisely, A.S., et al. (2010). Generation of rat pancreas in mouse by interspecific blastocyst injection of pluripotent stem cells. Cell *142*, 787–799.

Lee, H.J., Hore, T.A., and Reik, W. (2014). Reprogramming the methylome: erasing memory and creating diversity. Cell Stem Cell *14*, 710–719.

Lister, R., Pelizzola, M., Dowen, R.H., Hawkins, R.D., Hon, G., Tonti-Filippini, J., Nery, J.R., Lee, L., Ye, Z., Ngo, Q.M., et al. (2009). Human DNA methylomes at base resolution show widespread epigenomic differences. Nature *462*, 315–322.

Mascetti, V.L., and Pedersen, R.A. (2016). Human-mouse chimerism validates human stem cell pluripotency. Cell Stem Cell *18*, 67–72.

Matsui, T., Leung, D., Miyashita, H., Maksakova, I.A., Miyachi, H., Kimura, H., Tachibana, M., Lorincz, M.C., and Shinkai, Y. (2010). Proviral silencing in embryonic stem cells requires the histone methyltransferase ESET. Nature *464*, 927–931.

Nichols, J., and Smith, A. (2009). Naive and primed pluripotent states. Cell Stem Cell *4*, 487–492.

Okamoto, I., Patrat, C., Thépot, D., Peynot, N., Fauque, P., Daniel, N., Diabangouaya, P., Wolf, J.P., Renard, J.P., Duranthon, V., and Heard, E. (2011). Eutherian mammals use diverse strategies to initiate X-chromosome inactivation during development. Nature *472*, 370–374.

Pastor, W.A., Chen, D., Liu, W., Kim, R., Sahakyan, A., Lukianchikov, A., Plath, K., Jacobsen, S.E., and Clark, A.T. (2016). Naive human pluripotent cells feature a methylation landscape devoid of blastocyst or germline memory. Cell Stem Cell *18*, 323–329.

Petropoulos, S., Edsgärd, D., Reinius, B., Deng, Q., Panula, S.P., Codeluppi, S., Plaza Reyes, A., Linnarsson, S., Sandberg, R., and Lanner, F. (2016). Single-cell RNA-seq reveals lineage and X chromosome dynamics in human preimplantation embryos. Cell *165*, 1012–1026.

Quenneville, S., Verde, G., Corsinotti, A., Kapopoulou, A., Jakobsson, J., Offner, S., Baglivo, I., Pedone, P.V., Grimaldi, G., Riccio, A., and Trono, D. (2011). In embryonic stem cells, ZFP57/KAP1 recognize a methylated hexanucleotide to affect chromatin and DNA methylation of imprinting control regions. Mol. Cell *44*, 361–372.

Roode, M., Blair, K., Snell, P., Elder, K., Marchant, S., Smith, A., and Nichols, J. (2012). Human hypoblast formation is not dependent on FGF signalling. Dev. Biol. *361*, 358–363.

Rowe, H.M., Jakobsson, J., Mesnard, D., Rougemont, J., Reynard, S., Aktas, T., Maillard, P.V., Layard-Liesching, H., Verp, S., Marquis, J., et al. (2010). KAP1 controls endogenous retroviruses in embryonic stem cells. Nature *463*, 237–240.

Singh, K., Cassano, M., Planet, E., Sebastian, S., Jang, S.M., Sohi, G., Faralli, H., Choi, J., Youn, H.D., Dilworth, F.J., and Trono, D. (2015). A KAP1 phosphorylation switch controls MyoD function during skeletal muscle differentiation. Genes Dev. *29*, 513–525.

Takashima, Y., Guo, G., Loos, R., Nichols, J., Ficz, G., Krueger, F., Oxley, D., Santos, F., Clarke, J., Mansfield, W., et al. (2014). Resetting transcription factor control circuitry toward ground-state pluripotency in human. Cell *158*, 1254–1269.

Theunissen, T.W., Powell, B.E., Wang, H., Mitalipova, M., Faddah, D.A., Reddy, J., Fan, Z.P., Maetzel, D., Ganz, K., Shi, L., et al. (2014). Systematic identification of culture conditions for induction and maintenance of naive human pluripotency. Cell Stem Cell *15*, 471–487.

Tucker, K.L., Beard, C., Dausmann, J., Jackson-Grusby, L., Laird, P.W., Lei, H., Li, E., and Jaenisch, R. (1996). Germ-line passage is required for establishment of methylation and expression patterns of imprinted but not of nonimprinted genes. Genes Dev. *10*, 1008–1020.

Turelli, P., Castro-Diaz, N., Marzetta, F., Kapopoulou, A., Raclot, C., Duc, J., Tieng, V., Quenneville, S., and Trono, D. (2014). Interplay of TRIM28 and DNA methylation in controlling human endogenous retroelements. Genome Res. *24*, 1260–1270.

Urich, M.A., Nery, J.R., Lister, R., Schmitz, R.J., and Ecker, J.R. (2015). MethylC-seq library preparation for base-resolution whole-genome bisulfite sequencing. Nat. Protoc. *10*, 475–483.

Wang, J., Xie, G., Singh, M., Ghanbarian, A.T., Raskó, T., Szvetnik, A., Cai, H., Besser, D., Prigione, A., Fuchs, N.V., et al. (2014). Primate-specific endogenous retrovirus-driven transcription defines naive-like stem cells. Nature *516*, 405–409.

Ware, C.B., Nelson, A.M., Mecham, B., Hesson, J., Zhou, W., Jonlin, E.C., Jimenez-Caliani, A.J., Deng, X., Cavanaugh, C., Cook, S., et al. (2014). Derivation of naive human embryonic stem cells. Proc. Natl. Acad. Sci. USA *111*, 4484–4489.

Weber, M., Hellmann, I., Stadler, M.B., Ramos, L., Pääbo, S., Rebhan, M., and Schübeler, D. (2007). Distribution, silencing potential and evolutionary impact of promoter DNA methylation in the human genome. Nat. Genet. *39*, 457–466.

Wu, J., and Izpisua Belmonte, J.C. (2015). Dynamic pluripotent stem cell states and their applications. Cell Stem Cell *17*, 509–525.

Wu, J., Okamura, D., Li, M., Suzuki, K., Luo, C., Ma, L., He, Y., Li, Z., Benner, C., Tamura, I., et al. (2015). An alternative pluripotent state confers interspecies chimaeric competency. Nature *521*, 316–321.

Yan, L., Yang, M., Guo, H., Yang, L., Wu, J., Li, R., Liu, P., Lian, Y., Zheng, X., Yan, J., et al. (2013). Single-cell RNA-seq profiling of human preimplantation embryos and embryonic stem cells. Nat. Struct. Mol. Biol. *20*, 1131–1139.

Cell Stem Cell, Volume *19*

Supplemental Information

Molecular Criteria for Defining the Naive Human Pluripotent State

Thorold W. Theunissen, Marc Friedli, Yupeng He, Evarist Planet, Ryan C. O'Neil, Styliani Markoulaki, Julien Pontis, Haoyi Wang, Alexandra Iouranova, Michaël Imbeault, Julien Duc, Malkiel A. Cohen, Katherine J. Wert, Rosa Castanon, Zhuzhu Zhang, Yanmei Huang, Joseph R. Nery, Jesse Drotar, Tenzin Lungjangwa, Didier Trono, Joseph R. Ecker, and Rudolf Jaenisch

Supplemental Information

Molecular Criteria for Defining the Naive Human Pluripotent State

Thorold W. Theunissen[1,*], Marc Friedli[2,*], Yupeng He[3,*], Evarist Planet[2], Ryan Oneil[3], Styliani Markoulaki[1], Julien Pontis[2], Haoyi Wang[1,‡], Alexandra Iouranova[2], Michaël Imbeault[2], Julien Duc[2], Malkiel A. Cohen[1], Katherine J. Wert[1], Rosa G. Castanon[3], Zhuzhu Zhang[3], Yanmei Huang[1], Joseph R. Nery[3], Jesse Drotar[1], Tenzin Lungjangwa[1], Didier Trono[2,#], Joseph R. Ecker[3,#] and Rudolf Jaenisch[1,4,#]

Contents:

I. Supplemental Experimental Procedures

II. Supplemental Figure Legends

III. Supplemental Table Legends

IV. Supplemental Figures

V. Supplemental References

I. Supplemental Experimental Procedures

Culture conditions

Conventional (primed) human ESCs were maintained on mitomycin C inactivated mouse embryonic fibroblast (MEF) feeder layers and passaged using Collagenase (1 mg/mL) or manual methods. Primed human ESCs and human iPSCs were cultured in human ESC medium (hESM) [DMEM/F12 (Invitrogen) supplemented with 15% fetal bovine serum (Gibco HI FBS, 10082-147), 5% KnockOut Serum Replacement (Invitrogen), 2 mM L-glutamine (MPBio), 1% nonessential amino acids (Invitrogen), 1% penicillin-streptomycin (Lonza), 0.1 mM β-mercaptoethanol (Sigma) and 4 ng/ml FGF2 (R&D systems)]. Naive human ESCs were cultured on mitomycin C-inactivated MEF

feeder cells, and were passaged by a brief PBS wash followed by single-cell dissociation using 3-5 minute treatment with Accutase (Gibco) and centrifugation in fibroblast medium [DMEM (Invitrogen) supplemented with 10% FBS (Gibco HI FBS, 10082-147), 2 mM L-glutamine (MPBio), 1% nonessential amino acids (Invitrogen), 1% penicillin-streptomycin (Lonza) and 0.1 mM β-mercaptoethanol (Sigma)]. For conversion of pre-existing primed human ESC lines, we seeded 2 x 10^5 trypsinized single cells on a MEF feeder layer in hESM supplemented with ROCK inhibitor Y-27632 (Stemgent, 10 μM). 2 days later medium was switched to 5i/L/A or 4i/L/A (no IM12)-containing naive human ESC medium. Following an initial wave of cell death, naive colonies appeared within 10 days and were expanded polyclonally using Accutase (Gibco) on a MEF feeder layer. Naive human pluripotent cells were derived and maintained in serum-free N2B27-based media supplemented with inhibitors and cytokines. 500 mL of medium was generated by including: 240 mL DMEM/F12 (Invitrogen; 11320), 240 mL Neurobasal (Invitrogen; 21103), 5 mL N2 supplement (Invitrogen; 17502048), 10 mL B27 supplement (Invitrogen; 17504044), 10 μg recombinant human LIF (made in-house), 2 mM L-glutamine (MPBio), 1% nonessential amino acids (Invitrogen), 0.1 mM β-mercaptoethanol (Sigma), 1% penicillin-streptomycin (Lonza), 50 μg/ml BSA (Gibco Fraction V, 15260), and the following small molecules and cytokines: PD0325901 (Stemgent, 1 μM), IM-12 (Enzo, 1 μM or 0.5 μM), SB590885 (R&D systems, 0.5 μM), WH4-023 (A Chemtek or Selleckchem, 1 μM), Y-27632 (Stemgent, 10 μM) and Activin A (Peprotech, 10 ng/mL). Additional inhibitors described in this work include: GSK3 inhibitor CHIR99021 (Stemgent, 1 μM or 0.3 μM), JNK inhibitor SP600125 (R&D systems, 10 μM) and doxycycline (DOX) (Sigma, 2 μg/ml). Naive cells generated with DOX-inducible KLF2 and NANOG transgenes (Theunissen et al., 2014) were maintained in 1 μM PD0325901, 0.3 μM (t) or 1 μM CHIR99021, 20 ng/ml hLIF and 2 μg/ml DOX with optional inclusion of 10 μM ROCK inhibitor Y-27632 (t2i/L/DOX+RI). To adapt DOX-dependent naive cells to transgene-free culture conditions, 1 x 10^5 single cells were seeded on a MEF feeder layer in 2i/L/DOX and DOX was withdrawn the next day. For re-priming, semi-confluent cultures of naive cells were switched to hESM with ROCK inhibitor Y-27632 (10 μM) and passaged with Collagenase on MEFs. Neuronal precursor cells (NPCs) were derived from re-primed cells using a published

differentiation protocol (Cohen et al., 2007). To culture naive human cells described by Gafni et al. (2013), we used the KSR-based version of NHSM (updated version 15/12/2013 provided by Dr. Jacob Hanna's laboratory), consisting of KO-DMEM (Invitrogen) supplemented with 18% KSR (Invitrogen), 1% penicillin-streptomycin (Invitrogen), 1% L-glutamine (Invitrogen), 1% NEAA (Invitrogen), 0.1 mM β-mercaptoethanol (Sigma), 12.5 µg/mL recombinant human insulin (Sigma) and the following cytokines and inhibitors: 20 ng/mL human LIF (Peprotech), 8 ng/mL FGF2 (R&D), 2 ng/mL TGFβ1 (Peprotech), 3 µM Chir99021 (Axon Medchem), 1 µM PD0325901 (Axon Medchem), 2 µM BIRB796 (Axon Medchem), 10 µM SP600125 (Tocris), 5 µM Y-27632 (Axon Medchem) and 5 µM Go6983 (Tocris). Tissue culture media were filtered using a low protein-binding binding 0.22 µm filter (Corning). All experiments in this paper were performed under physiological oxygen conditions (5% O_2, 3% CO_2).

TALEN design and construction

TALENs were designed and assembled as previously described (Hockemeyer et al., 2011). Candidate TALENs constructs were transiently transfected into HEK293 cells, and active TALENs targeting the desired location at high efficiency were identified using a Surveyor assay. The sequences of primers used in the Surveyor assay are gcagactggcatgttctctgtg and tgctccatgagggatccttgtcc. The DNA binding sites of the TALENs used for targeting the exon 3 of MECP2 are gcagccatcagcccaccact and ctctgctttgcctgcct. To target both MECP2 alleles in WIBR2 human ESCs, two donor constructs were generated. One donor construct was engineered to contain an eGFP-polyA sequence in-frame with MECP2 exon 3 and a PGK-puro-polyA cassette flanked by two homology arms corresponding to the genomic sequence of MECP2. The other donor construct contains a tdTomato-polyA sequence along with a PGK-neo-polyA cassette, flanked by the same homology arms.

Genome editing in human ESCs

TALEN-mediated gene targeting was performed as previously described (Hockemeyer et al., 2011). Briefly, WIBR2 ESCs were cultured in Rho-associated protein kinase

(ROCK) inhibitor for 24 hours prior to electroporation. Cells were harvested and dissociated into single cells using trypsin/EDTA solution, followed by re-suspension in PBS. Electroporation was performed using 40ug of donor plasmid and 5ug of each TALEN plasmid. Cells were plated on DR4 MEF feeders, and cultured in maintenance medium containing ROCK inhibitor for the first 24 hours. Puromycin or G418 was added to maintenance medium 48 hours after electroporation, and individual resistant clones were picked and expanded. Southern blots were performed to identify correctly targeted clones, using 32P random primer-labeled probes (Stratagene) against 5' or 3' external sequences, or GFP (internal). Two independently double-targeted MECP2 reporter primed hESC clones were established. 29M-GP26-TN9 was established by targeting the first MECP2 allele using GFP (29M-GP), and then targeting the other allele with tdTomato. 29M-TN3-GP8 was established by targeting the first MECP2 allele using tdTomato (29M-TN), and then targeting the other allele with GFP.

Flow cytometry

Single cell suspensions were filtered, stained with DAPI (Life Technologies), and assessed on the LSRFortessa SORP (Beckton-Dickinson, San Jose, CA). PE-Texas Red (for detection of autofluorescence) was excited by a Coherent Compass 561 nm (50 mW) yellow/green laser and detected using a bandpass filter (emission) of 610/20, GFP was excited by a Coherent Sapphire Solid State 488 nm (100 mW) blue laser and detected using a bandpass filter (emission) of 530/30, and DAPI was excited by a Lightwave Xcyte 355 nm (60 mW) UV laser and detected using a bandpass filter (emission) of 450/50.

Interspecific embryo injections

Donor cells for interspecific embryos injection

Interspecific embryo injections were performed with the following constitutively labeled human ESC lines and culture conditions: C1 AAVS1-GFP iPSCs and WIBR3 AAVS1-GFP ESCs in KSR-based NHSM media (Gafni et al., 2013), WIBR3 AAVS1-GFP ESCs in 5i/L/A or 6i/L/A (with JNK inhibitor), WIBR3 AAVS1-tdTomato ESCs with inducible

KLF2 and NANOG transgenes in t2i/L/DOX+RI (1 µM PD0325901, 0.3 µM CHIR99021, 10 µM Y27632) or 4i/L/A after DOX withdrawal.

Morula and blastocyst injection

Embryo injections were performed using (C57Bl/6xDBA) B6D2F2 host embryos, either at the blastocyst or morula stage. For both types of injections, B6D2F1 females were hormone primed by an i.p. injection of PMS (Pregnant Mare Serum Gonadotropin, EMD Millipore) followed 46h later by an injection of hCG (human Chorionic Gonadrotropin, VWR). After mating with stud males, embryos were harvested at the one cell stage. For morula stage injections the embryos were cultured in a CO_2 incubator for 2 nights, whereas for blastocyst injections embryos were cultured for 3 nights. On the day of the injection, groups of embryos were placed in drops of M2 medium and using a 20 µm diameter injection pipet (Origio, Inc.) 3-10 cells were injected under the zona or in non-compacted embryos in the inner space of the embryo. For the injection of blastocysts, approximately 10 cells were injected into the blastocoel cavity. In both cases, the injection was assisted by an XY Clone laser (Hamilton Thorne, Inc.). About 20 blastocysts were subsequently transferred to each recipient female; the day of injection was considered as 2.5 dpc. Fetuses were collected at 9.5-12.5 dpc for further analyses. As previously, all animal experiments were performed in compliance with protocol # 1031-088-16 from the Committee on Animal Care at MIT. In addition, interspecies chimerism experiments were approved by the Embryonic Stem Cell Research Oversight (ESCRO) Committee at Whitehead Institute.

Human mitochondrial PCR assay

Genomic DNA was extracted from E9.5-12.5 injected mouse embryos. 25ng of genomic DNA per sample was used for qPCR analysis to measure the presence of human DNA. The existence of human DNA in embryos was determined by amplification of a human-specific mitochondrial (mtDNA) fragment. To correct for sample-to-sample variations in qPCR efficiency and errors in sample quantification the amplification of a specific ultra-conserved non-coding element (UCNE) was used as invariant endogenous control. All

samples were run in technical triplicates in a 384-well plate with Fast SYBR Green Master Mix (Life Technologies) using an ABI Prism 7900HT Sequence detection system (Life Technologies). Relative quantification ratios were calculated and determined by $R=2^{-\Delta\Delta Ct}$. For all experimental samples, a relative quantification (RQ) was measured in comparison to negative-control mouse DNA samples. In addition, for calibration, each assay contained the human:mouse cell serial dilutions. Additional details of this protocol are described in (Cohen et al., 2016).

qRT-PCR

Total RNA was isolated using the Rneasy Kit (QIAGEN) and reversed transcribed using the Superscript III First Strand Synthesis kit (Invitrogen). Quantitative RT-PCR analysis was performed in triplicate using the ABI 7900 HT system with FAST SYBR Green Master Mix (Applied Biosystems). Gene expression was normalized to a reference gene (RPLP0). Error bars represent the standard deviation (SD) of the mean of triplicate reactions. Primer sequences are included in the following primer table:

Primers used in this study:

**Gene**	**Primer sequence (5'- 3')**	**Application**
ZIC2-F	CCCTTCAAGGCCAAATACAA	RT-PCR
ZIC2-R	TGCATGTGCTTCTTCCTGTC	
STELLA-F	GTTACTGGGCGGAGTTCGTA	RT-PCR
STELLA-R	TGAAGTGGCTTGGTGTCTTG	
REX1-F	GGAATGTGGGAAAGCGTTCGT	RT-PCR
REX1-R	CCGTGTGGATGCGCACGT	
FUW-KLF2-F	GATTTTGCTGGGTTGGTTTTT	RT-PCR
FUW-KLF2-R	CCACATAGCGTAAAAGGAGCA	
FUW-NANOG-F	GCTGGGGAAGGCCTTAATGT	RT-PCR
FUW-NANOG-R	CCACATAGCGTAAAAGGAGCA	

GAPDH-F	CGAGATCCCTCCAAAATCAA	RT-PCR
GAPDH-R	ATCCACAGTCTTCTGGGTGG	
RPLP0-F	GCTTCCTGGAGGGTGTCC	RT-PCR
RPLP0-R	GGACTCGTTTGTACCCGTTG	

RNA-Seq analysis

To prepare RNA for sequencing, 1 million naive or primed human ESCs were trypsinized and purified from GFP-labeled or non-fluorescent MEFs using the FACSAria (Beckton-Dickinson) prior to lysis and RNA extraction. Samples were homogenized in 1 ml of TRIzol Reagent (Life Technologies, 15596-026), purified using the mirVANA miRNA isolation kit (Ambion, AM1560) following the manufacturer's instructions and re-suspended in 100 µl nuclease-free water (Ambion, AM9938). Reads were mapped to the human genome (hg19) using TopHat (v2.0.11) in sensitive mode (the exact parameters are: tophat --library-type fr-firststrand -g 1 --no-novel-juncs --no-novel-indels -G $gtf --transcriptome-index $ transcriptome --b2-sensitive -o $localdir $index $reads1 $reads2). Gene counts were generated using HTSeq-count. For repetitive sequences, an in-house curated version of the Repbase database was used (fragmented LTR and internal segments belonging to a single integrant were merged). TEs counts were generated using the multiBamCov tool from the bedtools software. TEs which did not have at least one sample with 20 reads (after normalizing for sequencing depth) were discarded from the analysis. TEs overlapping exons were also removed from the analysis. Normalisation for sequencing depth and differential gene expression analysis was performed using Voom (Law et al., 2014) as it has been implemented in the limma package of Bioconductor (Gentleman et al., 2004). A gene (or TE) was considered to be differentially expressed when the fold change between groups was bigger than 2 and the p-value was smaller than 0.05. A moderated paired t-test (as implemented in the limma package of R) was used to test significance. P-values were corrected for multiple testing using the Benjamini-Hochberg's method (Benjamini, 1995). Single cell RNA-Seq data was downloaded from GEO (Edgar et al., 2002) (GSE36552). Data was mapped and processed as explained above. When merging with in-house RNA-Seq, only TEs which were expressed in both datasets were used. To be considered expressed, the TE needed to have at least as many reads across all samples as samples existed in each dataset.

Transposon expression profiling was performed on the following primed cell lines: male WIBR1 hESCs (Lengner et al., 2010), male NPC 1.1#4 and NPC 1.1#13 hiPSCs

(Maetzel et al., 2014), and two female primed WIBR2 hESC clones independently targeted with fluorescent reporters at both alleles of MECP2 (29M-GP26-TN9 = GFP-positive in primed state; 29M-TN3-GP8 = tdTomato-positive in primed state). Transposon expression profiling was performed on the following naive cell lines: male WIN1 hESCs derived directly from the human embryo (Theunissen et al., 2014) and maintained in 5i/L/A for 19 passages, double-color WIBR2 MECP2 reporter cells derived in 5i/L/A for 10 passages, WIBR2 and WIBR3 naive hESCs derived in 4i/L/A for 4 passages and two clones (#12 and #16) of WIBR3 OCT4-ΔPE-GFP-positive naive cells maintained with DOX-inducible KLF2 and NANOG transgenes in t2i/L/DOX+RI. In addition, we performed transposon profiling on three additional lines cultured in KSR-based NHSM media on MEFs (Gafni et al., 2013): WIBR2 ESCs cultured in NHSM for 13 passages, C1 AAVS1-GFP iPSCs cultured in NHSM for 19 passages and LIS2 ESCs cultured in NHSM for 38 passages. C1 AAVS1-GFP iPSCs (P9 NHSM) and LIS2 ESCs (P35 NHSM) were shared by Dr. Jacob Hanna's lab (Weizmann Institute, Rehovot, Israel) and maintained in NHSM for 10 and 3 additional passages, respectively, before FACS purification to remove MEFs and RNA isolation.

Correspondence between naive/primed ESCs and single cell expression data from human embryonic stages

For every cell state we perform a statistical test to find the genes (or TEs) that have a different expression level compared to the other cell states (Yan et al., 2013). For this we use a moderated F-test (comparing the interest group against every other) as implemented in the limma package of Bioconductor. For a gene (or TE) to be selected as expressed in a specific cell state it needs to have a significant p-value (<0.05 after adjusting for multiple testing with the Benjamini and Hochberg method) and an average fold change respective to the other cell states greater than 10. Note that with this approach a gene (or TE) can be marked as expressed in more than one cell state.

Once we have the genes (or TEs) that are expressed in a specific cell state we ask if those genes are more expressed in primed or in naive. For that we see how many of the genes are up (or down) regulated in the primed/naive pairwise comparison. For a gene

to be considered differentially expressed it needs to have a p-value (after multiple testing correction with the Benjamini and Hochberg method) lower than 0.05 and a fold change greater than 2.

Genome-wide SNP Genotyping

Affymetrix human SNP array 6.0 was used to interrogate SNP genotypes of the WIBR2 and WIBR3 cell lines. For WIBR2, data were generated by hybridizing genomic DNA to the array according to manufacturer’s instructions. For WIBR3, data were obtained from a previous study (Soldner et al., 2011) deposited in GEO (GSM738141) that analyzed copy number variations (CNVs) rather than SNP genotypes. Array intensity data were analyzed by Affymetrix Genotyping Console. The BIRDSEED V2 algorithm and the default confidence threshold (0.1) were used for genotyping call. Five additional arrays from the same previous study (Soldner et al., 2011) generated from similar cell lines using the same platform (GSM738137, GSM738138, GSM738139, GSM738140, and GSM738142) were included in the analysis to boost sensitivity of the genotyping call.

Quantification of allele-specific gene expression

Genotype information for SNPs on X chromosome was obtained using the Affymetrix human SNP array 6.0 as described above. SNPs found to be heterozygous in WIBR2, i.e. having an “AB” genotype, were selected for further analysis (5044 such SNPs were found on X chromosome). To determine allele-specific gene expression, 2x100 paired-end sequencing reads of RNAs isolated from WIBR2 single color $MECP2^{GFP\text{-}ON/Tom\text{-}OFF}$ or $MECP2^{GFP\text{-}OFF/Tom\text{-}ON}$ primed cells and double color naive cells derived in 5i/L/A and maintained in 5i/L/A or 4i/L/A were mapped to the human reference genome hg38 using STAR (Dobin et al., 2013). Read depths of the two different alleles “A” and “B” at each of the SNP sites were analyzed for the RNA-seq data using samtools mpileup (Li, 2011). A stringent filter, requiring a total read depth of at least 10 in all samples was applied to select SNPs that can be used to reliably derive allelic expression information. Relative allelic expression level was calculated as number of reads originating from a

particular allele divided by total number of reads. Genes reported to escape X inactivation are labeled in red: CD99 and ZBED1 are positioned in the PAR region, while PIR is listed as Discordant in the consensus definition (Balaton et al., 2015).

Quantification of total X-linked gene expression

RNA-seq paired-end reads of 100 nt were aligned to human reference genome hg38 using STAR (Dobin et al., 2013). Gene expression was quantified using cufflinks (Roberts et al., 2011). Genes on X chromosome having an expression level of greater than one FPKM in at least one sample were selected for analysis. Selected genes were arranged according to their order on the X chromosome. For each gene, the average of the FPKM value in male naive samples was used as a baseline; Log2(FoldChange) was calculated for each individual sample compared to the baseline. Medians of the log2(FoldChange) values of a moving window of 50 genes were plotted. The quantification of total X-linked gene expression levels was performed using single-color MECP2 reporter female primed lines (n=2), male primed lines maintained in the same growth conditions (n=3), double-color MECP2 reporter naive cells obtained by conversion in 5i/L/A and maintenance in 5i/L/A, 4i/L/A or 4i/L/A+CHIR99021 for 10 passages (n=3) and naive male embryo-derived WIN1 ESCs maintained in 5i/L/A or 4i/L/A (n=2). Note that in this analysis all samples were normalized to the average expression in male naive samples, whereas in our previous study (Theunissen et al., 2014) we performed a similar analysis using male primed cells as baseline.

ChIP-Seq

Cells were cross-linked for 10 minutes at room temperature by the addition of one-tenth of the volume of 11% formaldehyde solution (11% Formaldehyde Sigma 2525459, 50mM HEPES pH 7.5, 100 mM NaCl, 1 mM EDTA pH 8.0, 0.5 mM EGTA pH 8.0) to the growth media followed by quenching with 100 mM glycine. Cells were washed twice with PBS, then the supernatant was aspirated and the cell pellet was flash frozen in liquid nitrogen.

Pellets were lysed, resuspended in 1mL of LB1 on ice for 10min (50 mM HEPES-KOH pH 7.4, 140 mM NaCl, 1 mM EDTA, 0.5 mM EGTA, 10% Glycerol, 0.5% NP40, 0.25% Tx100, protease inhibitors), then after centrifugation resuspend in LB2 on ice for 10min (10 mM Tris pH 8.0, 200 mM NaCl, 1 mM EDTA, 0.5 mM EGTA and protease inhibitors). After centrifugation, resuspend in LB3 (10 mM Tris pH 8.0, 200 mM NaCl, 1 mM EDTA, 0.5 mM EGTA, 0.1% NaDOC, 0.1% SDS and protease inhibitors) and sonicated (Covaris settings: 5% duty, 200 cycle, 140 PIP, 60 min), yielding genomic DNA fragments with a bulk size of 100-300 bp.

Coating of the beads with the specific antibodies (KAP1 ab10483 and H3K9me3 Diagenode pAb-056-050) was carried out during the day at 4°C, then chromatin was added overnight at 4°C. Subsequently, washes were performed with 3x Wash Buffer (100 mM Tris pH 8.0, 1 mM EDTA, 0.5 mM EGTA, 500 mM LiCl, 1% NP40, 1% NaDOC) and 1 with TE buffer. Final DNA was purified with Qiagen Elute Column.

Up to 10 nanograms of ChIPed DNA or input DNA (Input) were prepared for sequencing. Library was quality checked by DNA high sensitivity chip (Agilent). Quality controlled samples were then quantified by picogreen (Qubit® 2.0 Fluorometer,Invitrogen). Cluster amplification and following sequencing steps strictly followed the Illumina standard protocol.

Sequenced reads were de-multiplexed to attribute each read to a DNA sample and then aligned to reference human genome hg19 with bowtie2 (v2.2.4 with parameters -k 1 --end-to-end). PCR duplicates removal (MarkDuplicates using picard tools v1.8 and parameters: VALIDATION_STRINGENCY=LENIENT REMOVE_DUPLICATES=true), samples were downsampled (DownsampleSam using picard tools v1.8) to the lowest dataset count for H3K9me3 and KAP1 (app. 23 millions reads) and for H3K4me3 used from (Theunissen et al., 2014) (app. 9 millions reads).

Heatmaps and profile averages were calculated using ngs.plot R package (Shen et al., 2014) over 5/20kb windows around the 5' of repeat elements from BAM files (Repbase

X version), (Parameters used with ngs.plot -SC global -I 0 -L 2500 -MQ0 -RB 0.05). Datasets are normalized to their respective Input DNA sample. Screenshots were made with bigwig file produce with MACS1.4 and every scale has been adjusted to the same value.

MethylC-Seq library construction

Primed methylomes were mapped in WIBR1, WIBR2, WIBR3, WIBR3 AAVS1-GFP and previously analyzed H9 ESCs (Takashima et al., 2014). Naive methylomes were mapped in WIBR3 (AAVS1-GFP) ESCs in 5i/L/A (P21), WIN1 naive embryo-derived ESCs in 5i/L/A (P15), WIBR2 and WIBR3 ESCs in 4i/L/A (P4), two lines of WIBR3 OCT4-ΔPE-GFP DOX-inducible KLF2+NANOG naive cells (#12 and #16), and previously analyzed H9 naive ESCs maintained in t2i/L+PKCi (Takashima et al., 2014). Additional methylomes were mapped in re-primed cells (P12 and P19, respectively) obtained from WIBR3 4i/L/A naive cells and WIBR3 (AAVS1-GFP) 5i/L/A naive cells and neural precursors derived from WIBR3 4i/L/A re-primed cells.

To prepare DNA for MethylC-Seq, 2 million naive or primed human ESCs were trypsinized and purified from GFP-labeled or non-fluorescent MEFs using the FACSAria (Beckton-Dickinson). Genomic DNA was extracted using the DNeasy Blood and Tissue Kit (Qiagen, Valencia, CA). 2 µg of genomic DNA was spiked with 10 ng unmethylated cl857 Sam7 Lambda DNA (Promega, Madison, WI). The DNA was fragmented with a Covaris S2 (Covaris, Woburn, MA) to 150-200 bp, followed by end repair and addition of a 3’ A base. Cytosine-methylated adapters provided by Illumina (Illumina, San Diego, CA) were ligated to the sonicated DNA at 16˚C for 16 hours with T4 DNA ligase (New England Biolabs). Adapter-ligated DNA was isolated by two rounds of purification with AMPure XP beads (Beckman Coulter Genomics, Danvers, MA). Adapter-ligated DNA (≤450 ng) was subjected to sodium bisulfite conversion using the MethylCode kit (Life Technologies, Carlsbad, CA) as per manufacturer’s instructions. The bisulfite-converted, adapter-ligated DNA molecules were enriched by 4 cycles of PCR with the following reaction composition: 25 µL of Kapa HiFi Hotstart Uracil+ Readymix (Kapa Biosystems, Woburn, MA) and 5 µl TruSeq PCR Primer Mix (Illumina) (50 µl final). The

thermocycling parameters were: 95˚C 2 min, 98˚C 30 sec, then 4 cycles of 98˚C 15 sec, 60˚C 30 sec and 72˚C 4 min, ending with one 72˚C 10 min step. The reaction products were purified using AMPure XP beads. Up to two separate PCR reactions were performed on subsets of the adapter-ligated, bisulfite-converted DNA, yielding up to two independent libraries from the same biological sample.

MethylC-Seq data processing

MethylC-Seq data were processed as described in Schultz et al. (Schultz et al., 2015) with some modification. Briefly, adapter sequences on MethylC-Seq reads were trimmed using Cutadapt (version 1.2.1) (Martin, 2011). Next, cytosines (Cs) in the trimmed reads were computationally converted to thymines (Ts). The converted reads were mapped twice, to the forward strand of a converted Watson strand reference and the reversed strand of a converted Crick strand reference. A converted reference is created by replacing all cytosines with thymines (Watson strand) or by replacing all guanines with adenines (Crick strand). For mapping single-end reads, Bowtie 2 (version 2.2.5) (Langmead and Salzberg, 2012) was used along with default options. For aligning paired-end reads, bowtie 2 (version 2.2.5) was used with following options: “-k 2 -q --no-mixed --no-discordant --maxins 1000 --score-min L,0,-0.2”. Reads were mapped to hg19 reference genome. Any read that mapped to multiple locations was removed and one read from each starting location on each strand from each library was kept (i.e., clonal reads were removed).

To identify the sites showing evidence of methylation (i.e. methylated sites), we first counted the number of reads that supported methylation and the number of reads covering. Then, binomial test was performed on these counts to test whether the methylation counts are beyond bisulfite non-conversion events. In the test, the probability of observing methylation in null hypothesis, which is that methylation observed comes from incomplete bisulfite conversion, equals the non-conversion rate, which was determined by computing the fraction of methylated reads in the lambda genome which was spiked in during library construction. For samples (Naive H9 and Primed H9 ESCs from Takashima et al. (Takashima et al., 2014) without unmethylated

DNA spiked in, the genome-wide non-CG methylation level was used as an estimate of non-conversion rate. The false discovery rate (FDR) was computed using Benjamini-Hochberg method and p-value cutoff was calculated given 1% FDR. Considering that the p-value distributions for each methylation context are different, this procedure was applied to each three nucleotide context independently (e.g., a p-value cutoff was calculated for CAC cytosines). AnnoJ browser (Wang et al., 2013) was used for visualization of MethylC-Seq data.

Reduced representation bisulfite sequencing (RRBS) data processing

RRBS reads were first trimmed using trim_galore [http://www.bioinformatics.babraham.ac.uk/projects/trim_galore/] with options "--rrbs -paired -trim1". Then, trimmed reads were mapped to hg19 human reference genome and lambda genome together by bowtie2 using the bismark pipeline (--bowtie2 -X 1000). Bisulfite non-conversion rate was estimated in the same way as in MethylC-seq data. In Figure S4A only CpG sites covered in at least one of the two RRBS experiments (8 cells and morula) (Guo et al., 2014) were included, i.e. CpG sites covered in MethylC-seq but not in RRBS were excluded.

Calculation of methylation levels

Methylation level for single site is defined as the fraction of reads supporting methlyation out of reads that cover the site subtracting bisulfite non-conversion rate. For region with multiple sites, methylation level is defined as weighted methylation (Schultz et al., 2012) subtracting bisulfite non-conversion rate. CpG methylation level is also called as mCpG/CpG, which non-CpG methylation level is named as mCpH/CpH (where H = A, C or T).

Methylation levels across various genomic features

Figures S4C and S4D show the methylation levels across multiple genomic features. Exon and intron annotation was based on GENCODE (Harrow et al., 2012) annotation

(Release 19). Promoters are defined as +/- 1kb regions around transcription start sites of all genes from GENCODE annotation. CpG island (CGI) coordinates were downloaded from UCSC genome browser (Dec 1st, 2015). Enhancer center list was downloaded from Xie et al. (Xie et al., 2013). For each enhancer center, we extended 1kb on each side and then liftover the coordinates (hg18) to hg19 reference. Repeat annotation was obtained from Repbase. For Figure S4C-D, only regions that were covered in all samples were included in the calculation.

Methylation of transposable elements (TEs)

Figure 4C-E compared the methylation of overexpressed TEs with non-overexpressed TEs from the same family in naive versus primed cells or vice versa. TEs showing significantly increased expression were considered as overexpressed copies (1% FDR, See “RNA-Seq analysis” for details). Other integrants in the same family were used as control. Only integrants that were covered in all samples were included.

Analysis of imprinted Differentially Methylated Regions (DMRs)

A list of candidate imprinted DMRs was obtained from Table 1 in Court et al. (Court et al., 2014), excluding placenta-specific imprinted DMRs. In total, 50 candidate imprinted DMRs were included. For analysis on samples related to 4i/L/A condition (Figure 5B and C; Figure S5A, C and E), we filtered out imprinted DMRs with extreme CpG methylation level (less than 0.3 or greater than 0.7) in primed WIBR3, since imprinted DMRs should have an intermediate methylation level (here defined as CpG methylation level between 0.3 and 0.7). In total, 26 imprinted DMRs were included in more advanced analyses. For analysis on samples related to 5i/L/A condition (Figure 5B and D; Figure S5B, D and F), we filtered out imprinted DMRs with extreme CpG methylation level in the AAVS1-GFP targeted subclone of primed WIBR3, resulting in 28 imprinted DMRs. In Figure 5B, “Erased” is defined as regions where the intermediate methylation in primed WIBR3 (or AAVS1-GFP targeted subclone) becomes hypomethylated (mCpG/CpG < 0.3) in both naive and re-primed cells.

For analysis of imprinted DMRs in primed H9 ESCs from Takashima et al. (Takashima et al., 2014), we started with the same 50 candidate imprinted DMRs and filtered out regions that were not intermediately methylated in primed H9, i.e. mCpG/CpG is either greater than 0.7 or less than 0.3. After filtering, 31 imprinted DMRs remained. Out of the 31 regions, 24 regions showed less than 0.3 in CpG methylation level in naive H9 ESCs. Therefore, in total, 24 out 31 imprinted DMRs in primed H9 were lost in naive H9 ESCs using the conditions described in Takashima et al. (2014).

To plot the methylation changes in imprinted DMRs and flanking regions (Figure S5A and B), each imprinted DMR along with its upstream 1kb region and downstream 1kb region were equally divided into 10 bins, respectively (30 bins in total). Next, we calculated and plotted the CpG methylation level of each bin. In Figure S5C and D, instead of CpG methylation levels, we calculated the $\log_2$ fold change of CpG methylation levels compared to the parental primed line. Specifically, for each bin, we calculated the ratio of CpG methylation level in one specific sample to the CpG methylation level of the corresponding bin in the parental primed line. In Figure S5E and F, for each sample, we plotted the distribution of the $\log_2$ fold change of bins in imprinted DMRs and flanking regions respectively. The boxplots clearly showed that in naive cells, bins in both imprinted DMRs and flanking regions were generally hypomethylated compared to the parental primed cells, while the methylation decrease in imprinted DMRs was greater. In re-primed and neural precursor cells (NPCs), the bins in flanking regions were able to restore methylation to similar levels as in the parental primed cells, but methylation in bins in imprinted DMRs failed to fully restore.

Analysis of allelic CpG methylation in imprinted DMRs

To decode allelic methylation patterns, MethylC-seq reads were first assigned to alleles based on the overlaps between basecalls on reads and WIBR3 heterozygous SNPs on the genome. Reads overlapping multiple heterozygous SNPs were discarded. Bisulfite conversion was taken into account:

- When distributing reads mapped to Watson strand to alleles, we only considered non C-T SNPs (i.e. we ignored heterozygous SNPs that have C and T as alleles).

Then, both C and T basecalls on reads are both considered as matched to Cs on the genome because under bisulfite conversion, they correspond to methylated and unmethylated cytosines respectively.

- Similarly, for reads mapped to crick strand, only non G-A SNPs were used to determinate their original allele and consider both G and A basecalls on reads as matches to Gs on the genome.

Across all CpGs within the 26 primed WIBR3 imprinted DMRs, only two CpGs (chr15:25,069,480−25,069,481) were found to be covered by reads that could be assigned to alleles and at least 5 reads in each allele of this CpG pair in all samples included in the allelic methylation analysis: WIBR3 4i/L/A, WIBR3 5i/L/A, Re-primed WIBR3 4i/L/A, Re-primed WIBR3 5i/L/A, NPC Re-primed WIBR3 4i/L/A and Primed WIBR3. Then, we calculated the allelic CpG methylation difference (Figure S5G), defined as the CpG methylation difference between alleles.

CpG methylation states of X chromosome

In Figure 6A, CGI coordinates were downloaded from UCSC genome browser and promoters are +/- 1kb regions around transcription start sties of X-linked genes. Figure 6A and Figure S6A show the CGIs with at least 1bp overlap with promoter. In Figure 6B and Figure S6B, promoters that do not overlap any CGI are shown. In this analysis, gene annotation from UCSC genome browser was used to be consistent with the annotation used in RNA-Seq analysis. In the lower panel of Figure 6B, random 2kb bins were chosen from regions outside CGI and non-CGI promoters.

II. Supplemental Figure Legends

Figure S1. Titration of GSK3 inhibitor IM12 and correspondence of naive and primed cells to human embryonic stages by gene or TE expression [related to Figures 1-2]

(**A**) Phase images (Top) and flow cytometric analysis (Bottom) of the proportion of OCT4-ΔPE-GFP+ cells obtained after withdrawal of DOX and culture for 9 passages at low density splitting (1:10) in four distinct growth conditions: 2i/L, 5i/L/A, t5i/L/A (4i/L/A + 0.5 µM IM12) and 4i/L/A (-IM12). Wild-type WIBR3 and WIBR3 OCT4-ΔPE-GFP primed cells are included for comparison.

(**B**) Quantification of the cell number after two passages at low density splitting (1:10) following DOX withdrawal and expansion in 5i/L/A, t5i/L/A (4i/L/A + 0.5 µM IM12) and 4i/L/A (-IM12). Note that reduction or removal of the GSK3 inhibitor IM12 enhances the proliferation of naive human cells in a dose-dependent manner. Error bars indicate ± 1 SD.

(**C**) Quantitative gene expression analysis for exogenous *KLF2*, exogenous *NANOG* and endogenous *ZIC2*, *REX1* and *STELLA* in primed human ESCs and naive human ESCs maintained in 2i/L/DOX, t5i/L/A (4i/L/A + 0.5 µM IM12) and 4i/L/A. Error bars indicate ± 1 SD.

(**D-E**) Dotplots showing the expression of human embryo stage-specific genes (**D**) or TEs (**E**) in naive and primed human ESCs. Using single cell RNA-Seq data from early human embryos (Yan et al., 2013), a statistical test was performed to find the genes (or TEs) that have a different expression level for every stage of human embryonic development compared to the other stages. Stage-specific genes (or TEs) that are upregulated (p-value < 0.05, fold change 2) in naive or primed cells are indicated in orange and blue, respectively, while genes (or TEs) that did not change expression are indicated in grey. The naive samples include all three conditions of naive cells that we examined in the PCA analyses shown in Figure 2A-B (i.e. 5i/L/A, 4i/L/A and t2i/L/DOX+RI). See Supplemental Experimental Procedures for details of the analysis.

Figure S2. Evaluating the transposcriptome in alternative conditions for naive human pluripotency [related to Figures 1-2]

(**A**) RNA-Seq quantification of LTR7 integrants used as naive-like reporters by Izsvák and colleagues (Wang et al., 2014) in naive or primed ESCs. The naive samples include all three conditions of naive cells that we examined in the PCA analyses shown in Figure 2A-B (i.e. 5i/L/A, 4i/L/A and t2i/L/DOX+RI).

(**B**) Correspondence between gene expression (Left) or TE expression (Right) in naive/primed ESCs described by Smith and colleagues (Takashima et al., 2014) and single cell human embryonic stages (Yan et al., 2013). For every stage of human embryonic development a statistical test was performed to find the genes (or TEs) that have a different expression level

compared to the other stages. The proportions of developmental stage-specific genes (or TEs) that are upregulated (p-value < 0.05, fold change 2) in t2i/L+Gö or primed cells are indicated in orange and blue, respectively, while genes (or TEs) that did not change expression are indicated in grey. See Supplemental Experimental Procedures for details of the analysis.

(**C**) Correspondence between gene expression (Left) or TE expression (Right) in naive ESCs cultured in KSR-based NHSM conditions as described by Hanna and colleagues (Gafni et al., 2013) and single cell human embryonic stages (Yan et al., 2013). For every stage of human embryonic development a statistical test was performed to find the genes (or TEs) that have a different expression level compared to the other stages. The proportions of developmental stage-specific genes (or TEs) that are upregulated (p-value < 0.05, fold change 2) in NHSM or primed cells are indicated in orange and blue, respectively, while genes (or TEs) that did not change expression are indicated in grey. See Supplemental Experimental Procedures for details of the analysis.

Figure S3. Expression of trophectoderm-associated transcription factors in naive human ESCs [related to Figure 2]

RNA-Seq quantification of 8 transcription factors that are upregulated in naive human ESCs and were previously reported to be overexpressed in trophectoderm and placenta compared to conventional human ESCs (Bai et al., 2012).

Figure S4. Genome-wide DNA demethylation in naive human ESCs [related to Figure 4]

(**A**) Genome-wide CpG methylation levels of all samples using regions covered by Reduced Representation Bisulfite Sequencing (RRBS) to enable incorporation of RRBS-generated 8 cell and morula methylome datasets (Guo et al., 2014).

(**B**) Genome-wide non-CpG methylation levels in naive and primed human ESCs.

(**C-D**) Two bar charts showing the (**C**) CpG and (**D**) non-CpG methylation levels of various genomic features in all samples. The order of samples in each bar subplot is the same as shown in the figure legend on the left.

Figure S5. Disruption of imprinted DMRs in naive human ESCs [related to Figure 5]

(**A-B**) Heatmaps showing the CpG methylation levels of imprinted DMRs and the flanking regions (+/- 1kb) in WIBR3 primed ESCs before and after conversion in 4i/L/A (**A**) or WIBR3 (AAVS1-GFP targeted subclone) ESCs before and after conversion in 5i/L/A (**B**) together with re-primed derivatives and differentiated NPC (neural progenitor cells). Upstream and downstream flanking regions were divided into 10 100bp bins respectively while imprinted DMRs were equally divided into 10 bins. Imprinted DMRs highlighted in blue are ones that do not lose DNA methylation in re-primed cells but it is unclear whether the allelic methylation patterns in imprinted DMRs are maintained.

(**C-D**) Heatmaps showing CpG methylation levels $\log_2$ fold change of imprinted DMRs and the flanking regions (+/- 1kb) in naive ESCs converted in 4i/L/A and their re-primed and neural derivatives compared to the parental WIBR3 primed ESCs (**C**) or naive ESCs converted in 5i/L/A and their re-primed derivatives compared to the parental WIBR3 (AAVS1-GFP targeted subclone) primed ESCs (**D**).

(**E**) Boxplot displaying the CpG methylation levels $\log_2$ fold change of bins within imprinted DMRs (blue) and within flanking regions (grey) in 4i/L/A and derivative re-primed cells and neural precursors. Similar to (C), $\log_2$ fold change was calculated compared to the parental WIBR3 primed ESCs. Bold line in the center of box indicates the median and notch around it shows the confidence interval (calculated by the “boxplot” function in R). Whiskers indicate maximum or minimum values within 1.5 interquartile range to upper (or lower) quartile.

(**F**) Boxplot displaying the CpG methylation levels $\log_2$ fold change of bins within imprinted DMRs (blue) and within flanking regions (grey) in 5i/L/A and derivative re-primed cells. Similar to (D), $\log_2$ fold change was calculated compared to the parental WIBR3 (AAVS1-GFP targeted) primed ESCs. Bold line in the center of box indicates the median and notch around it shows the confidence interval (calculated by the “boxplot” function in R). Whiskers indicate maximum or minimum values within 1.5 interquartile range to upper (or lower) quartile.

(**G**) Allelic CpG methylation levels of region with two CpGs (chr15:25,069,480-25,069,481), which is within imprinted DMR near *SNRPN* gene.

Figure S6. Methylation status of the X chromosome and double-color MECP2 reporter system [related to Figure 6]

(**A-C**) Boxplots showing CpG methylation levels at (**A**) X-linked promoter CpG islands (CGIs), (**B**) non-CGI promoter regions and (**C**) random 2 kb bins in naive and primed human ESCs. The random 2kb bins do not overlap any CGIs or non-CG promoters. Promoters are defined as +/- 1kb regions around transcription start sites. Bold line in the center of box indicates the median and notch around it shows the confidence interval (calculated by the “boxplot” function in R). Whiskers indicate maximum or minimum values within 1.5 interquartile range to upper (or lower) quartile.

(**D**) Strategy for establishing a dual color reporter system of X chromosome status in human ESCs by TALEN-mediated targeting of the two alleles of MECP2 with tdTomato and GFP sequences.

(**E**) Southern blot confirming the generation of double-targeted MECP2 reporter cells with MECP2exon3-Tomato-PGK-NEO and MECP2exon3-GFP-PGK-PURO donor vectors.

(**F**) Phase and fluorescence images of $MECP2^{GFP\text{-}ON/Tom\text{-}OFF}$ primed cells in hESM, and after conversion to the naive state in 5i/L/A and 4i/L/A. Note that the maximum intensity of MECP2-GFP is significantly weaker than MECP2-tdTomato (see also Figure S6G) and is more readily discerned by fluorescence microscopy in dome-shaped naive colonies than flat primed colonies.

(**G**) Histograms reporting the levels of MECP2-GFP and MECP-tdTomato in $MECP2^{GFP\text{-}ON/Tom\text{-}OFF}$ primed cells in hESM, and after conversion to the naive state in 5i/L/A and 4i/L/A. Note that the maximum intensity of MECP2-GFP is significantly weaker than MECP2-tdTomato.

Figure S7. Further characterization of X chromosome status in naive human ESCs [related to Figure 6]

(**A**) Flow cytometric analysis showing the distribution of MECP2-GFP and MECP-tdTomato in $MECP2^{GFP\text{-}ON/Tom\text{-}OFF}$ primed cells in hESM, and after conversion to the naive state in 5i/L/A and 4i/L/A.

(**B**) Quantification of total X-linked gene expression using a moving median plot in single-color MECP2 reporter female primed lines (n=2, yellow), male primed lines maintained in the same growth conditions (n=3, red), double-color MECP2 reporter naive cells obtained by conversion in 5i/L/A and maintenance in 5i/L/A, 4i/L/A or 4i/L/A+CHIR99021 for 10 passages (n=3, blue) and naive male embryo-derived WIN1 ESCs in 5i/L/A or 4i/L/A (n=2, green). For all samples,

the X-linked expression level is normalized to that of male naive cells. Note that in our previous study (Theunissen et al., 2014), a similar analysis was performed using male primed cells as the baseline. This graph indicates that naive and primed human ESCs have different transcriptional outputs.

(**C**) Comparison of total X-linked gene expression in double-color MECP2 reporter naive cells obtained by conversion in 5i/L/A and maintenance in 5i/L/A, 4i/L/A or 4i/L/A+CHIR99021 for 10 passages (n=3, red) and, as controls in the same cell state, naive male ESCs (WIN1) maintained in 5i/L/A or 4i/L/A (n=2, green). Note that female naive cells have slightly increased X-linked gene expression when compared to male naive control cells.

(**D**) Normalized levels of *XIST* expression in RNA-Seq analysis of MECP2 reporter single-color primed cells and double color naive cells obtained by conversion in 5i/L/A and maintained in 5i/L/A or 4i/LA for 10 passages. Male ESCs grown in the same conditions (primed and naive) were used for comparison.

III. Supplemental Table Legends

Supplemental Table 1. Mean expression counts for all TE integrants that yielded a minimum of 20 reads in at least one ESC sample **[related to Figure 1]**.

Supplemental Table 2. Top 10 naive TEs with highest fold change compared with primed ESCs **[related to Figure 1]**.

Supplemental Table 3. A summary of various statistics for methylomes included in this study **[related to Figure 4]**.

Supplemental Table 4. CpG methylation data on candidate imprinted DMRs **[related to Figure 5]**.

IV. Supplemental Figures

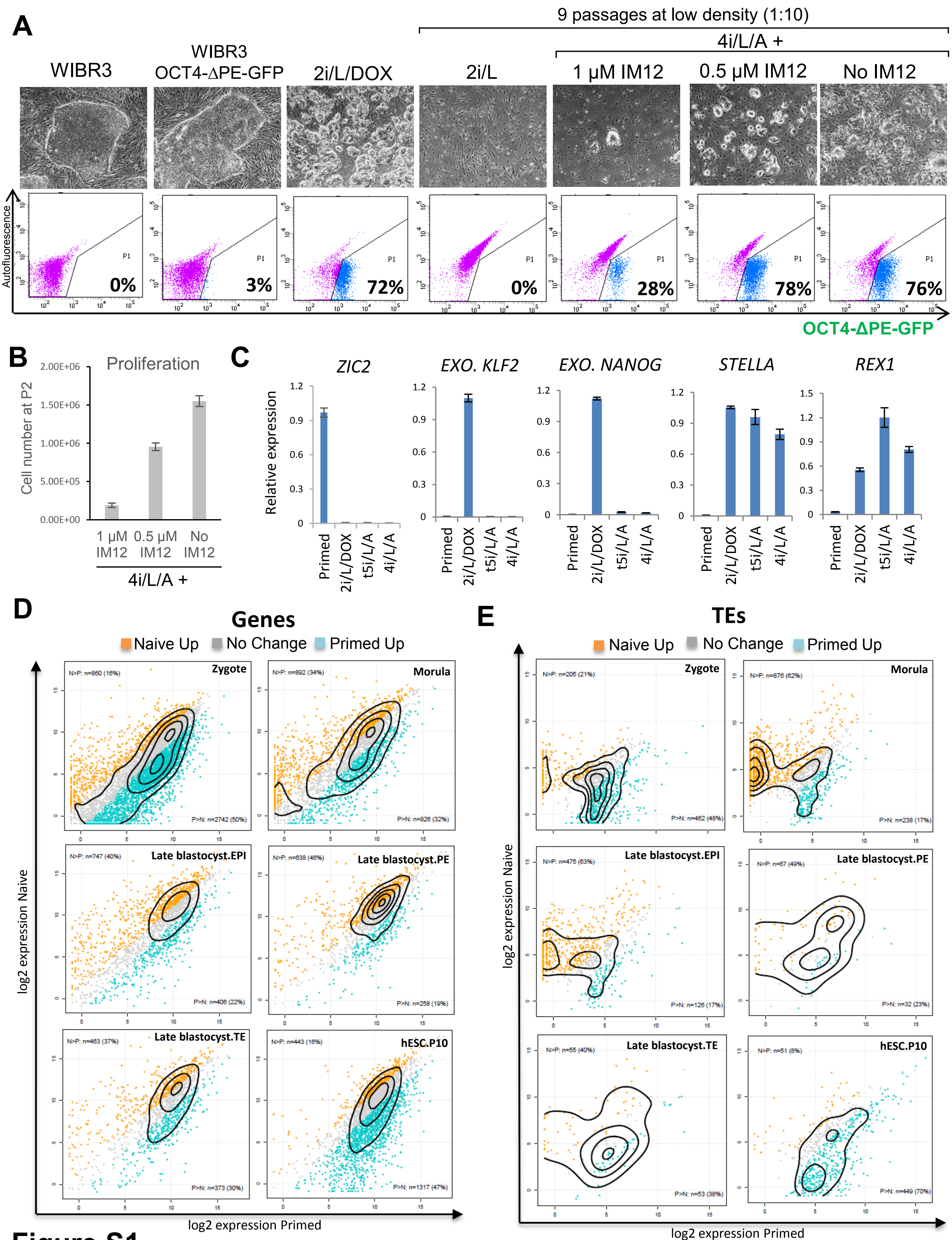
A
9 passages at low density (1:10)
4i/L/A +
WIBR3
WIBR3 OCT4-ΔPE-GFP
2i/L/DOX
2i/L
1 µM IM12
0.5 µM IM12
No IM12
Autofluorescence
0%
3%
72%
0%
28%
78%
76%
OCT4-ΔPE-GFP
B
Proliferation
Cell number at P2
1 µM IM12
0.5 µM IM12
No IM12
4i/L/A +
C
Relative expression
ZIC2
EXO. KLF2
EXO. NANOG
STELLA
REX1
Primed
2i/L/DOX
t5i/L/A
4i/L/A
D
Genes
Naive Up
No Change
Primed Up
Zygote
Morula
Late blastocyst.EPI
Late blastocyst.PE
Late blastocyst.TE
hESC.P10
log2 expression Naive
log2 expression Primed
E
TEs
Naive Up
No Change
Primed Up
Zygote
Morula
Late blastocyst.EPI
Late blastocyst.PE
Late blastocyst.TE
hESC.P10
log2 expression Naive
log2 expression Primed

Figure S1

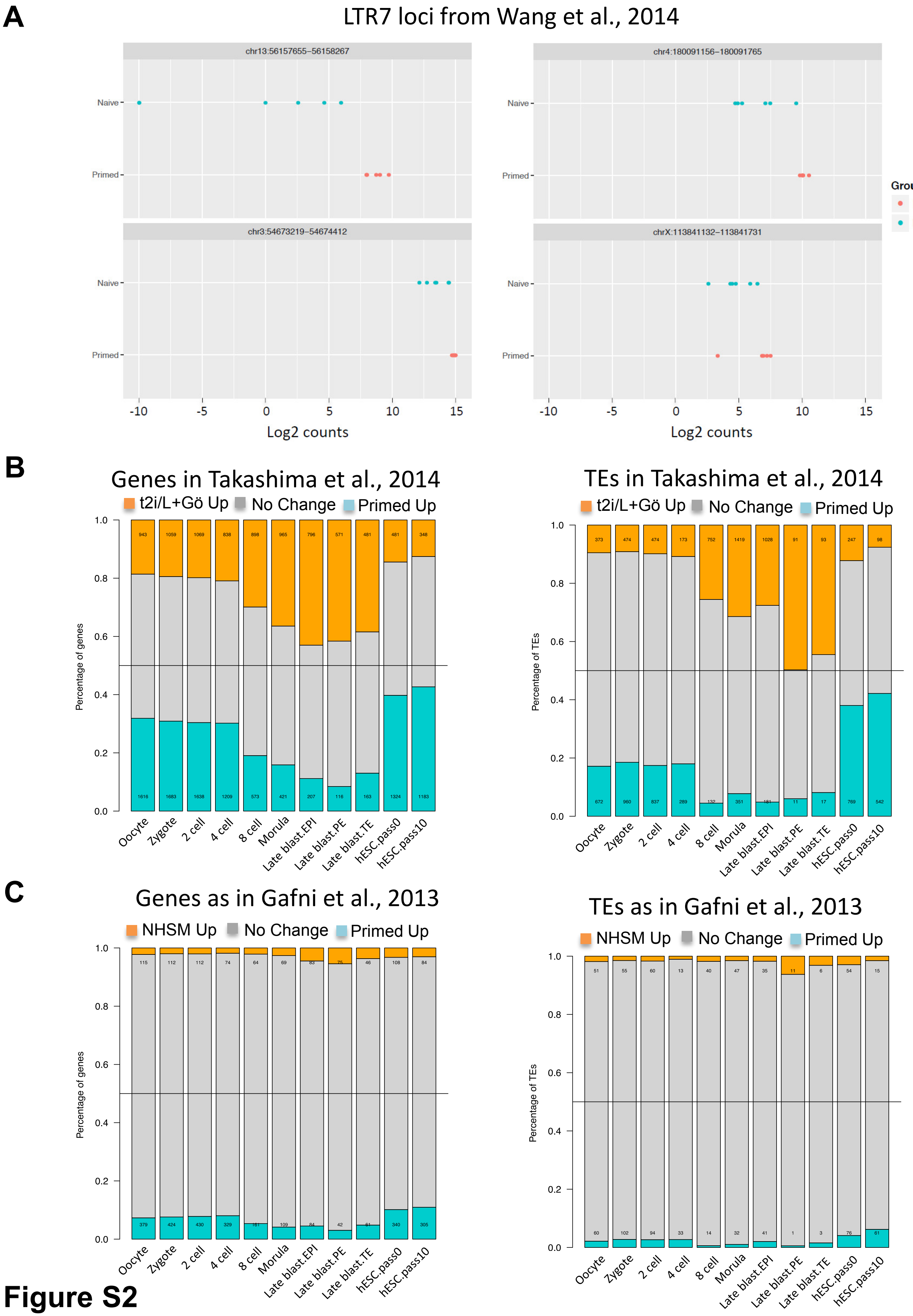
A
LTR7 loci from Wang et al., 2014
chr13:56157655–56158267
chr4:180091156–180091765
chr3:54673219–54674412
chrX:113841132–113841731
Naive
Primed
Group
Primed
Naive
-10 -5 0 5 10 15
Log2 counts
B
Genes in Takashima et al., 2014
TEs in Takashima et al., 2014
t2i/L+Gö Up
No Change
Primed Up
Percentage of genes
Percentage of TEs
0.0 0.2 0.4 0.6 0.8 1.0
943 1059 1069 838 898 965 796 571 481 481 348
1616 1683 1638 1209 573 421 207 116 163 1324 1183
373 474 474 173 752 1419 1028 91 93 247 98
672 960 837 289 132 351 181 11 17 769 542
Oocyte
Zygote
2 cell
4 cell
8 cell
Morula
Late blast.EPI
Late blast.PE
Late blast.TE
hESC.pass0
hESC.pass10
C
Genes as in Gafni et al., 2013
TEs as in Gafni et al., 2013
NHSM Up
No Change
Primed Up
115 112 112 74 64 69 83 75 46 108 84
379 424 430 329 161 109 84 42 61 340 305
51 55 60 13 40 47 35 11 6 54 15
60 102 94 33 14 32 41 1 3 76 61

Figure S2

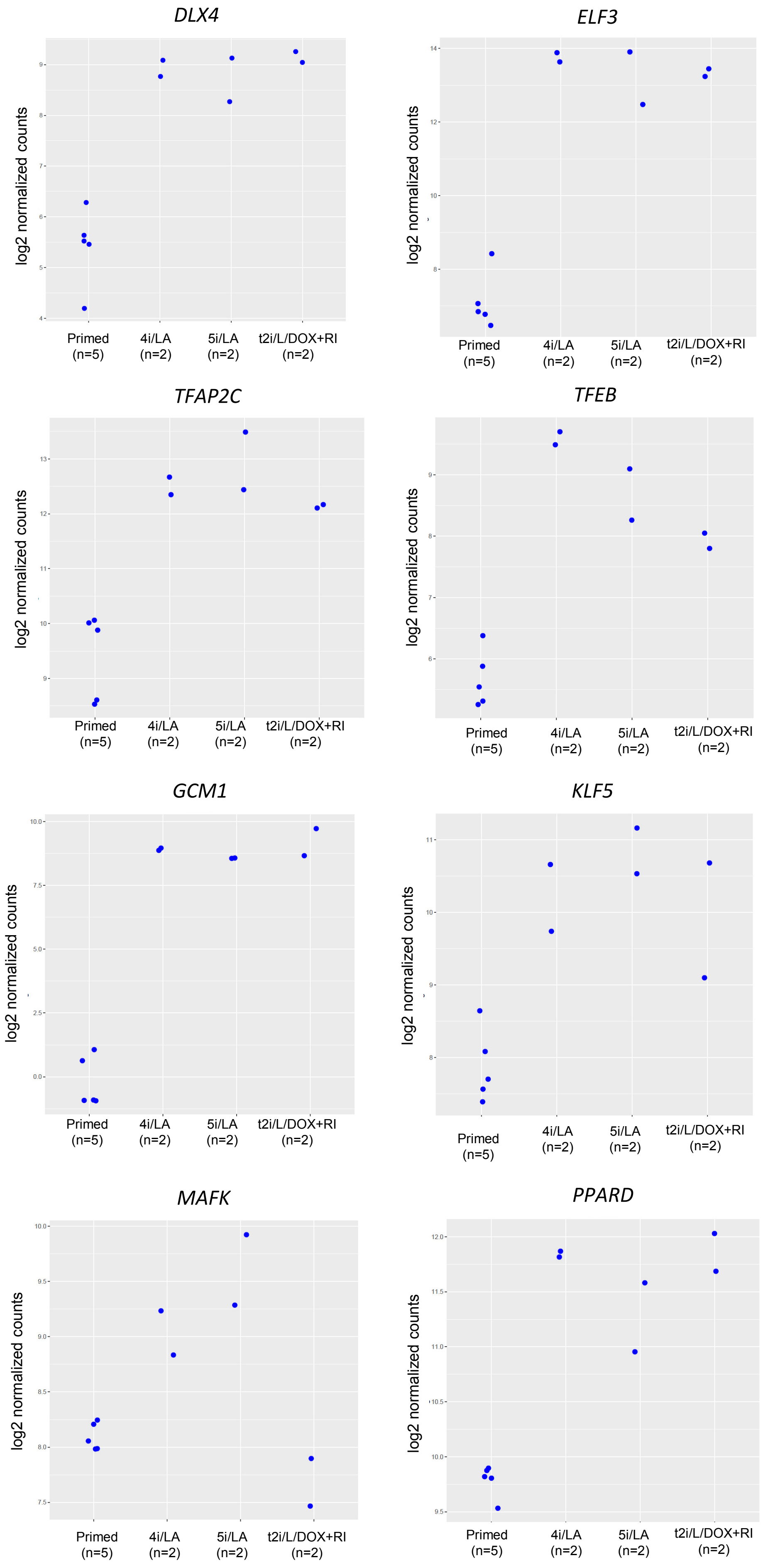

DLX4
ELF3
TFAP2C
TFEB
GCM1
KLF5
MAFK
PPARD
log2 normalized counts
Primed (n=5)
4i/LA (n=2)
5i/LA (n=2)
t2i/L/DOX+RI (n=2)

Figure S3

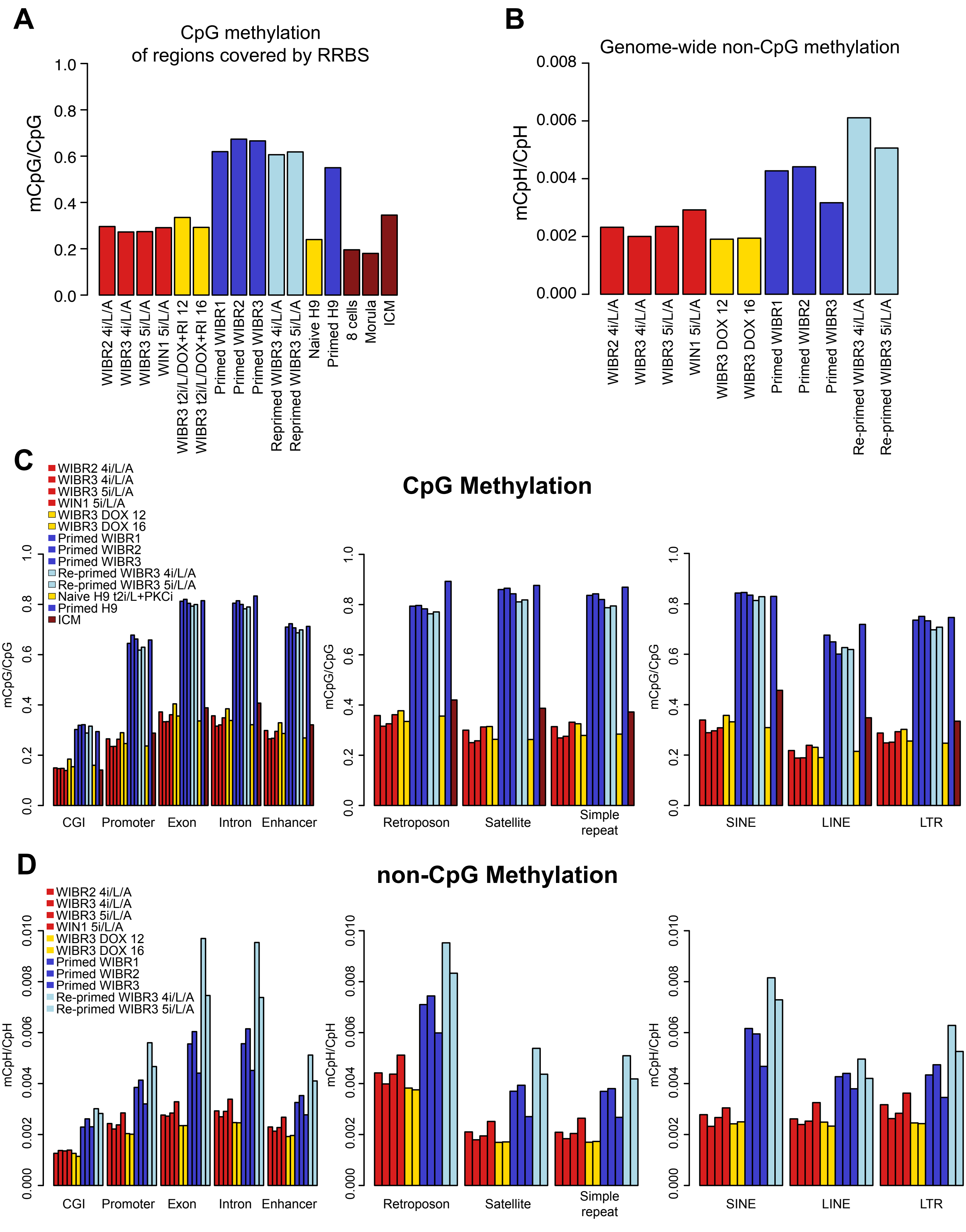
A
CpG methylation
of regions covered by RRBS
mCpG/CpG
1.0
0.8
0.6
0.4
0.2
0.0
WIBR2 4i/L/A
WIBR3 4i/L/A
WIBR3 5i/L/A
WIN1 5i/L/A
WIBR3 t2i/L/DOX+RI 12
WIBR3 t2i/L/DOX+RI 16
Primed WIBR1
Primed WIBR2
Primed WIBR3
Reprimed WIBR3 4i/L/A
Reprimed WIBR3 5i/L/A
Naive H9
Primed H9
8 cells
Morula
ICM
B
Genome-wide non-CpG methylation
mCpH/CpH
0.008
0.006
0.004
0.002
0.000
WIBR2 4i/L/A
WIBR3 4i/L/A
WIBR3 5i/L/A
WIN1 5i/L/A
WIBR3 DOX 12
WIBR3 DOX 16
Primed WIBR1
Primed WIBR2
Primed WIBR3
Re-primed WIBR3 4i/L/A
Re-primed WIBR3 5i/L/A
C
CpG Methylation
WIBR2 4i/L/A
WIBR3 4i/L/A
WIBR3 5i/L/A
WIN1 5i/L/A
WIBR3 DOX 12
WIBR3 DOX 16
Primed WIBR1
Primed WIBR2
Primed WIBR3
Re-primed WIBR3 4i/L/A
Re-primed WIBR3 5i/L/A
Naive H9 t2i/L+PKCi
Primed H9
ICM
mCpG/CpG
CGI
Promoter
Exon
Intron
Enhancer
Retroposon
Satellite
Simple repeat
SINE
LINE
LTR
D
non-CpG Methylation
WIBR2 4i/L/A
WIBR3 4i/L/A
WIBR3 5i/L/A
WIN1 5i/L/A
WIBR3 DOX 12
WIBR3 DOX 16
Primed WIBR1
Primed WIBR2
Primed WIBR3
Re-primed WIBR3 4i/L/A
Re-primed WIBR3 5i/L/A
mCpH/CpH
0.010
0.008
0.006
0.004
0.002
0.000
CGI
Promoter
Exon
Intron
Enhancer
Retroposon
Satellite
Simple repeat
SINE
LINE
LTR

Figure S4

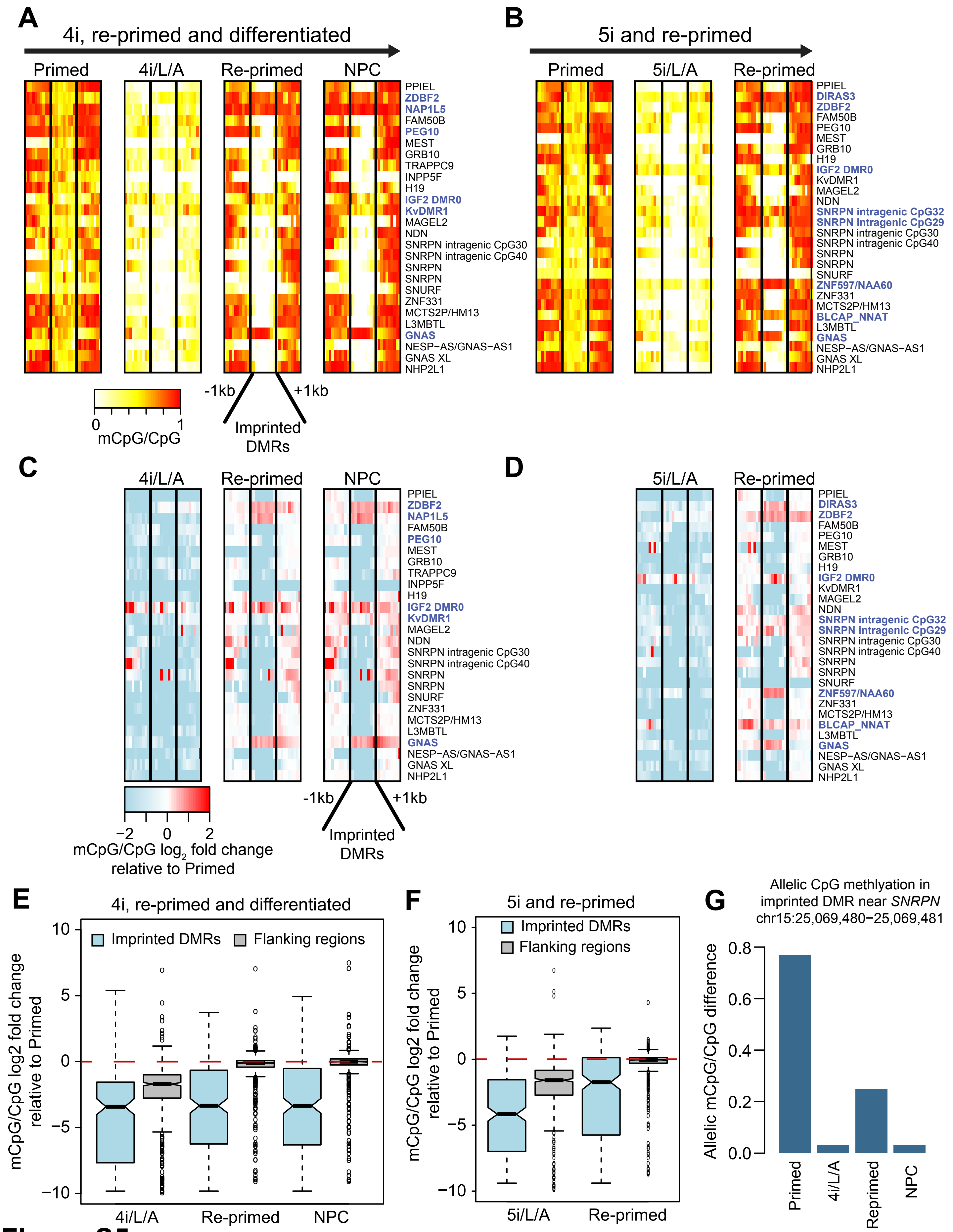
A
4i, re-primed and differentiated
Primed
4i/L/A
Re-primed
NPC
PPIEL
ZDBF2
NAP1L5
FAM50B
PEG10
MEST
GRB10
TRAPPC9
INPP5F
H19
IGF2 DMR0
KvDMR1
MAGEL2
NDN
SNRPN intragenic CpG30
SNRPN intragenic CpG40
SNRPN
SNRPN
SNURF
ZNF331
MCTS2P/HM13
L3MBTL
GNAS
NESP-AS/GNAS-AS1
GNAS XL
NHP2L1
0
1
mCpG/CpG
-1kb
+1kb
Imprinted DMRs
B
5i and re-primed
Primed
5i/L/A
Re-primed
PPIEL
DIRAS3
ZDBF2
FAM50B
PEG10
MEST
GRB10
H19
IGF2 DMR0
KvDMR1
MAGEL2
NDN
SNRPN intragenic CpG32
SNRPN intragenic CpG29
SNRPN intragenic CpG30
SNRPN intragenic CpG40
SNRPN
SNRPN
SNURF
ZNF597/NAA60
ZNF331
MCTS2P/HM13
BLCAP_NNAT
L3MBTL
GNAS
NESP-AS/GNAS-AS1
GNAS XL
NHP2L1
C
4i/L/A
Re-primed
NPC
−2
0
2
mCpG/CpG log2 fold change relative to Primed
-1kb
+1kb
Imprinted DMRs
D
5i/L/A
Re-primed
E
4i, re-primed and differentiated
Imprinted DMRs
Flanking regions
mCpG/CpG log2 fold change relative to Primed
10
5
0
−5
−10
4i/L/A
Re-primed
NPC
F
5i and re-primed
Imprinted DMRs
Flanking regions
5i/L/A
Re-primed
G
Allelic CpG methlyation in imprinted DMR near SNRPN chr15:25,069,480−25,069,481
Allelic mCpG/CpG difference
0.8
0.6
0.4
0.2
0.0
Primed
4i/L/A
Reprimed
NPC

Figure S5

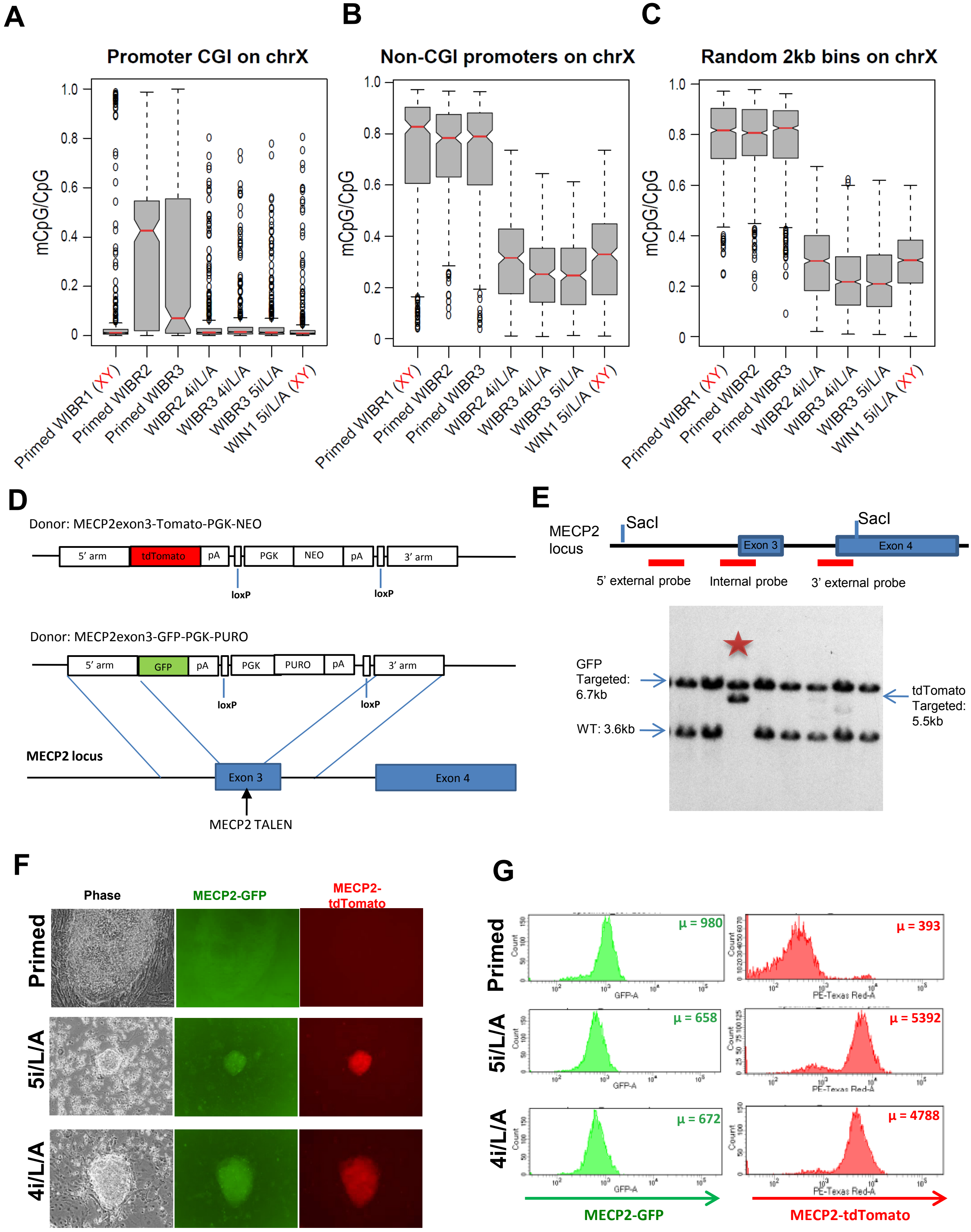

A
Promoter CGI on chrX
mCpG/CpG
1.0
0.8
0.6
0.4
0.2
0
Primed WIBR1 (XY)
Primed WIBR2
Primed WIBR3
WIBR2 4i/L/A
WIBR3 4i/L/A
WIBR3 5i/L/A
WIN1 5i/L/A (XY)
B
Non-CGI promoters on chrX
C
Random 2kb bins on chrX
D
Donor: MECP2exon3-Tomato-PGK-NEO
5' arm
tdTomato
pA
PGK
NEO
pA
3' arm
loxP
loxP
Donor: MECP2exon3-GFP-PGK-PURO
5' arm
GFP
pA
PGK
PURO
pA
3' arm
loxP
loxP
MECP2 locus
Exon 3
Exon 4
MECP2 TALEN
E
MECP2 locus
SacI
SacI
Exon 3
Exon 4
5' external probe
Internal probe
3' external probe
GFP Targeted: 6.7kb
WT: 3.6kb
tdTomato Targeted: 5.5kb
F
Phase
MECP2-GFP
MECP2-tdTomato
Primed
5i/L/A
4i/L/A
G
Primed
5i/L/A
4i/L/A
μ = 980
μ = 393
μ = 658
μ = 5392
μ = 672
μ = 4788
Count
GFP-A
PE-Texas Red-A
MECP2-GFP
MECP2-tdTomato

Figure S6

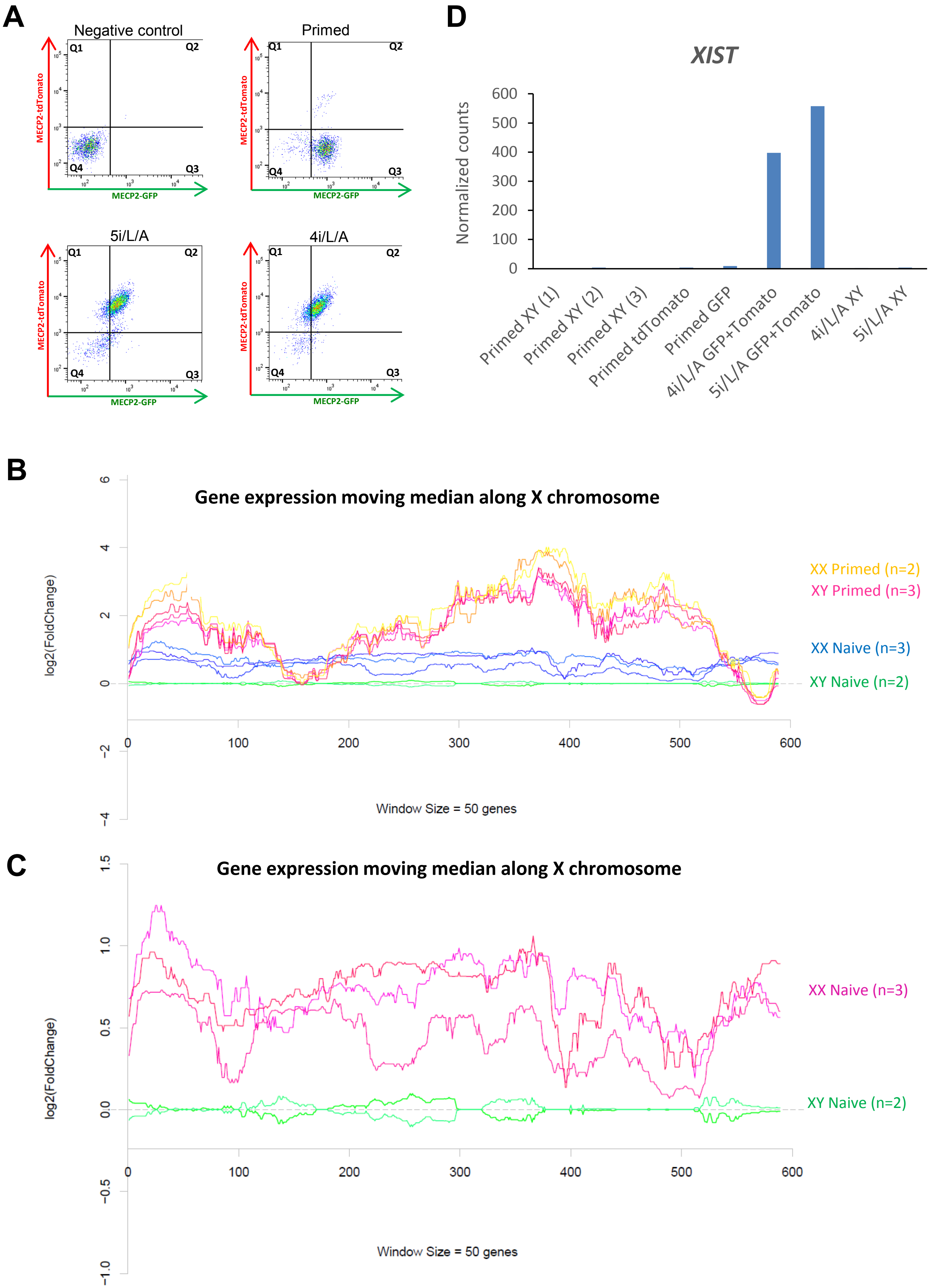
A
Negative control
Primed
5i/L/A
4i/L/A
Q1
Q2
Q3
Q4
MECP2-tdTomato
MECP2-GFP
D
XIST
Normalized counts
600
500
400
300
200
100
0
Primed XY (1)
Primed XY (2)
Primed XY (3)
Primed tdTomato
Primed GFP
4i/L/A GFP+Tomato
5i/L/A GFP+Tomato
4i/L/A XY
5i/L/A XY
B
Gene expression moving median along X chromosome
log2(FoldChange)
XX Primed (n=2)
XY Primed (n=3)
XX Naive (n=3)
XY Naive (n=2)
Window Size = 50 genes
C
Gene expression moving median along X chromosome
log2(FoldChange)
XX Naive (n=3)
XY Naive (n=2)
Window Size = 50 genes

Figure S7

V. Supplemental References

Bai, Q., Assou, S., Haouzi, D., Ramirez, J.M., Monzo, C., Becker, F., Gerbal-Chaloin, S., Hamamah, S., and De Vos, J. (2012). Dissecting the first transcriptional divergence during human embryonic development. Stem Cell Rev *8*, 150-162.

Balaton, B.P., Cotton, A.M., and Brown, C.J. (2015). Derivation of consensus inactivation status for X-linked genes from genome-wide studies. Biology of sex differences *6*, 35.

Benjamini, Y.a.H., Y. (1995). Controlling the false discovery rate: A practical and powerful approach to multiple testing. Journal of the Royal Statistical Society B.

Cohen, M.A., Itsykson, P., and Reubinoff, B.E. (2007). Neural differentiation of human ES cells. Curr Protoc Cell Biol *Chapter 23*, Unit 23 27.

Cohen, M.A., Wert, K.J., Goldmann, J., Markoulaki, S., Buganim, Y., Fu, D., and Jaenisch, R. (2016). Human neural crest cells contribute to coat pigmentation in interspecies chimeras after in utero injection into mouse embryos. Proceedings of the National Academy of Sciences of the United States of America.

Court, F., Tayama, C., Romanelli, V., Martin-Trujillo, A., Iglesias-Platas, I., Okamura, K., Sugahara, N., Simon, C., Moore, H., Harness, J.V.*, et al.* (2014). Genome-wide parent-of-origin DNA methylation analysis reveals the intricacies of human imprinting and suggests a germline methylation-independent mechanism of establishment. Genome research *24*, 554-569.

Dobin, A., Davis, C.A., Schlesinger, F., Drenkow, J., Zaleski, C., Jha, S., Batut, P., Chaisson, M., and Gingeras, T.R. (2013). STAR: ultrafast universal RNA-seq aligner. Bioinformatics *29*, 15-21.

Edgar, R., Domrachev, M., and Lash, A.E. (2002). Gene Expression Omnibus: NCBI gene expression and hybridization array data repository. Nucleic Acids Res *30*, 207-210.

Gafni, O., Weinberger, L., Mansour, A.A., Manor, Y.S., Chomsky, E., Ben-Yosef, D., Kalma, Y., Viukov, S., Maza, I., Zviran, A.*, et al.* (2013). Derivation of novel human ground state naive pluripotent stem cells. Nature *504*, 282-286.

Gentleman, R.C., Carey, V.J., Bates, D.M., Bolstad, B., Dettling, M., Dudoit, S., Ellis, B., Gautier, L., Ge, Y., Gentry, J., *et al.* (2004). Bioconductor: open software development for computational biology and bioinformatics. Genome Biol *5*, R80.

Guo, H., Zhu, P., Yan, L., Li, R., Hu, B., Lian, Y., Yan, J., Ren, X., Lin, S., Li, J., *et al.* (2014). The DNA methylation landscape of human early embryos. Nature *511*, 606-610.

Harrow, J., Frankish, A., Gonzalez, J.M., Tapanari, E., Diekhans, M., Kokocinski, F., Aken, B.L., Barrell, D., Zadissa, A., Searle, S., *et al.* (2012). GENCODE: the reference human genome annotation for The ENCODE Project. Genome research *22*, 1760-1774.

Langmead, B., and Salzberg, S.L. (2012). Fast gapped-read alignment with Bowtie 2. Nat Methods *9*, 357-359.

Law, C.W., Chen, Y., Shi, W., and Smyth, G.K. (2014). voom: Precision weights unlock linear model analysis tools for RNA-seq read counts. Genome Biol *15*, R29.

Lengner, C.J., Gimelbrant, A.A., Erwin, J.A., Cheng, A.W., Guenther, M.G., Welstead, G.G., Alagappan, R., Frampton, G.M., Xu, P., Muffat, J., *et al.* (2010). Derivation of pre-X inactivation human embryonic stem cells under physiological oxygen concentrations. Cell *141*, 872-883.

Li, H. (2011). A statistical framework for SNP calling, mutation discovery, association mapping and population genetical parameter estimation from sequencing data. Bioinformatics *27*, 2987-2993.

Maetzel, D., Sarkar, S., Wang, H., Abi-Mosleh, L., Xu, P., Cheng, A.W., Gao, Q., Mitalipova, M., and Jaenisch, R. (2014). Genetic and chemical correction of cholesterol accumulation and impaired autophagy in hepatic and neural cells derived from Niemann-Pick Type C patient-specific iPS cells. Stem cell reports *2*, 866-880.

Martin, M. (2011). Cutadapt removes adapter sequences from high-throughput sequencing reads. EMBnetjournal *17*.

Roberts, A., Trapnell, C., Donaghey, J., Rinn, J.L., and Pachter, L. (2011). Improving RNA-Seq expression estimates by correcting for fragment bias. Genome Biol *12*, R22.

Schultz, M.D., He, Y., Whitaker, J.W., Hariharan, M., Mukamel, E.A., Leung, D., Rajagopal, N., Nery, J.R., Urich, M.A., Chen, H., *et al.* (2015). Human body epigenome maps reveal noncanonical DNA methylation variation. Nature *523*, 212-216.

Schultz, M.D., Schmitz, R.J., and Ecker, J.R. (2012). 'Leveling' the playing field for analyses of single-base resolution DNA methylomes. Trends Genet *28*, 583-585.

Shen, L., Shao, N., Liu, X., and Nestler, E. (2014). ngs.plot: Quick mining and visualization of next-generation sequencing data by integrating genomic databases. BMC genomics *15*, 284.

Soldner, F., Laganiere, J., Cheng, A.W., Hockemeyer, D., Gao, Q., Alagappan, R., Khurana, V., Golbe, L.I., Myers, R.H., Lindquist, S., *et al.* (2011). Generation of isogenic pluripotent stem cells differing exclusively at two early onset Parkinson point mutations. Cell *146*, 318-331.

Takashima, Y., Guo, G., Loos, R., Nichols, J., Ficz, G., Krueger, F., Oxley, D., Santos, F., Clarke, J., Mansfield, W., *et al.* (2014). Resetting transcription factor control circuitry toward ground-state pluripotency in human. Cell *158*, 1254-1269.

Theunissen, T.W., Powell, B.E., Wang, H., Mitalipova, M., Faddah, D.A., Reddy, J., Fan, Z.P., Maetzel, D., Ganz, K., Shi, L., *et al.* (2014). Systematic identification of culture conditions for induction and maintenance of naive human pluripotency. Cell stem cell *15*, 471-487.

Wang, J., Xie, G., Singh, M., Ghanbarian, A.T., Rasko, T., Szvetnik, A., Cai, H., Besser, D., Prigione, A., Fuchs, N.V., *et al.* (2014). Primate-specific endogenous retrovirus-driven transcription defines naive-like stem cells. Nature *516*, 405-409.

Wang, T., Liu, J., Shen, L., Tonti-Filippini, J., Zhu, Y., Jia, H., Lister, R., Whitaker, J.W., Ecker, J.R., Millar, A.H., *et al.* (2013). STAR: an integrated solution to management and visualization of sequencing data. Bioinformatics *29*, 3204-3210.

Xie, W., Schultz, M.D., Lister, R., Hou, Z., Rajagopal, N., Ray, P., Whitaker, J.W., Tian, S., Hawkins, R.D., Leung, D., *et al.* (2013). Epigenomic analysis of multilineage differentiation of human embryonic stem cells. Cell *153*, 1134-1148.

Yan, L., Yang, M., Guo, H., Yang, L., Wu, J., Li, R., Liu, P., Lian, Y., Zheng, X., Yan, J., *et al.* (2013). Single-cell RNA-Seq profiling of human preimplantation embryos and embryonic stem cells. Nat Struct Mol Biol *20*, 1131-1139.
